# Trash or treasure? Unlocking dark matter of enantiomeric natural products in innovative drugs discovery for potent angiogenesis inhibitors

**DOI:** 10.1007/s42995-025-00307-8

**Published:** 2025-07-10

**Authors:** Yan-Wei Wu, Xiao-Feng Mou, Zhong-Yuan Chen, Xiao-Jia Xue, Wen-Hui Wang, Jin-Zhou Guo, Bo-Qi Zhang, Ting-Ting Xue, Qun Zhang, Mei-Yan Wei, Yu-Cheng Gu, Gulab Said, Chang-Yun Wang, Ling Lu, Chang-Lun Shao

**Affiliations:** 1 Key Laboratory of Marine Drugs, The Ministry of Education of China, School of Medicine and Pharmacy, Ocean University of China, Qingdao, 266003 China; 2 Syngenta Jealott’s Hill International Research Centre, Bracknell, Berkshire, RG42 6EY UK; 3 Department of Chemistry, Women University Swabi, Swabi, 23430 Pakistan; 4 Key Laboratory of Tropical Medicinal Resource Chemistry of Ministry of Education, College of Chemistry and Chemical Engineering, Hainan Normal University, Haikou, 571158 China

**Keywords:** Angiogenesis, Chirality, Chiral natural products, Enantiomers, Dark matter of enantiomeric natural products, Innovative drugs

## Abstract

The occurrence and development of tumors rely on the nutritional supply from blood vessels, which also serve as the main pathway for tumor metastasis. Inhibiting angiogenesis is one of the main strategies for cancer treatments. Chiral drugs, encouraged and inspired by chiral natural products, make up a major portion of marketed drugs. However, as an important source for synthetic chemistry and drug discovery, the counterpart of chiral natural products, the enantiomers, has received little attention. Here, we constructed a compound library containing 100 racemates ( ±)-**1**–**100** and 4 pairs of enantiomers (**33**, **48**, **59**, **68**) of 3,4-dioxygenated-4-aryl-quinolin-2(1*H*)-one alkaloids. Through extensive activity screening, we found that the compounds with 3*R*, 4*R* configuration, opposite to the natural products, exhibited potent angiogenesis inhibitory activity in zebrafish, while the 3*S*, 4*S*-configured natural derivatives have no effects. More importantly, compound ( +)-**48**, named as (3*R*, 4*R*)-CHNQD-00728, significantly inhibited hepatic tumor growth in doxycin hydrochloride-induced liver-specific enlargement zebrafish. Examining the phenomenon and unlocking dark matter of enantiomeric natural products in innovative drugs discovery can provide a new perspective on organic synthesis and medicinal chemistry, thus enabling a broader exploration.

**Supplementary Information:**

The online version contains supplementary material available at 10.1007/s42995-025-00307-8.

## Introduction

**Fig. 1:**
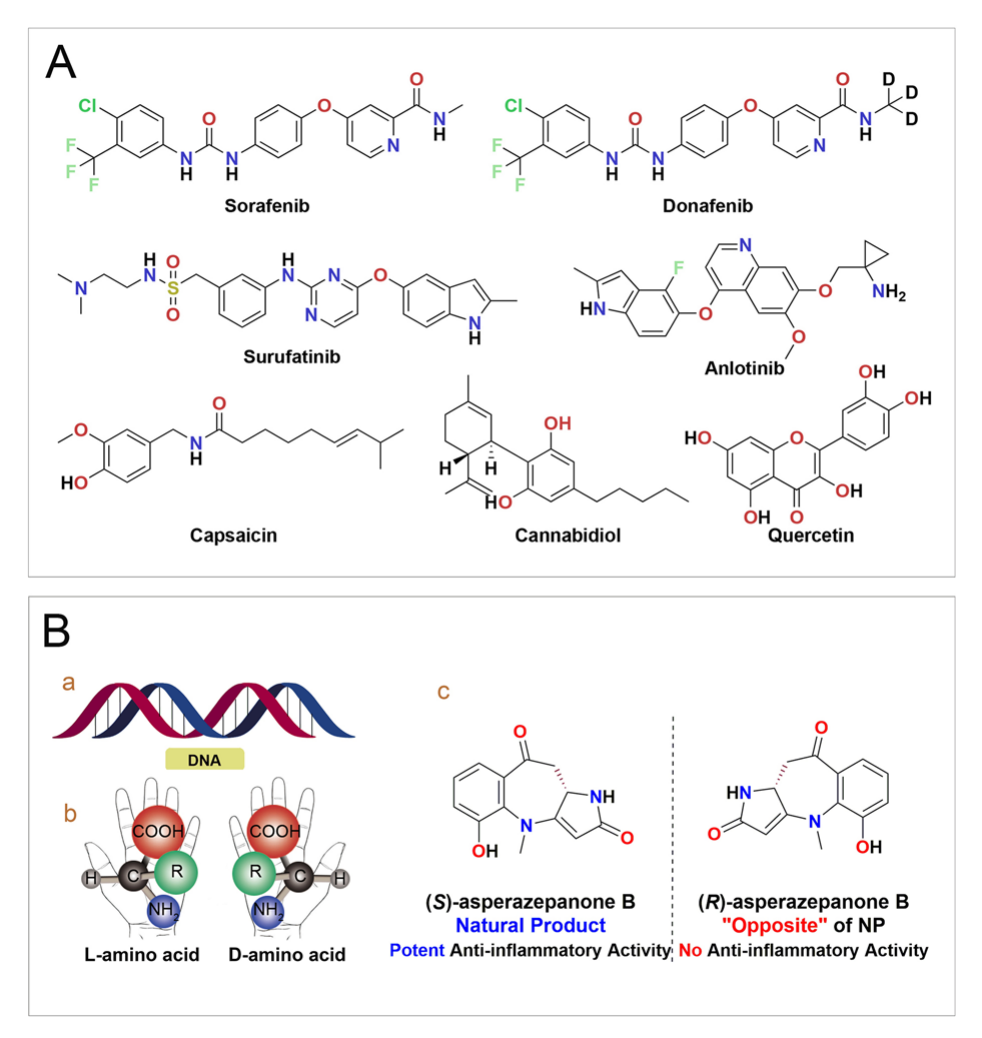
Anti-angiogenic small molecules and chirality in nature. **A** Structure of anti-angiogenic small molecules that are approved or potential drugs. **B** (a) Structure of DNA; (b) *L* and *D*-amino acid; (c) A pair of enantiomers: (*S*)-asperazepanone B and its enantiomer (*R*)-asperazepanone B)

Angiogenesis is an essential process in the normal development and physiology of the body, strictly regulated by a variety of pro-angiogenic and vaso-inhibitory signals (Cao et al. 2023; Carmeliet 2005; Dudley and Griffioen 2023). However, the accumulating evidence underscores the intricate association of angiogenic processes with many pathological conditions. An unbalanced expression of angiogenic factors can lead to the formation of aberrant vascular networks, that ultimately result in tissue and organ dysfunction leading to disease states which encompass malignancies, impaired wound healing, diabetic retinopathy, and beyond. Efforts in this area of research are yielding an increasing number of anti-angiogenic molecules as approved drugs or potential drugs (Azevedo et al. 2024; Li et al. 2021; Vafopoulou and Kourti 2022) (Fig. 1A).

Chirality plays a vital role in nature and living systems (Bitchagno et al. 2022; Brandt et al. 2017). The main substances that constitute living organisms, proteins and nucleic acids have certain stereo-configurations, and the interaction of these molecules with different enantiomers during the formation and evolution of life creates a preference for a particular enantiomer (Liu et al. 2023a, b; Niu et al. 2023) (Fig. 1B). From a biosynthetic point of view, as enzymes are chiral molecules, the enzymatic reactions generating natural products (NPs) are also stereospecific, and lead to single enantiomer NPs (Bitchagno et al. 2022), namely chiral NPs. As secondary metabolites, chiral natural products, with novel and complex structures and diverse biological activities (Carroll et al. 2024; Newman and Cragg 2020; Newman 2022), do not play an important role in maintaining the basic life activities of organisms, whereas can act as a chemical defense when interacting with the environment or other species, thereby enhancing the entity’s survival chances (Atanasov et al. 2021; Bitchagno et al. 2022; Mullowney et al. 2023).

The chiral characteristics of biological entities makes drug molecules prone to possess specific chiral structures (Brandt et al. 2017; Shen et al. 2013). Correspondingly, chiral drugs accounted for approximately two-thirds of marketed drugs over the past two decades (from 2002 to 2022) (McVicker and O’Boyle 2024) and 7 out of the top 10 best-selling drugs globally were chiral drugs, in 2023 (McGrath et al. 2010). From those, chiral compounds are of undeniable importance in continuing to drive drug development for a range of diseases (Brooks et al. 2011; Liu et al. 2023a, b). However, chiral natural products are generally found in low levels in nature, which greatly hinders the development and utilization. To address the issue of drug source limitations, researchers, especially synthetic chemists, have been tireless in developing novel and highly advanced technological limits in pursuit of chirality, striving to obtain the target chiral molecules with 100% selectivity and 100% yield without waste generation, thus realizing green and efficient chiral-controlled total synthesis (Ahrendt et al. 2000; Jang et al. 2007; Maji et al. 2023; Nicolaou et al. 2000; Nicolaou 2012). However, the application of precise chiral controlled total synthesis approach to chiral natural product synthesis is restricted. In the process of total synthesis, enantiomers that are not identical or completely different from the configuration of the NPs (when chiral control cannot be achieved), are readily produced and are often considered “waste”. For example, (*S*)-asperazepanone B, as shown in Fig. 1Bc, the marine-derived natural product possessed potent anti-inflammatory activity, whereas its synthetic enantiomer (*R*)-asperazepanone B is completely inactive (Xu et al. 2022). This may be the reason why many researchers focus solely on the chiral natural products themselves. Nevertheless, this could lead to the neglect of half of the molecules, enantiomeric natural products which may have remarkable activity. If we can fully explore and utilize the “waste” or “ineffective” enantiomers (a class of functional dark matter) generated during the total synthesis process, this can not only turn waste into treasure, but also greatly expand the drug resource pool.

We have been dedicated to the discovery of marine natural products (MNPs) and their mechanisms of action (Chen et al. 2023; Han et al. 2023; Hou et al. 2019; Jiang et al. 2022; Liu et al. 2024; Xu et al. 2021b), and have constructed a compound library containing more than 2300 molecules of MNPs and their derivatives. Moreover, we have been focusing on NPs and their isomers, with an emphasis on the differences in biological activity (Jia et al. 2015; Xu et al. 2022, 2021a). In the previous study, we obtained a series of chiral 3,4-dioxygenated-4-aryl-quinolin-2(1*H*)-one alkaloids from marine-derived fungi, which have excellent anti-fouling, antiviral and anti-inflammatory activities (Guo et al. 2023b; Qu et al. 2023; Shao et al. 2015, 2020). In particular, the total synthesis of aniduquinolone A and stereoisomers have been achieved after years of exploration (Guo et al. 2022).

In this study, to explore the potential relationship between the chiral centers of 3,4-dioxygenated-4-aryl-quinolin-2(1*H*)-one derivatives and their activity, we synthesized the racemic analogues of 3,4-dioxygenated-4-aryl-quinolin-2(1*H*)-one and obtained the enantiomers by high-performance liquid chromatography. Ultimately, a compound library composed of 3,4-dioxygenated-4-aryl-quinolin-2(1*H*)-one racemates and enantiomers (**1**–**100**) was constructed and subjected to extensive activity screening through multiple activity screening platforms.

It was unexpected that the compounds with *R*, *R* configuration can inhibit angiogenic activity in zebrafish, while the corresponding natural products with *S*, *S*-configured, were ineffective. Specifically, compound ( +)-**33**, named as (3*R*, 4*R*)-CHNQD-00610, had a significant antiangiogenic inhibitory effect, while the (−)-**33**, namely (3*S*, 4*S*)-CHNQD-00610, was inactive, for the first time. Furthermore, structural modification and activity assessment resulted in the most active one, ( +)-**48**, namely (3*R*, 4*R*)-CHNQD-00728, which proved to be effective in vivo and in vitro.

## Material and methods

### Chemistry

#### General procedures for the synthesis of *Route 1*

Under N_2_ protection, benzophenone **I** (0.25 mmol) was dissolved in DCM (5 mL), and methoxyacetyl chloride (0.5 mmol) was added under the catalysis of K_2_CO_3_, and reacted at room temperature for 0.5 h, then quenched with water, and extracted with EtOAc. The solvent was evaporated under reduced pressure to remove the solvent to get the compound **III**. After drying, **III** was dissolved in THF (10 mL), and *t*-BuOK (2.5 mmol) was added and reacted at room temperature for 5 h. The reaction mixture was extracted with saturated aqueous NH_4_Cl and EtOAc. After the solvent was evaporated under reduced pressure, the compound **IV** was purified by silica gel column chromatography.

#### General procedures for the synthesis of *Route 2*

After dissolving benzamide (13.2 mmol) in anhydrous pyridine (20 mL) at room temperature, methoxyacetyl chloride (26.5 mmol) was slowly added dropwise under continuous stirring. The mixture was then heated to 60 °C for 2 h. The reaction was monitored by TLC until it was complete, then the reaction was stopped and cooled to room temperature. After removing the organic solvent by distillation under reduced pressure, the resulting mixture was diluted with saturated aqueous NH_4_Cl and EtOAc. The organic layer was separated and the aqueous layer was extracted with EtOAc. The resulting organic extract was washed with brine, dried over anhydrous Na_2_SO_4_, concentrated by filtration under reduced pressure and purified by silica gel column chromatography to give the corresponding aryl imide product **II-3**.

HMDS (1.0 mmol) was added to a THF solution (5 mL) of raw phenol **II** (0.5 mmol) at room temperature. The mixture was reacted at 70 °C for 10 h, cooled to room temperature, distilled under reduced pressure to remove all volatile compounds, and dried to give TMS ether **II-1**. The concentrate was dissolved in dry THF (10 mL) at − 78 °C, and stirring was continued for 50 min with the slow dropwise addition of *n*-BuLi (3.0 mmol) under N_2_ protection, followed by the dropwise addition of Tf_2_O (1.5 mmol) and continued stirring for 50 min. The reaction was stopped by TLC and quenched by saturated aqueous Na_2_CO_3_ (50 mL) at − 78 °C and EtOAc was added. After separation of the aqueous phase and EtOAc, the aqueous phase was extracted with EtOAc. The resulting organic phase was concentrated under reduced pressure. The mixture was purified by silica gel column chromatography to give the target compound **II-2** in oil form.

Compound **II-2** (0.5 mmol) was dissolved in MeCN solution (8 mL) at room temperature, followed by addition of aryl imide **II-3** (1.8 mmol), and CsF (2.5 mmol). The reaction system was reacted at 80 °C for 2 h. After the reaction was monitored for completeness by TLC, the reaction system was cooled to room temperature and the solvent was evaporated under reduced pressure. Intermediate **III** was purified by silica gel column chromatography.

#### Synthesis of 3,4-dioxygenated-4-aryl-quinolin-2(1*H*)-one alkaloid derivatives

Under N_2_ protection, compound **IV** (0.37 mmol) was dissolved in dry MeCN (5 mL) and benzyl bromide (1.12 mmol) was added with the catalysis of K_2_CO_3_. The reaction was carried out for 4 h at 60 °C, and the end of the reaction was detected by TLC. The solvent was removed under reduced pressure and the reaction mixture was partitioned between H_2_O and EtOAc and the aqueous layer extracted with EtOAc. The residue was purified by silica gel column chromatography to give compound **V**.

### Preparation of compounds 23–75

Under N_2_ protection, compound **IV** (0.37 mmol) was dissolved in dry MeCN (5 mL) and benzyl bromide (1.12 mmol) was added with the catalysis of K_2_CO_3_. The reaction was carried out for 4 h at 60 °C, and the end of the reaction was detected by TLC. The solvent was evaporated under reduced pressure and the reaction mixture was partitioned between H_2_O and EtOAc and the aqueous layer extracted with EtOAc (3 × 120 mL). The residue was purified by silica gel column chromatography to give compounds **23–75**. Characterization data of the core compounds are shown below, and data of other compounds can be found in supporting information.

4-Hydroxy-3-methoxy-1-(3-methylbenzyl)-4-phenyl-3,4-dihydroquinolin-2(1*H*)-one (**33**), white solid, 52% yield. ( +)-**33**, [α]^16.3^ D =  + 56.7° (*c* 0.5, MeOH); (−)-**33**, [α]^16.3^ D =− 97.9° (*c* 0.5, MeOH); ^1^H NMR (500 MHz, acetone-*d*_6_) *δ* 7.37 (1H, d, *J* = 7.6 Hz), 7.32 (5H, overlapped), 7.25 (1H, t, *J* = 7.8 Hz), 7.17 (1H, t, *J* = 7.8 Hz), 7.08–7.04 (5H, overlapped), 5.22 (1H, d, *J* = 16.2 Hz), 5.11 (1H, d, *J* = 16.2 Hz), 4.81 (1H, s), 4.24 (1H, s), 3.52 (3H, s), 2.26 (3H, s); ^13^C NMR (125 MHz, acetone-*d*_6_) *δ* 168.1, 142.3, 139.0, 138.8, 137.9, 131.8, 129.6, 129.2, 129.0 × 2, 128.7, 128.5, 128.3, 128.1, 127.9 × 2, 124.7, 123.9, 116.1, 85.7, 77.0, 59.4, 45.5, 21.4; ESIMS *m/z* 374.20 [M + H]^+^, 396.20 [M + Na]^+^; HRESIMS *m/z* 374.1743 [M + H]^+^ (calcd for C_24_H_24_NO_3_^+^, 374.1751).

7-Fluoro-4-hydroxy-3-methoxy-1-(3-methylbenzyl)-4-phenyl-3,4-dihydroquinolin-2(1*H*)-one (**47**), white solid, 52% yield. ^1^H NMR (400 MHz, CDCl_3_) *δ* 7.23–7.17 (4H, overlapped), 7.16–7.10 (2H, overlapped), 7.08 (1H, t, *J* = 7.5 Hz), 6.96 (1H, d, *J* = 7.6 Hz), 6.87–6.81 (4H, overlapped), 5.07 (1H, d, *J* = 16.2 Hz), 4.93 (1H, d, *J* = 16.2 Hz), 4.04 (1H, s), 3.51 (4H, s), 2.19 (3H, s); ^13^C NMR (100 MHz, CDCl_3_) *δ* 166.4, 159.3 (d, *J* = 242.6 Hz), 138.9, 138.4, 135.8, 133.6 (d, *J* = 2.6 Hz), 132.4 (d, *J* = 6.8 Hz), 128.7, 128.6 × 3, 128.1, 127.1, 126.8 × 2, 123.6, 116.7 (d, *J* = 7.9 Hz), 115.5 (d, *J* = 22.7 Hz), 114.8 (d, *J* = 24.3 Hz), 84.3, 75.8, 59.2, 45.7, 21.4; HRESIMS m/z 392.1653 [M + H]^+^ (calcd for C_24_H_23_O_3_NF^+^, 392.1656); 414.1472 [M + Na]^+^ (calcd for C_24_H_22_O_3_NFNa^+^, 414.1476); 374.1548 [M-H_2_O + H]^+^ (calcd for C_24_H_21_FNO_2_^+^, 374.1551).

7-Chloro-4-hydroxy-3-methoxy-1-(3-methylbenzyl)-4-phenyl-3,4-dihydroquinolin-2(1*H*)-one (**48**), white solid, 63% yield. ( +)-**48**, [α]^16.3^ D =  + 67.8° (*c* 0.2, MeOH); (−)-**48**, [α]^16.3^ D =− 34.4° (*c* 0.2, MeOH); ^1^H NMR (600 MHz, CDCl_3_) *δ* 7.56 (1H, d, *J* = 2.5 Hz), 7.33–7.28 (3H, overlapped), 7.23–7.16 (4H, overlapped), 7.06 (1H, d, *J* = 7.6 Hz), 6.94 (1H, d, *J* = 7.8 Hz), 6.91 (2H, d, *J* = 8.8 Hz), 5.16 (1H, d, *J* = 16.1 Hz), 5.04 (1H, d, *J* = 16.1 Hz), 4.11 (1H, s), 3.61 (3H, s), 3.54 (1H, s), 2.28 (3H, s); ^13^C NMR (150 MHz, CDCl_3_) *δ* 166.43, 138.9, 138.5, 136.1, 135.7, 132.0, 129.5, 129.0, 128.8, 128.7 × 2, 128.6, 128.2, 127.7, 127.1, 126.8 × 2, 123.7, 116.6, 84.3, 75.8, 59.2, 45.6, 21.4; HRESIMS *m*/*z* 408.1369 [M + H]^+^ (calcd for C_24_H_23_ClNO_3_^+^, 408.1361).

7-Chloro-4-hydroxy-3-methoxy-1-(2-methylbenzyl)-4-phenyl-3,4-dihydroquinolin-2(1*H*)-one (**59**), white solid, 53%. ( +)-**59**, [α]^16.3^ D =  + 100.4° (*c* 0.5, MeOH); (−)-**59**, [α]^16.3^ D =− 78.3° (*c* 0.8, MeOH); ^1^H NMR (600 MHz, CDCl_3_) *δ* 7.60 (1H, d, *J* = 2.5 Hz), 7.38–7.33 (3H, overlapped), 7.31–7.28 (2H, overlapped), 7.23–7.16 (3H, overlapped), 7.09 (1H, t, *J* = 7.5 Hz), 6.84 (1H, d, *J* = 7.7 Hz), 6.70 (1H, d, *J* = 8.7 Hz), 5.40 (1H, d, *J* = 16.9 Hz), 4.69 (1H, d, *J* = 16.9 Hz), 4.13 (1H, s), 3.61 (3H, s), 3.60 (1H, s), 2.38 (3H, s); ^13^C NMR (150 MHz, CDCl_3_) *δ* 166.3, 138.9, 136.5, 134.9, 133.2, 131.9, 130.5, 129.5, 129.1, 128.8, 128.7 × 2, 127.7, 127.2, 126.7 × 2, 126.3, 124.1, 116.6, 84.3, 75.8, 59.2, 44.2, 19.1; HRESIMS *m*/*z* 408.1368 [M + H]^+^ (calcd for C_24_H_23_ClNO_3_^+^, 408.1361).

7-Chloro-4-hydroxy-3-methoxy-4-phenyl-1-(3-(trifluoromethyl)benzyl)-3,4-dihydroquinolin-2(1*H*)-one (**61**), white solid, 58% yield. ^1^H NMR (500 MHz, acetone-*d*_6_) *δ* 7.61 (2H, d, *J* = 11.9 Hz), 7.54 (1H, t, *J* = 7.6 Hz), 7.49 (1H, d, *J* = 7.8 Hz), 7.36–7.34 (6H, overlapped), 7.30 (1H, dd, *J* = 8.7, 2.5 Hz), 7.14 (1H, d, *J* = 8.7 Hz), 5.43 (1H, d, *J* = 16.6 Hz), 5.19 (1H, d, *J* = 16.6 Hz), 5.13 (1H, s), 4.30 (1H, s), 3.52 (3H, s); ^13^C NMR (125 MHz, acetone-*d*_6_) *δ* 168.1, 141.3 (d, *J* = 6.0 Hz), 139.2, 137.6, 134.1 (d, *J* = 4.1 Hz), 131.5, 131.2 (d, *J* = 31.9 Hz), 130.4, 129.5, 129.2 × 2, 129.1 × 2, 128.3, 127.7 × 2, 125.2 (dd, *J* = 379.4, 109.2 Hz), 124.9 (dd, *J* = 7.7, 3.8 Hz), 124.2 (dd, *J* = 7.4, 3.5 Hz), 117.7, 85.2, 76.9, 59.5, 45.3; ESIMS *m/z* 462.12/464.07 [M + H]^+^/[M + 2 + H]^+^ (3:1); HRESIMS *m/z* 462.1071 [M + H]^+^ (calcd for C_24_H_20_F_3_ClNO_3_^+^, 462.1078).

7-Chloro-4-hydroxy-3-methoxy-1-(4-methylbenzyl)-4-phenyl-3,4-dihydroquinolin-2(1*H*)-one (**68**), white solid, 55% yield. ( +)-**68**, [α]^16.3^ D =  + 42.9° (*c* 0.2, MeOH); (−)-**68**, [α]^16.3^ D =−44.3° (*c* 0.2, MeOH); ^1^H NMR (500 MHz, acetone-*d*_6_) *δ* 7.38–7.25 (7H, overlapped), 7.11 (5H, overlapped), 5.18 (1H, d, *J* = 16.1 Hz), 5.11 (1H, d, *J* = 16.1 Hz), 5.04 (1H, s), 4.24 (1H, s), 3.51 (3H, s), 2.28 (3H, s); ^13^C NMR (125 MHz, acetone-*d*_6_) *δ* 167.8, 141.4, 137.8, 137.5, 134.4, 134.2, 130.0 × 2, 129.3, 129.1 × 2, 129.1, 128.8, 128.1, 127.8 × 2, 127.7 × 2, 118.0, 85.4, 76.8, 59.4, 45.3, 21.0; ESIMS *m/z* 408.17/410.16 [M + H]^+^/[M + 2 + H]^+^ (3:1); HRESIMS *m/z* 408.1355 [M + H]^+^ (calcd for C_24_H_23_ClNO_3_^+^, 408.1361).

## Preparation of compounds 76–100

Under nitrogen protection, compound **IV**^3,4 Δ^ (0.35 mmol) was dissolved in dry MeCN (5 mL) and benzyl bromide (1.05 mmol) was added with the catalysis of K_2_CO_3_. The reaction was carried out for 4 h at 60 °C, and the end of the reaction was detected by TLC. The solvent was evaporated under reduced pressure and the reaction mixture was partitioned between H_2_O and EtOAc and the aqueous layer extracted with EtOAc (3 × 120 mL). The residue was purified by silica gel column chromatography to give compounds **76–100**.

3-Methoxy-1-(3-methylbenzyl)-4-phenylquinolin-2(1*H*)-one (**80**), white solid, 55% yield. ^1^H NMR (500 MHz, acetone-*d*_6_) *δ* 7.59–7.53 (2H, overlapped), 7.50 (1H, t, *J* = 7.4 Hz), 7.46 (1H, d, *J* = 8.3 Hz), 7.41–7.39 (3H, overlapped), 7.23–7.20 (2H, overlapped), 7.16 (1H, dd, *J* = 8.0, 1.4 Hz), 7.12–7.09 (2H, overlapped), 7.08 (1H, d, *J* = 7.5 Hz), 5.66 (2H, s), 3.82 (3H, s), 2.29 (3H, s); ^13^C NMR (125 MHz, acetone-*d*_6_) *δ* 159.7, 145.8, 139.1, 138.1, 137.9, 137.6, 134.9, 130.3 × 2, 129.5, 129.4, 129.2 × 2, 128.9, 128.7, 128.3, 127.8, 124.7, 123.0, 122.1, 116.0, 60.1, 46.4, 21.4; ESIMS *m/z* 356.13 [M + H]^+^, 378.15 [M + Na]^+^.

7-Chloro-3-methoxy-1-(3-methylbenzyl)-4-phenylquinolin-2(1*H*)-one (**94**), white solid, 53% yield. ^1^H NMR (500 MHz, acetone-*d*_6_) *δ* 7.61–7.56 (2H, overlapped), 7.55–7.50 (1H, m), 7.47 (1H, d, *J* = 9.0 Hz), 7.42 (1H, d, *J* = 1.6 Hz), 7.42–7.37 (2H, overlapped), 7.22 (1H, t, *J* = 7.6 Hz), 7.19 (1H, s), 7.12–7.07 (3H, overlapped), 5.65 (2H, s), 3.84 (3H, s), 2.29 (3H, s); ^13^C NMR (125 MHz, acetone-*d*_*6*_,) *δ* 159.4, 146.8, 139.1, 137.5, 136.9, 136.2, 134.2, 130.3 × 2, 129.5, 129.4 × 2, 129.2 × 2, 128.8, 128.2, 128.0, 126.6, 124.6, 123.7, 117.9, 60.2, 46.6, 21.4; HRESIMS *m*/*z* 390.1262 [M + H]^+^ (calcd for C_24_H_21_ClNO_2_^+^, 390.1255).

## Results and discussion

### Chemistry

**Fig. 2:**
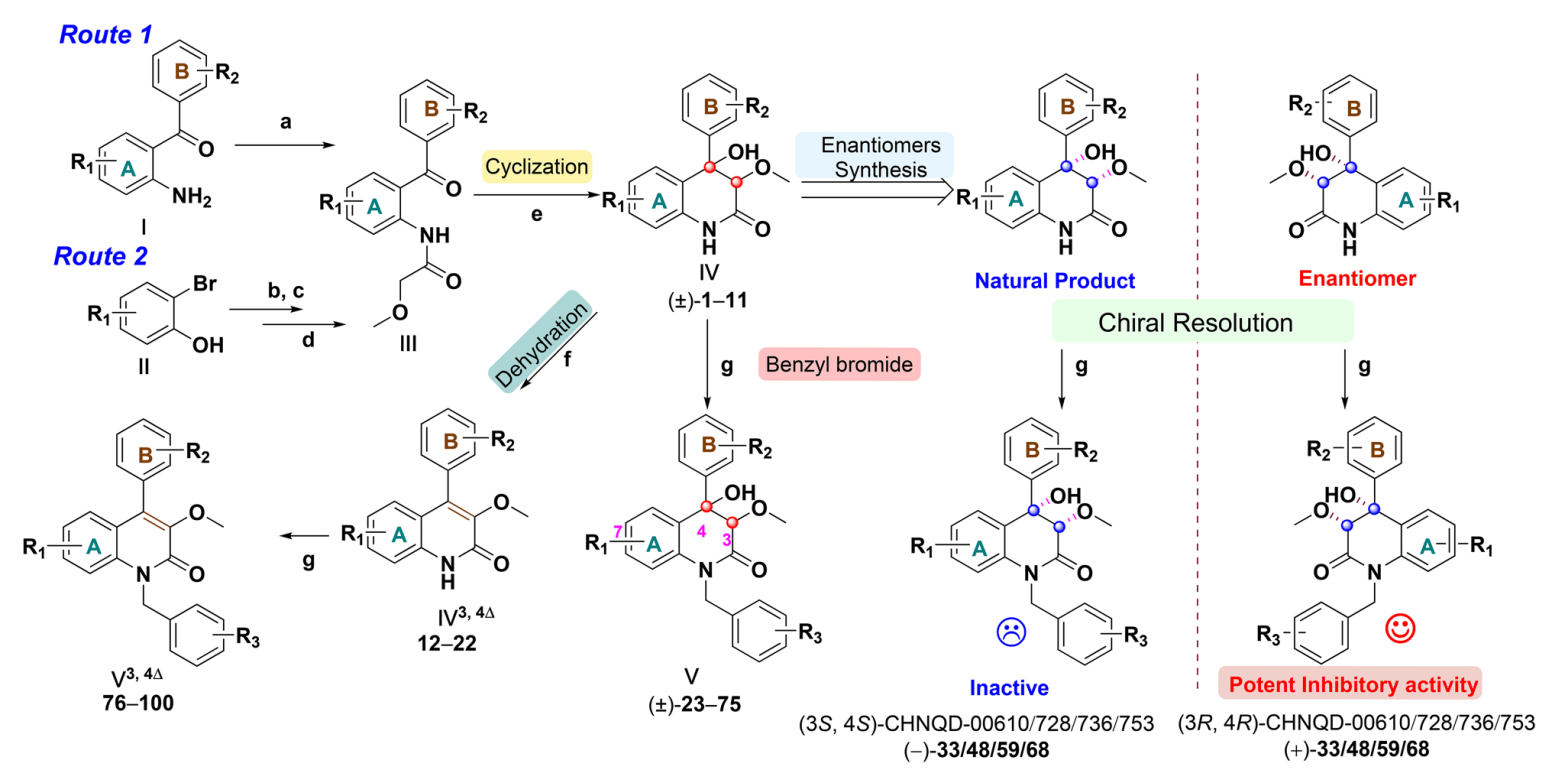
General strategy for synthesis of compounds. a. methoxyacetyl chloride, K_2_CO_3_, DCM, 30 min, rt.; b. methoxyacetyl chloride, pyridine, 80 °C; c. HMDS, THF, 75 °C; d(i). *n*-BuLi, THF, − 78 °C; d(ii). Tf_2_O; d(iii). **II-3**, CsF, MeCN, 80 °C; e. *t*-BuOK, THF, 6 h; f. pyridine, SOCl_2_, 0 °C, 1 h or KOH/EtOH, 100 °C, reflux; g. K_2_CO_3_, MeCN, 50 °C

The compounds were synthesized according to the procedure shown in Fig. 2 (Li et al. 2009; Qu et al. 2023). Two classes of compounds, racemates **IV** and dehydrated compounds IV^3,4 Δ^ were synthesized, and a series of analogues were obtained by modifying the A-ring, B-ring and amino group NH.

Herein, a general three-step synthetic route (*Route 1*) was used to synthesize the compounds. The first two steps afforded a vital intermediate **IV**, which was also the skeleton of natural 6-deoxyaflaquinolone E. Commercially acceptable *o*-aminobenzophenone or its halogen substituted regeants **I** was first treated with methoxyacetyl chloride at room temperature to form the intermediate amide **III**. After quenching with water, the raw product **III** was extracted by DCM in high purity (92–95%). The ring closure process was induced by *t*-BuOK (7 equiv) to afford intermediate **IV** and its halogen substituted derivatives in racemic form (( ±)-**1**–**11)** with high yields of 93–95%. The compound **IV** was dehydrated by thionyl chloride in pyridine at 0 °C to afford intermediate **IV**^3,4 Δ^ (**12**–**22**). The dehydration process can also be induced by KOH in EtOH refluxing at 100 °C (75%–90%). Intermediates **IV** or **IV**^3,4 Δ^ was dissolved in acetone, different substituted *α*-bromophenylmethane was added, catalysed by base catalysis (K_2_CO_3_) to obtain the first variations **V** (**23**–**49, 51**–**75**) and **V**^3,4Δ^ (**76**–**100**) in 53–65% yields.

To further investigate the effect of substituents and enrich the structure type, a second route (*Route 2*) was designed to synthesized compounds. Specifically, a three-step protocol was implemented to generate aryne precursor. Substituted 2-bromophenol **II** was transformed into TMS ether **II-1** by HMDS without further purification.

Treating TMS ether with *n*-BuLi led to halogen-metal exchange followed by a retro-Brook rearrangement to generate lithium phenolate intermediate. Then, the intermediate was trapped with triflic anhydride in situ to afford aryne precursor **II-2** (see Fig. S1) in three steps with yields of 42%–60%. Aryl imides **II-3** (see Fig. S1) could be obtained by acylation of benzamide with methoxyacetyl chloride. Aryne precursor **II-2** was inserted into the aryl imides **II-3** in the presence of CsF to afford the racemic intermediate **III**. Subsequent steps are the same as in *Route 1*. Eventually, compound **50** were synthesized.

Because of the steric hindrance effects of the benzene ring and the methoxy group, compounds **23**–**75** were all synthesized in racemic forms. By chiral resolution, single enantiomer quinolinones were fortunately purified and their absolute configurations were determined by optical rotation, CD spectra and X-ray diffraction. Optical rotation and CD spectrum of each monomer compound in different configuration showed a certain regularity, which supplied a traceable rule to the absolute configuration determination of this class of compounds.

Therefore, a total of 100 compounds (**1**–**100**) were synthesized through two routes, including 64 racemates with 11 skeleton compounds (( ±)-**1**–**11**, Fig. S1) and 53 analogues (**23**–**75**), and 36 dehydrated compounds, comprising 11 skeleton compounds (**12**–**22**, Fig. S1) and 25 analogues (**76**–**100**). Furthermore, 4 pairs of enantiomers (**33**, **48**, **59**, **68**) were obtained through chiral resolution methods.

### Inhibition of angiogenesis activity and the preliminary structure–activity relationship (SAR)

In this study, the effects of all synthesized compounds on angiogenesis were measured in zebrafish embryos expressing endothelial GFP (*Tg*(*flk1:EGFP*) (Cross et al 2003). By observing and counting the length of intersegmental blood vessels (ISVs) in zebrafish, the inhibitory rates of different compounds on blood vessel formation were obtained.

**Fig. 3:**
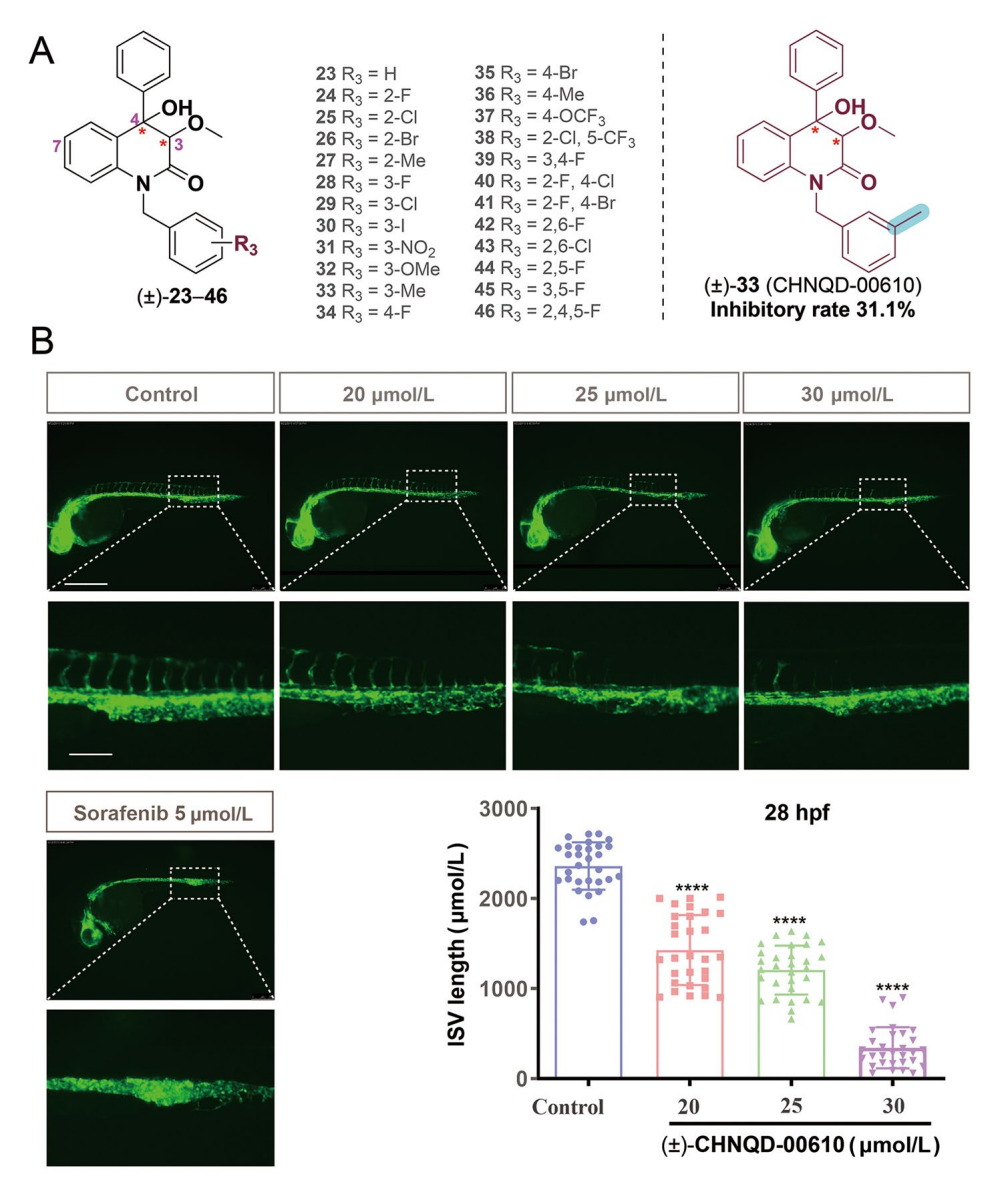
Analogues can inhibit angiogenesis in zebrafish. (A) Structure of analogues **23**–**46**. (B) Representative images and quantification of the length of ISV in *Tg(flk1:EGFP)* zebrafish at 28 h post fertilization (hpf) after ( ±)-**33** (CHNQD-00610) treatment for 24 h *(n* = 30/group). Scale bars, 500 μm (lower magnification) and 100 μm (insets). Sorafenib as positive control. Data are expressed as the mean ± SD. (****p < 0.0001)

The preliminary SAR of the 3,4-dioxygenated-4-aryl-quinolin-2(1*H*)-one alkaloids class was investigated by introducing variations on rings A and B, NH position and dehydration (Fig. 2). By comparison of anti-angiogenic activities of N-benzyl substituted 3,4-dioxygenated-4-aryl-quinolin-2(1*H*)-one alkaloids (**23**–**46**, Fig. 3A), we found that compound **33** (CHNQD-00610) with methyl substituted benzyl group showed better anti-angiogenic activity than that of other substitutions (such as fluoro, chloro, bromo, iodo, nitro, methoxyl, trifluoromethoxy groups, disubstituted or triubstituted halogens, et al.), with inhibitory rate of 31.1% at concentration of 30 μmol/L. Moreover, the anti-angiogenic activity of CHNQD-00610 with *m*-methyl substitution was found to be slightly better than compound **35** with *p*-methyl, and far superior to *o*-methyl substituted compound **25**.

At concentrations of 20, 25 and 30 μmol/L, CHNQD-00610 exhibited a dose-dependent inhibitory effect on zebrafish angiogenesis (Fig. 3B). No significant differences in overall morphology or somite number were observed between treated and control embryos. Above results suggested that CHNQD-00610 likely inhibited blood vessel formation through a more specific pathway rather than general developmental disruption.

**Fig. 4:**
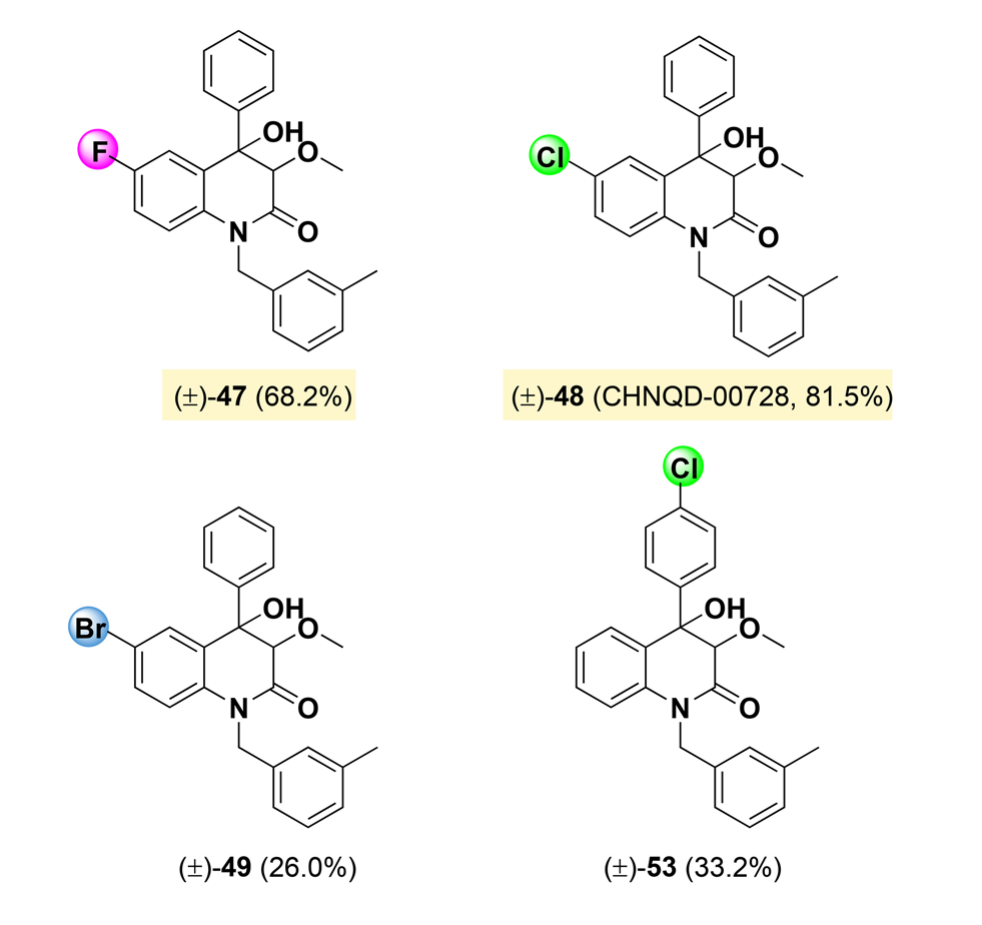
The structures of compounds of **47**–**56** and their angiogenesis inhibitory activity. ^*a*^Rate, angiogenesis inhibitory rate at concentration of 30 μmol/L. Results were the average of three independent experiments, each performed in duplicate. Standard deviations were less than ± 10%. Yellow: rate > 60%; Green: 60% > rate > 40%; Blue: rate < 40%

On the basis of compound **33**, different substituents, (such as halogen, methyl group, et al.) were introduced to ring A and B to further explore the structure–activity relationship of 3,4-dioxygenated-4-aryl-quinolin-2(1*H*)-one alkaloids. By comparing the activity of compounds **33** and **47**–**51**, it was found that halogen substitution on the A ring can enhance the activity. Compound **48**, named as CHNQD-00728, was found to be the significantly effective compound, with an inhibitory rate of 81.5% at 30 μmol/L, showing stronger activity than 7-fluoro, 7-bromo and 8-chloro, which was nearly three times higher than that of the unsubstituted compound **33**. Compared to compound **33**, halogen substitution on the B ring has little effect on activity, but for compound **48** with 7-chloro substitution, 2'-halogen substitution reduced the activity (Fig. 4).

**Fig. 5:**
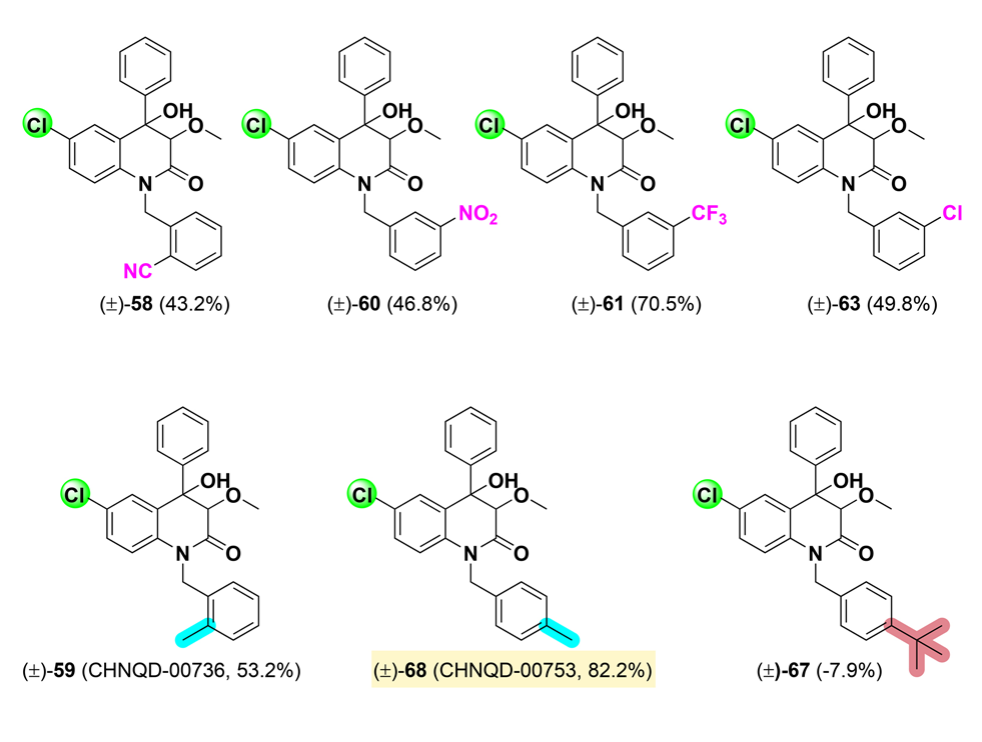
The structures of compounds of **55**–**73** and their angiogenesis inhibitory activity. ^*a*^Rate, angiogenesis inhibitory rate at concentration of 30 μmol/L. Results were the average of three independent experiments, each performed in duplicate. Standard deviations were less than ± 10%. Yellow: rate > 60%; Green: 60% > rate > 40%; Blue: rate < 40%

After two rounds of structural modification and activity screening, it was found that the substitution of fluoro, chloro, bromo at the 4' position in the B-ring has little effects for activity. Hence, on the basis, we proceeded to synthesize a series of compounds (**57**–**75**) to explore the effects of benzyl group on the activity, with the 7-chloro substitution on the A-ring and no substitution on the B-ring (Fig. 5).

**Fig. 6:**
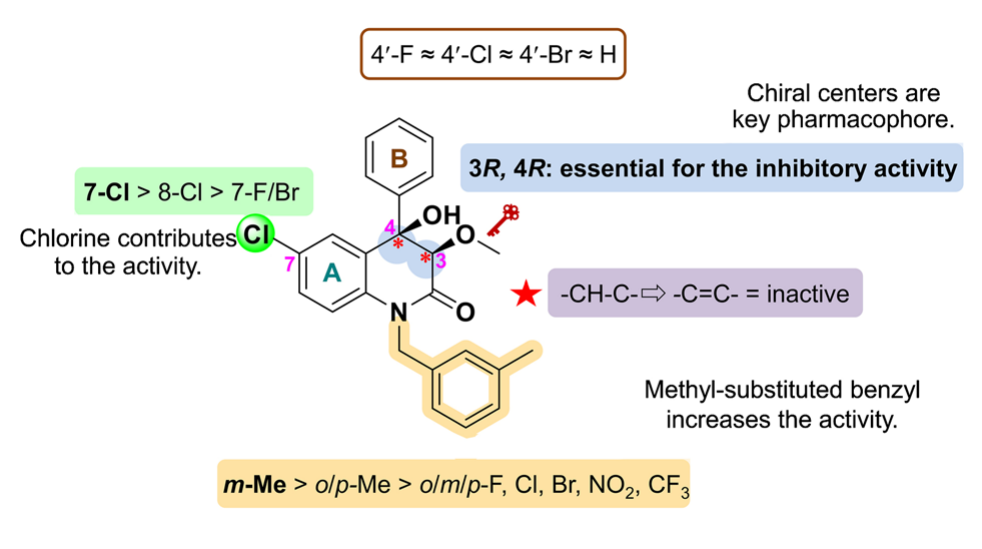
The SAR of 3,4-dioxygenated-4-aryl-quinolin-2(1*H*)-one alkaloids

To investigate the effect of the chiral centers at C-3 and 4 positions on the activity, and take the chiral resolution steps can be simplified into account, a series of dehydrated compounds (**76**–**100**) was synthesized, as shown in Fig. S2. Activity testing revealed that all dehydrated compounds were completely ineffective, indicating that the chiral centers are crucial for the inhibition of angiogenesis (Fig. 6).

**Fig. 7:**
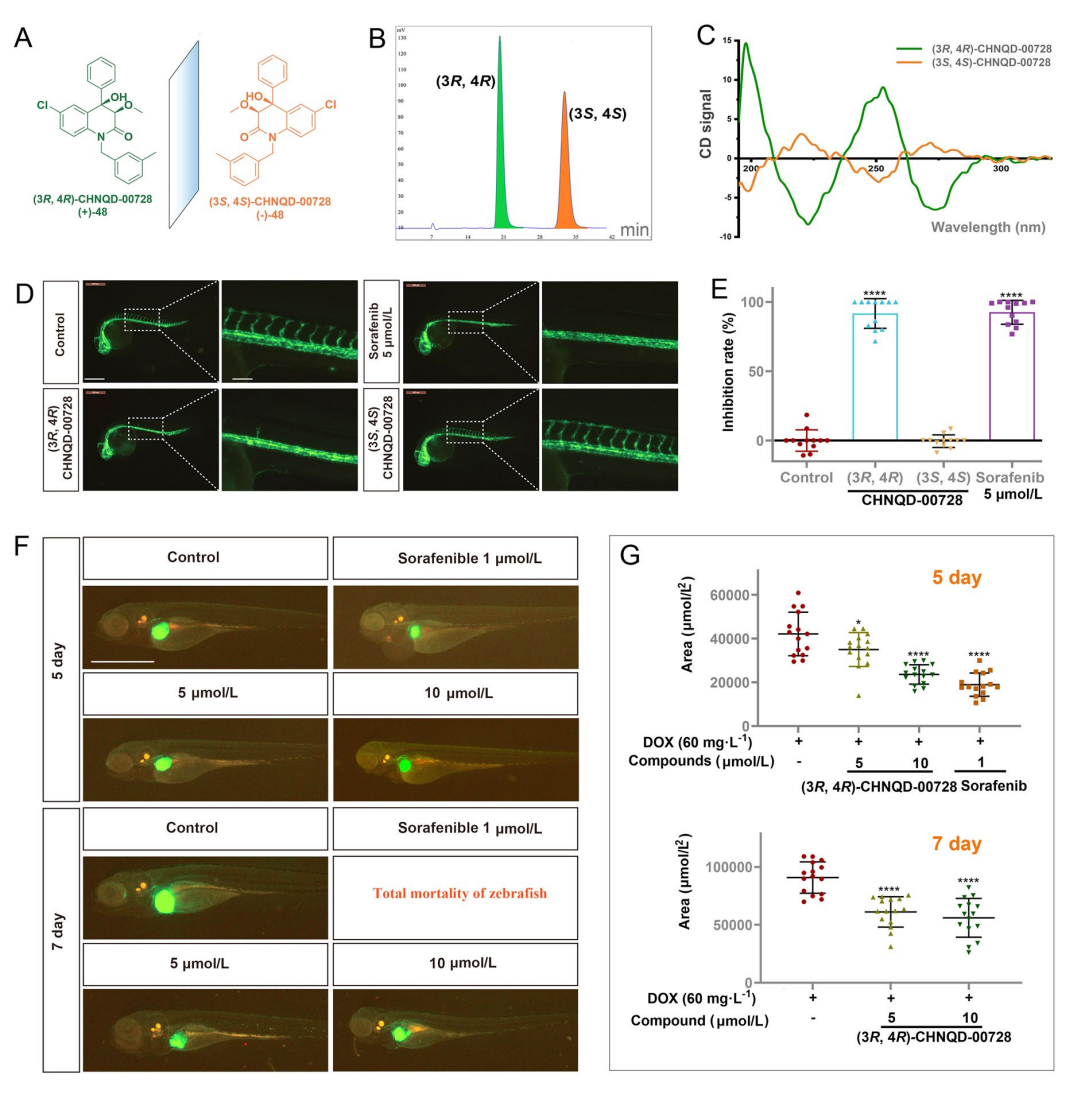
(3*R*, 4*R*)-CHNQD-00728 inhibited angiogenesis and abnormal liver enlargement in zebrafish. **A** Chemical structures of ( ±)-CHNQD-00728 (**48**). **B** Chiral-phase HPLC analysis ( ±)-CHNQD-00728. **C** The CD spectra of ( ±)-CHNQD-00728. **D** Representative images of *Tg(flk1:EGFP)* zebrafish at 28 hpf treated with (3*R*, 4*R*)-CHNQD-00728, (3*S*, 4*S*)-CHNQD-00728 at concentration of 30 µmol/L, or sorafenib for 24 h. Scale bars, 500 μm (lower magnification) and 120 μm (insets). Sorafenib as positive control. **E** Inhibition rate based on ISV length quantification (n = 15/group). **F** (3*R*, 4*R*)-CHNQD-00728 inhibited abnormal liver enlargement in *TO* (*kras*^*G12V*^) zebrafish (*n* = 20/group). Scale bars, 1 mm. **G** The size of live zebrafish was measured at 5 (upper panel) and 7 dpf (lower panel). Data are expressed as the mean ± SD. *p < 0.05; ****p < 0.0001

Hence, the racemates were separated by chiral resolution (Fig. 7A–C, Fig. S3), to further study the differences in biological activity between the enantiomers. Surprisingly, compounds with *S*,* S* configuration were inactive, and only *R*,* R*-configured compounds inhibited angiogenesis. Compound ( +)-**48**, named as (3*R*, 4*R*)*-*CHNQD-00728, at concentration of 30 μmol/L can significantly inhibit angiogenesis in zebrafish embryos, with an inhibitory rate of over 90% (Fig. 7D, E). The same results were also found in compounds CHNQD-00610 (**33**), CHNQD-00736 (**59**) and CHNQD-00753 (**68**) (Fig. S4A, B). The finding that only compounds with *R, R*-configuration exhibited activity strongly suggested a critical role for the configurational site in the activity of these racemates.

Building on the discovery of the *R*-configuration’s critical role, further studies focused on compound (3*R*, 4*R*)-CHNQD-00728 *Tg*(*flk1*:*EGFP*) embryos were treated with varying concentrations of (3*R*, 4*R*)-CHNQD-00728. These experiments revealed a dose-dependent inhibition of angiogenesis (Fig. S4C, D). Notably, at concentration of 40 μmol/L, the compound completely blocked angiogenesis, mirroring the effect of sorafenib, a known anti-angiogenic drug. However, unlike sorafenib, which exhibited cardiotoxicity in zebrafish embryos, (3*R*, 4*R*)-CHNQD-00728 did not display such adverse effects at this concentration. These findings highlight (3*R*, 4*R*)-CHNQD-00728 as a potent vasoactive agent with superior selectivity for inhibiting angiogenesis compared to (3*S*, 4*S*)-CHNQD-00728, which was inactive.

### Antitumor efficacy in *TO* (*kras*^*G12V*^) zebrafish

Given that tumor growth depends on angiogenesis, targeting blood vessel formation has emerged as a promising strategy for developing anti-cancer drugs (Cao et al. 2023; Carmeliet and Jain 2011; Ferrara and Kerbel 2005; Folkman 1971, 2007; Hanahan and Folkman 1996; Prokopiou et al. 2013; Weis and Cheresh 2011). Compound CHNQD-00728 demonstrated anti-angiogenic properties, prompting the investigation of its potential to suppress tumor growth. To explore this possibility, we employed the *TO(kras*^*G12V*^*)* transgenic zebrafish model. In this model, doxycycline hydrochloride (DOX) activated liver-specific expression of the oncogenic kras gene, ultimately leading to abnormal liver enlargement and progression to liver cancer (Nguyen et al. 2012). Therefore, we utilized the *TO(kras*^*G12V*^*)* model to evaluate the effect of (3*R*, 4*R*)-CHNQD-00728 on hepatocellular carcinoma (HCC) growth in vivo. The results revealed that it inhibited DOX-induced liver enlargement at concentrations of 5 and 10 µmol/L (Fig. 7F, G) at both 5 and 7 days post-fertilization (dpf). Most notably, all zebrafish in the sorafenib group died due to toxic effects, while all survived in the (3*R*, 4*R*)-CHNQD-00728 group, which is also consistent with the non-cytotoxic results in the cells (Hela, HCT116 and EA. hy 926). These findings suggested that (3*R*, 4*R*)-CHNQD-00728 holds promise as a therapeutic agent for liver cancer.

### Inhibition of VEGFR2 and p-ERK expression, and regulation of angiogenesis-related genes

**Fig. 8:**
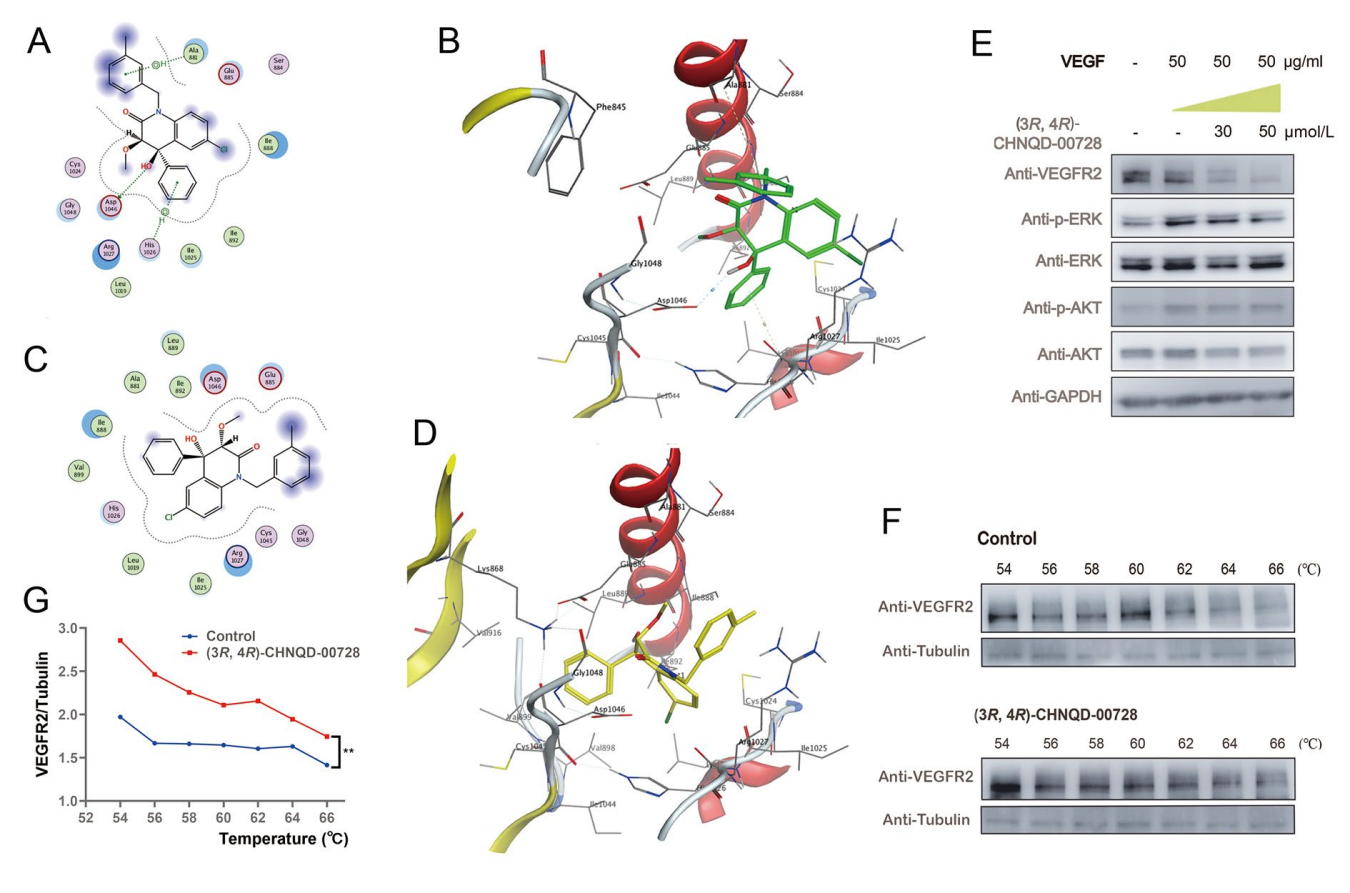
(3*R*, 4*R*)-CHNQD-00728 bound to VEGFR2 and inhibited both VEGFR2 protein levels and VEGF-induced p-ERK expression. **A**, **B** 2D and 3D molecular docking binding interactions of (3*R*, 4*R*)-CHNQD-00728 with VEGFR2 (PDB: 5EW3). **C**, **D** 2D and 3D molecular docking binding interactions of (3*S*, 4*S*)-CHNQD-00728 with VEGFR2. Molecular docking was performed by using MOE software. **E** Western blots of VEGFR2, pAKT and AKT, pERK and ERK induced by 50 μg/mL VEGF in HUVECs with (3*R*, 4*R*)-CHNQD-00728 treatment at concentrations of 0, 30 and 50 µmol/L. **F**, **G** Cellular thermal shift assay of the binding of (3*R*, 4*R*)-CHNQD-00728 to VEGFR2

To investigate the anti-angiogenic mechanism of (3*R*, 4*R*)-CHNQD-00728, molecular docking was conducted. The results revealed that (3*R*, 4*R*)-CHNQD-00728 was well-fitted in the active site of VEGFR2 and occupied the hydrophobic pocket with ring A and B, showed a high degree of selectivity and potency toward VEGFR2. Moreover, the binding complex also interacted by one conventional hydrogen bond and two π-H interactions with three amino acids (ASP1046, ALA881, and HIS1026) as clarified in Fig. 8A and B. Conversely, compound (3*S*, 4*S*)-CHNQD-00728 showed only weak hydrophobic interactions with protein VEGFR2 (Fig. 8C, D). Additionally, docking with other angiogenesis-related molecules (EGFR, PDGFR, and ERK) revealed no significant binding affinity. Since VEGFR2 signaling is critical for tumor blood vessel growth, we investigated its role further. Treatment with (3*R*, 4*R*)-CHNQD-00728, VEGFR2 protein levels in multiple cell lines were reduced (Fig. 8E, Fig. S5A–C).

As anticipated, downstream signaling molecules of the VEGFR2 pathway, p-ERK, were also markedly decreased after VEGF treatment (Fig. 8E, Fig. S5A–C). A slight decrease in p-AKT was also observed. In contrast, (3*R*, 4*R*)-CHNQD-00728 cannot inhibit EGF-induced p-ERK or p-AKT protein levels (Fig. S5F). This finding highlighted the specificity of (3*R*, 4*R*)-CHNQD-00728 for the VEGFR2 pathway. Interestingly, it did not affect VEGFR2 mRNA levels, suggesting it targets protein stability.

Cellular thermal shift assays demonstrated that it increased VEGFR2 thermal stability (Fig. 8F), supporting that there is a direct interaction between VEGFR2 and (3*R*, 4*R*)-CHNQD-00728. Additionally, ELISA experiments demonstrated that it had no effect on VEGF secretion in 786-O and RCC4 cells (Fig. S6B). We also investigated the effect of (3*R*, 4*R*)-CHNQD-00728 on hypoxia-inducible factor 1α (HIF-1*α*), a key upstream signaling pathway in angiogenesis (Masoud and Li 2015; Palazon et al. 2017; Weidemann and Johnson 2008; Zhao et al. 2024), and found (3*R*, 4*R*)-CHNQD-00728 and its analogue (3*R*, 4*R*)-CHNQD-00610 did not affect HIF-1*α* protein levels or its transcriptional activity (Fig. S5D, E). To gain a deeper understanding of the mechanisms by which (3*R*, 4*R*)-CHNQD-00728 inhibits angiogenesis, we investigated the expression of several angiogenesis-related genes in various cell types. Our findings revealed that both (3*R*, 4*R*)-CHNQD-00728 and (3*R*, 4*R*)-CHNQD-00610 can suppress the mRNA expression of pro-angiogenic genes, including PDGF-BB, TGF-β, EPO, HMOX1, and FMNL3 (Fig. S6A, S7).

### Effects of (3*R*, 4*R*)-CHNQD-00728 on angiogenesis in vitro

**Fig. 9:**
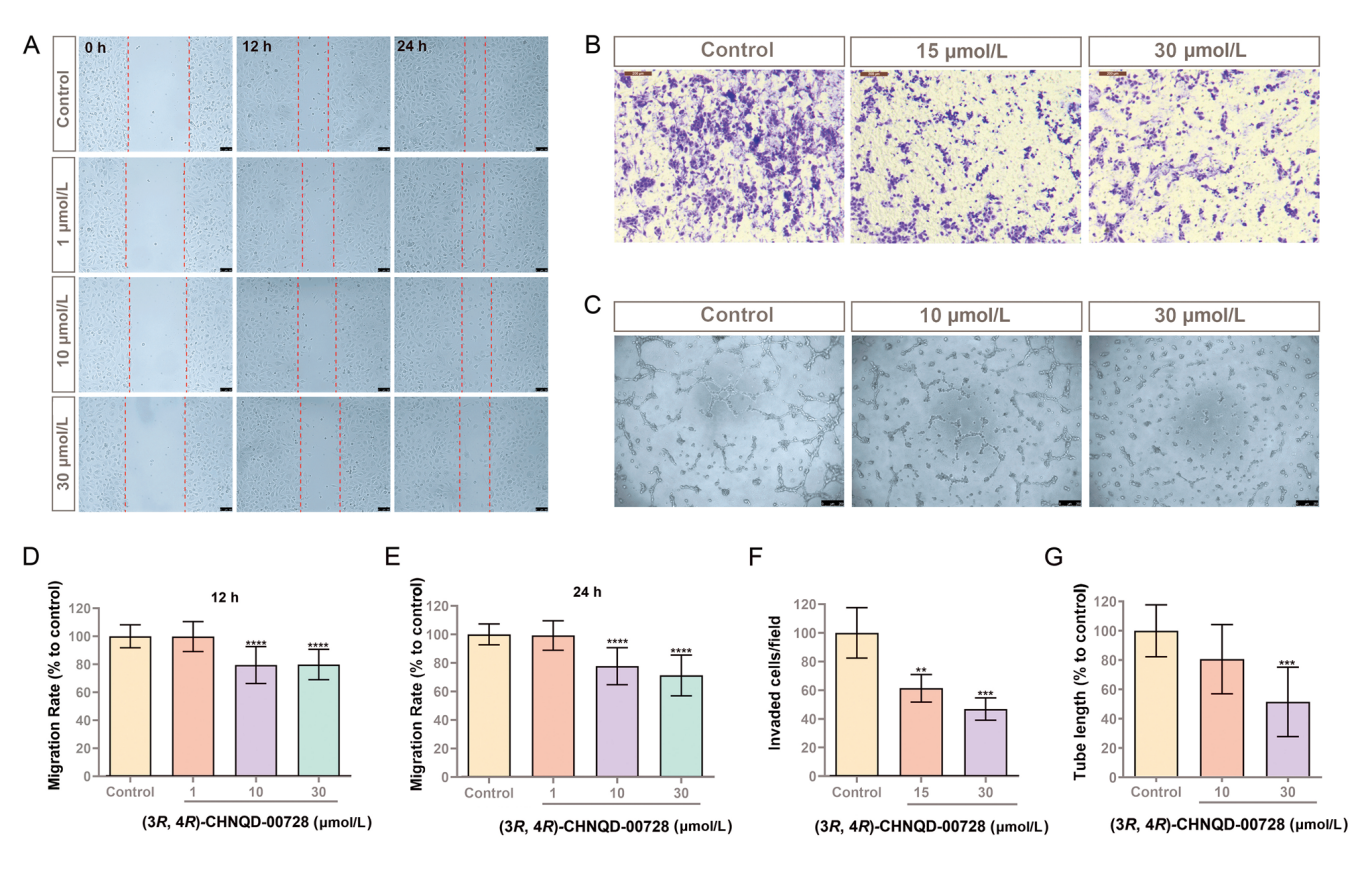
(3*R*, 4*R*)-CHNQD-00728 inhibited endothelial migration. **A**, **D**, **E** Scratch migration and quantification in endothelial cells with 0, 1, 10 and 30 μmol/L of (3*R*, 4*R*)-CHNQD-00728 treatment for 12 and 24 h. Scale bar, 100 μm. **B**, **F** Transwell migtation and quantifiction of migration cell number in HUVECs with (3*R*, 4*R*)-CHNQD-00728 treatment at concentrations of 0, 15 and 30 μmol/L. Scale bar, 200 μm. **C**, **G** Tube formation and quantification of tube length in HUVECs with (3*R*, 4*R*)-CHNQD-00728 treatment at concentrations of 0, 10 and 30 μmol/L. Scale bar, 250 μm. ANOVA in all analyses. **p < 0.01; ***p < 0.001; ****p < 0.0001

To further assess the functional impact of (3*R*, 4*R*)-CHNQD-00728 on angiogenesis, we employed wound-healing and transwell migration assays. These assays revealed that treatment with (3*R*, 4*R*)-CHNQD-00728 significantly inhibited endothelial cell migration in a dose-dependent manner (Fig. 9A, B and D–F). Similarly, capillary tube formation assays demonstrated that the compound at concentration of 30 μmol/L significantly reduced both total branch length and the number of new tubes formed (Fig. 9C and G). Notably, (3*R*, 4*R*)-CHNQD-00610 also exhibited moderate inhibitory effects on capillary tube formation (Fig. S8). These findings collectively suggested that (3*R*, 4*R*)-CHNQD-00728 has the potential to disrupt both endothelial cell migration and tube formation, key processes essential for angiogenesis.

## Discussion

Currently, cancer treatment primarily relies on cytotoxic drugs, which directly kill or inhibit the growth and proliferation of tumor cells. However, these cytotoxic chemotherapeutic agents possess non-selective cytotoxicity, poor tissue selectivity, and severe systemic adverse reactions. These characteristics lead to poor patient tolerance and prognosis. Moreover, tumor cells are not completely eradicated, and drug resistance is inevitably increased. As an emerging therapeutic approach, anti-angiogenic therapies combat cancer by inhibiting the formation of new blood vessels and limiting distant tumor invasion and metastasis (Liu et al. 2023a, b). Despite the growing list of FDA-approved drugs, the clinical benefits of anti-angiogenic therapies have not been long-lasting, and some side effects, such as acquired drug resistance and tumor recurrence, have been observed in antiangiogenic therapy. This may also be due to the fact that the current pipeline of anti-tumor drugs involves chemical modifications of existing antitumor agents, or on drug design targeting known targets and mechanisms of action. There is an urgent need to develop innovative anti-angiogenic drugs of new chemical class, different mechanisms of action or novel targets.

**Fig. 10:**
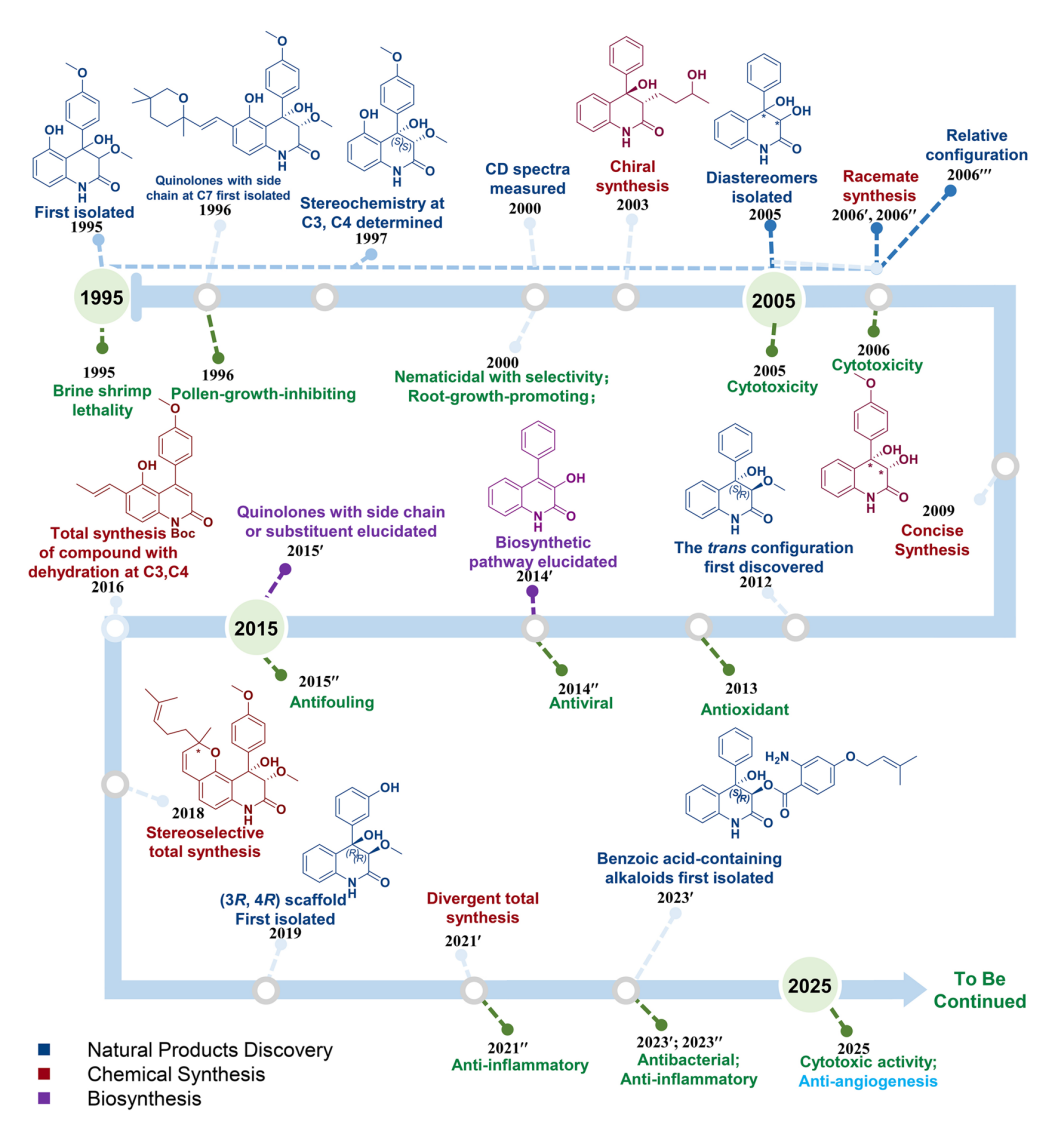
The timeline of 3,4-dioxygenated-4-aryl-quinolin-2(1*H*)-one alkaloids development

NPs serve as an important source pool for innovative drug development. From 1981 to 2019, among the nearly 1400 small molecules approved, about 63.1% were related to NPs. MNPs, due to their unique habitats, have the characteristics of novel structures and significant activities. Currently, there are 17 marine drugs have been approved (Zhang et al. 2024). During the last 30 years, a family of 3,4-dioxygenated-4-aryl-quinolin-2(1*H*)-one alkaloids (Fig. 10) has been isolated from terrestrial and marine fungi, with or without a side chain at C-7 position (Chen et al. 2014; Dai et al. 2021; El-Kashef et al. 2019; He et al 2005; Hu et al. 2023; Kimura et al 1996; Kusano et al. 2000; Mou et al. 2017; Nakaya 1995; Neff et al. 2012; Schmeda-Hirschmann et al. 2005; Scherlach and Hertweck 2006; Simonetti et al. 2016a; Uchida et al. 2006a, b; Wubshet, et al. 2013). In recent years, the total synthesis (Guo et al. 2023a, 2022, 2023b; Jia et al. 2021; Li et al. 2009; Schwan et al. 2018, 2020; Simonetti et al. 2016b; Ueki et al. 2003; Vece et al 2018; Wang et al. 2003; Zou et al. 2015) and biosynthesis (Ishikawa et al. 2014; Uchida et al. 2006a, b) of these NPs—characterized by their novel and complex structures and diverse activities, such as cytotoxic, antiviral, antioxidant, anti-inflammatory, antibacterial, brine shrimp lethality, antifouling, nematicidal, root-growth-promoting and pollen growth inhibiting activity (Kang et al. 2025; Qu et al. 2023; Simonetti et al. 2016a, b)—have been continuously investigated (Table S1, Fig. S9). Among these NPs, we found that the chiral centers at C3 and C4 are almost always of the 3*S*, 4*S* configuration, with very few 3*R*, 4*R* NPs, especially in those scaffolds without side chains (An et al. 2013a, 2013b; Hayashi et al. 1997; Shao et al. 2015).

In this study, we obtained 3*S*, 4*S* and its enantiomeric 3*R*, 4*R* compounds without side chains by synthesis and chiral resolution, and found that the enantiomers 3*R*, 4*R* possessed potent angiogenesis inhibitory activity in vivo and in vitro, whereas 3*S*, 4*S* was completely inactive. To the best of our knowledge, this is the first report on the enantiomer-activity relationship for this class of compounds, as well as the first report on their angiogenesis inhibitory activity. The compounds in this study have demonstrated significant anti-angiogenic activity in vitro and in vivo. More importantly, they are non-cytotoxic, coupled with a unique chiral configurations-activity relationship, which may make it different mechanism of action of the currently marketed anti-angiogenic inhibitors. This further indicates that this class of compounds has the potential to be developed into a novel type of safe and effective anti-angiogenic inhibitors.

Moreover, our research findings prompt us to consider whether the scarcity of 3*R*, 4*R* configured NPs is due to chiral switching, or they are extremely rare in nature and have not been detected, or perhaps these molecules and their activities are not essential for the survival of marine organisms. This highlights the significance of chirality in NPs and the mining of NPs dark matter from another perspective. It reminds us that we should not only focus on chiral natural products themselves, but also on the enantiomers that are not identical or completely different from the configuration of the NPs (often considered as “waste” or “ineffective” enantiomers). Perhaps these “waste” compounds may bring us a bright discovery, achieving the goal of “turning waste into treasure.”

Nevertheless, although we have currently discovered the role of 3*R*, 4*R*-configured compounds in inhibiting angiogenesis, the functions of 3*R*, 4*S* and 3*S*, 4*R* compounds remain unclear. Thus, continuing to synthesize and explore the functions of 3*R*, 4*S* and 3*S*, 4*R* compounds, as well as investigating the relationship between marine chemical defense mechanisms and pharmacological activities, will be crucial for systematically studying the relationship between configuration and activity of this class of compounds and for drug development.

## Conclusion

Herein, we have described the importance of chirality and the fundamental position of chiral natural products in drug development. By constructing the compound library of 3,4-dioxygenated-4-aryl-quinolin-2(1*H*)-one alkaloids with racemates and enantiomers, and through multiple extensive activity screening, it was found that the enantiomer (3*R*, 4*R*)*-*CHNQD-00728 possessed potent angiogenesis inhibitory activity in *Tg(flk1:EGFP)* zebrafish, together with favorable effects on angiogenesis in vitro. Moreover, evidence of antitumor activity in vivo was demonstrated in *TO* (*kras*^*G12V*^) zebrafish. While the corresponding compound (3*S*, 4*S*)-CHNQD-00728 had no activity completely, which is the conformation possessed by the NP. The result highlighted the importance of NPs from another perspective and elucidated the potential of NPs enantiomers in expanding and inspiring drug development resources. It’s essential to unlock the latent functionalities of the “dark matter” of NP enantiomers. Moreover, it will provide theoretical guidance for the fields of organic synthesis, medicinal chemistry, and innovative drugs research. It is anticipated that enantiomeric NPs will achieve their unique potential in drug development.

## Statistical analysis

GraphPad Prism 9.0 was used for data analysis. All the results were represented as mean ± SD. The differences among groups were determined using one-way analysis of variance (ANOVA); *p < 0.05, **p < 0.01, ***p < 0.001, and ****p < 0.0001 were considered statistically significant. All the experiments were performed at least in triplicates for every condition.

## Supplementary Information

Below is the link to the electronic supplementary material.

Supplementary file1 (DOCX 47372 KB)

## Data Availability

Additional data related to this paper may be requested from the authors.

## Abbreviations

NPs: Natural products
MNPs: Marine natural products
DCM: Dichloromethane
K_2_CO_3_: Potassium carbonate
EtOAc: Ethyl acetate
THF: Tetrahydrofuran
*t*-BuOK: Potassium *tert*-butoxide
NH_4_Cl: Ammonium chloride
SOCl_2_: Thionyl chloride
*n*-BuLi: *n*-butyllithium
MeCN: Acetonitrile
HMDS: Hexamethyldisilazane
TMS: Tetramethylsilane
Tf_2_O: Trifluoromethanesulfonic anhydride
Na_2_SO_4_: Sodium sulfate
Na_2_CO_3_: Sodium carbonate
CsF: Cesium fluoride
SAR: Structure–activity relationship
VEGFR2: Vascular endothelial growth factor receptor 2
ISVs: Intersegmental blood vessels
hpf: Hour post fertilization
