# Supplementary material for: Trash or treasure? Unlocking dark matter of enantiomeric natural products in innovative drugs discovery for potent angiogenesis inhibitors
Source: Mar Life Sci Technol. 2025 Jul 10;8(1):164–79. doi: 10.1007/s42995-025-00307-8 (PMC12953838; doi:10.1007/s42995-025-00307-8)
Supplement: Supplementary file 1 — Supplementary file1 (DOCX 47372 KB) [file 42995_2025_307_MOESM1_ESM.docx]

**Supporting Information**

**Trash or treasure? Unlocking dark matter of enantiomeric natural products in innovative drugs discovery for potent angiogenesis inhibitors**

Yan-Wei Wu,^1^ Xiao-Feng Mou,^1^ Zhong-Yuan Chen,^1^ Xiao-Jia Xue,^1^ Wen-Hui Wang,^1^ Jin-Zhou Guo,^1^ Bo-Qi Zhang,^1^ Ting-Ting Xue,^1^ Qun Zhang,^1^ Mei-Yan Wei,^1^ Yu-Cheng Gu,^2^ Gulab Said^1,3^, Chang-Yun Wang,^1^ Ling Lu,^1^ Chang-Lun Shao^1, 4^

1 Key Laboratory of Marine Drugs, The Ministry of Education of China, School of Medicine and Pharmacy, Ocean University of China, Qingdao 266003, People’s Republic of China

2 Syngenta Jealott’s Hill International Research Centre, Bracknell, Berkshire, RG42 6EY, UK

3 Department of Chemistry, Women University Swabi, Swabi 23430, Pakistan

4 Key Laboratory of Tropical Medicinal Resource Chemistry of Ministry of Education, College of Chemistry and Chemical Engineering, Hainan Normal University, Haikou 571158, People’s Republic of China

* Correspondence: shaochanglun@163.com (Chang-Lun Shao); linglu@ouc.edu.cn (Ling Lu); changyun@ouc.edu.cn (Chang-Yun Wang)

Yan-Wei Wu, Xiao-Feng Mou and Zhong-Yuan Chen contributed equally to this work.

**Table of Contents**

[Structures of skeleton compounds and dehydrated compounds. S3](#_Toc190437756)

[CD spectra of enantiomeric compounds S5](#_Toc190437757)

[Inhibitory effect of enantiomeric compounds. S6](#_Toc190437758)

[Inhibition of VEGFR2 and p-ERK expression, and regulation of angiogenesis-related genes S7](#_Toc190437759)

[Effects of (3*R*, 4*R*)-CHNQD-00610 on angiogenesis *in vitro*. S9](#_Toc190437760)

[Experimental Section S10](#_Toc190437761)

[Chemistry S10](#_Toc190437762)

[General experimental procedures S10](#_Toc190437763)

[Bioactivity S10](#_Toc190437764)

[Cell lines S10](#_Toc190437765)

[Animals S11](#_Toc190437766)

[Plasmids S11](#_Toc190437767)

[*In vivo* anti-angiogenesis assay in zebrafish embryos S11](#_Toc190437768)

[Western blot analysis S11](#_Toc190437769)

[Wound healing assay S12](#_Toc190437770)

[Transwell invasion assay S12](#_Toc190437771)

[Tube formation assay S12](#_Toc190437772)

[Real-Time quantitative PCR (qPCR) analysis S12](#_Toc190437773)

[Enzyme-linked immunosorbent assay (ELISA) S13](#_Toc190437774)

[*In vivo* anticancer assay in zebrafish embryos S13](#_Toc190437775)

[Characterization data S14](#_Toc190437776)

[Spectra of compounds S35](#_Toc190437777)

# **Structures of skeleton compounds and dehydrated** **compounds.**

**Figure S1**. Structures of skeleton compounds ((±)-1−11), dehydrated skeletal compounds (12−22) and intermediates (Ⅱ-1−Ⅱ-3) in *route 2*.

**Figure S2**. Structures of dehydrated compounds (76−100).

# CD spectra of e**nantiomeric compounds**


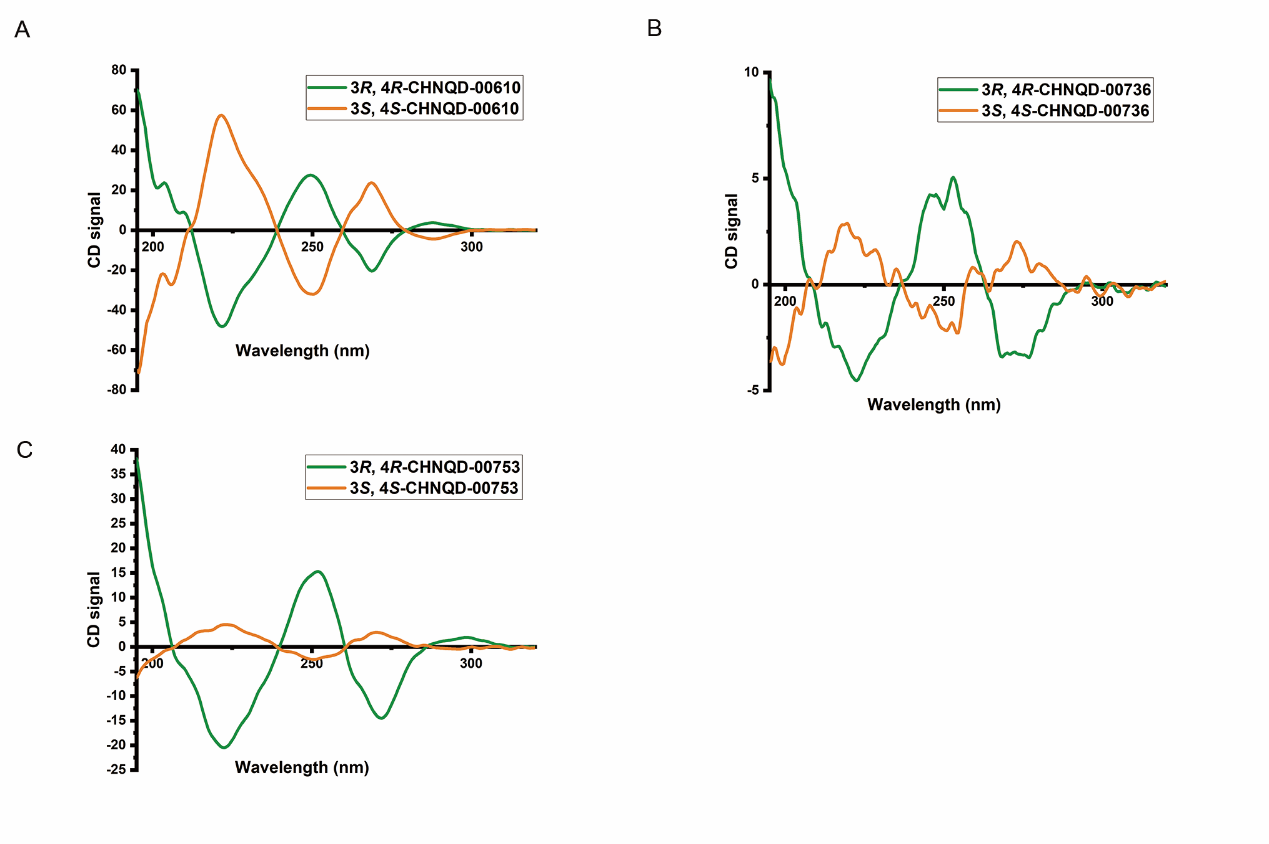


Figure S3. CD Spectra of Enantiomeric Compounds, (A) CHNQD-00610, (B) CHNQD-00736 and (C) CHNQD-00753.

# **Inhibitory effect of enantiomeric compounds.**


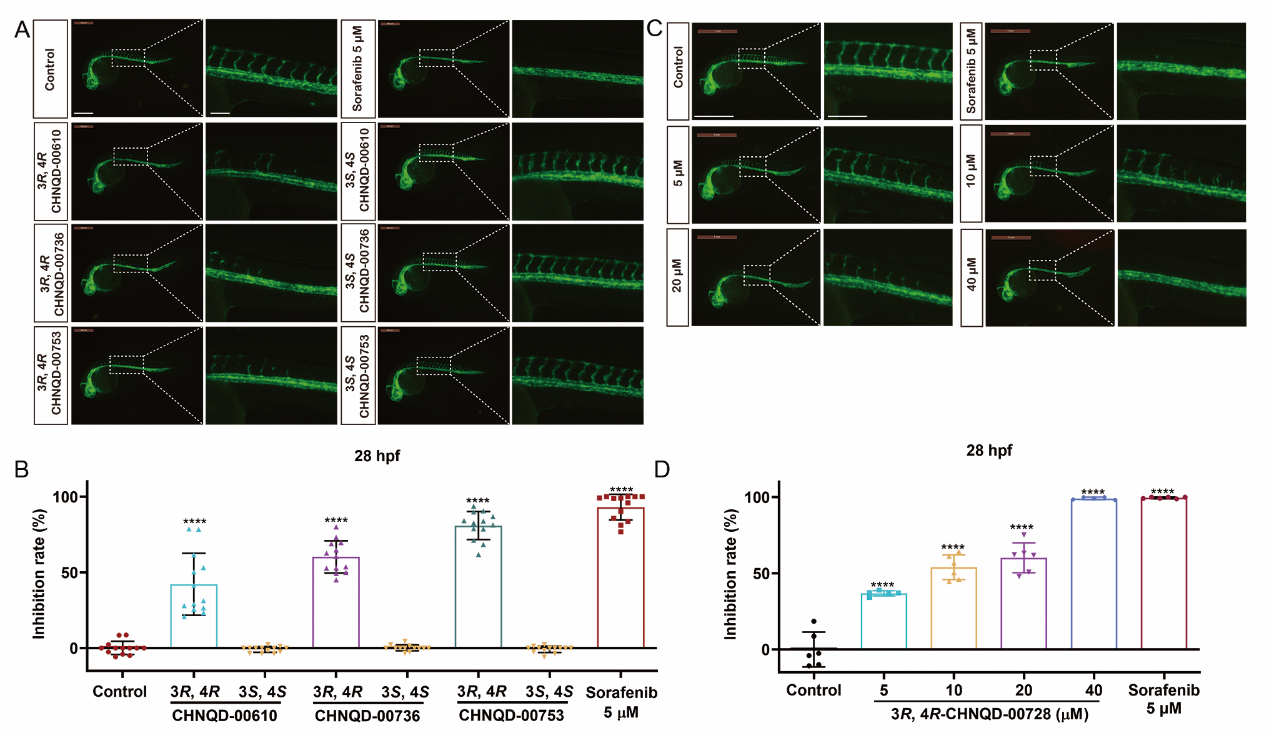


Figure S4. Analogues inhibited angiogenesis in zebrafish. (A) Representative images of *Tg*(*flk1:EGFP*) zebrafish at 28 hpf treated with enantiomeric CHNQD-00610, CHNQD-00736, CHNQD-00753 at concentration of 30 µM and sorafenib for 24 h (n = 30/group). Scale bars, 500 μm (lower magnification) and 120 μm (insets). Sorafenib as positive control. (B) Quantification of blood vessel inhibition based on ISV length. (C) Representative images of *Tg*(*flk1:EGFP*) zebrafish treated with 3*R*, 4*R*-CHNQD-00728 at concentrations of 5, 10, 20 and 40 µM and sorafenib for 24 h. Scale bars, 1 mm (lower magnification) and 250 μm (insets). Sorafenib as positive control. (D) Quantification of blood vessel inhibition based on ISV length.

# **Inhibition of VEGFR2 and p-ERK expression, and regulation of angiogenesis-related genes**


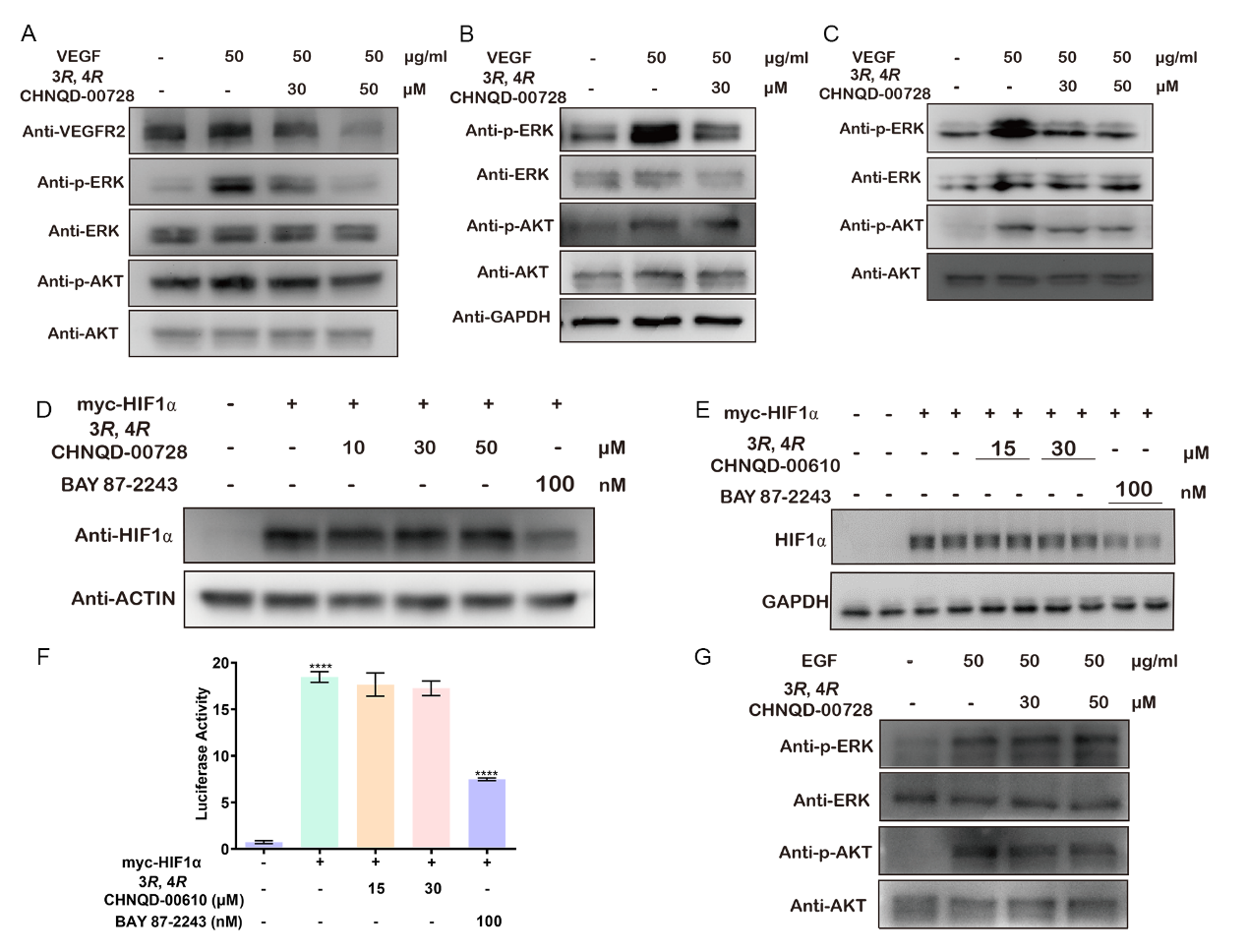


**Figure S5.** 3*R*, 4*R*-CHNQD-00728 inhibited VEGFR2 protein levels and VEGF-induced p-ERK expression. (A−C) Western blot analysis of VEGFR2, pERK and pAKT protein levels. (A) HEK293T/17 cells transfected with pcDNA3.1-VEGFR2 vector, (B) EA. hy926 cells, and (C) HCT116 cells were treated with 0, 30, or 50 μM 3*R*, 4*R*-CHNQD-00728 for 24 h, followed by stimulation with 50 ng/mL VEGF for 15 min. (D) Western blot analysis of HIF1α protein levels in HEK293T/17 cells. Cells were treated with 0, 10, 30 and 50 µM 3*R*, 4*R*-CHNQD-00728 or 100 nM BAY 87-2243 for 24 h. Prior to compound addition, cells were transfected with either empty vector or expression vector encoding myc-HIF1*α*. (E) Western blot analysis of HIF1α protein levels in HEK293T/17 cells. Cells were treated with 0, 15 and 30 µM 3*R*, 4*R*-CHNQD-00610 or 100 nM BAY 87-2243 for 24 h. Prior to compound addition, cells were transfected with either empty vector or expression vector encoding myc-HIF1α. (F) HIF transactivity assay in HEK293T/17 cells. Cells were co-transfected with p2.1 or pSV-Renilla and the indicated expression vector, followed by treatment with 3*R*, 4*R*-CHNQD-00610 for 24 h. Luciferase activity was normalized to Renilla activity. Data are represented as the mean ± SD (n = 3). *****p* < 0.0001. (G) Western blot analysis of pERK, ERK, pAKT, and AKT protein levels in HUVECs treated with 0, 30, or 50 μM 3*R*, 4*R*-CHNQD-00728 for 24 h, followed by stimulation with 50 ng/mL EGF for 15 min.


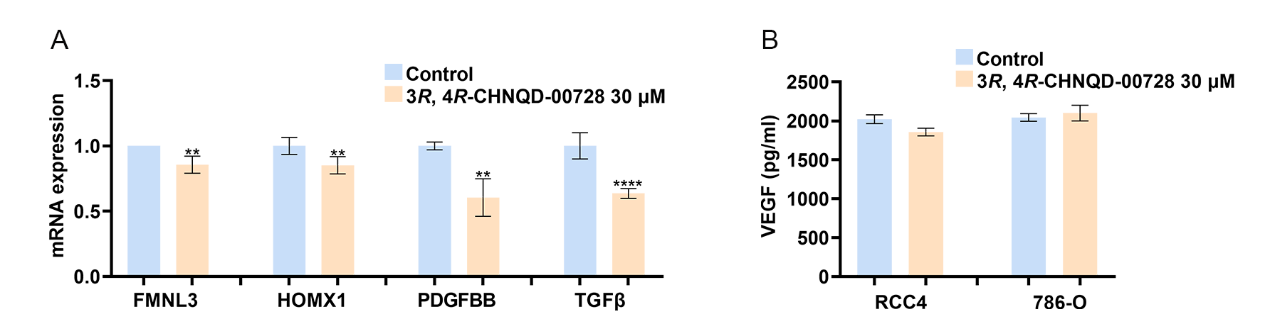


Figure S6. Effects of 3*R*, 4*R*-CHNQD-00728 modulated expression of angiogenesis-related genes. (A) qRT-PCR analysis of mRNA expression levels of FMNL3, HOMX1, PDGFBB, and TGFβ in HUVECs treated with 3*R*, 4*R* -CHNQD-00728 for 24 h. (B) ELISA analysis of VEGF secretion in 786-O and RCC4 cells treated with 3*R*, 4*R*-CHNQD-00728 for 24 h. Data were represented as means ± SD (n = 3). ***p* < 0.01; *****p* < 0.0001.


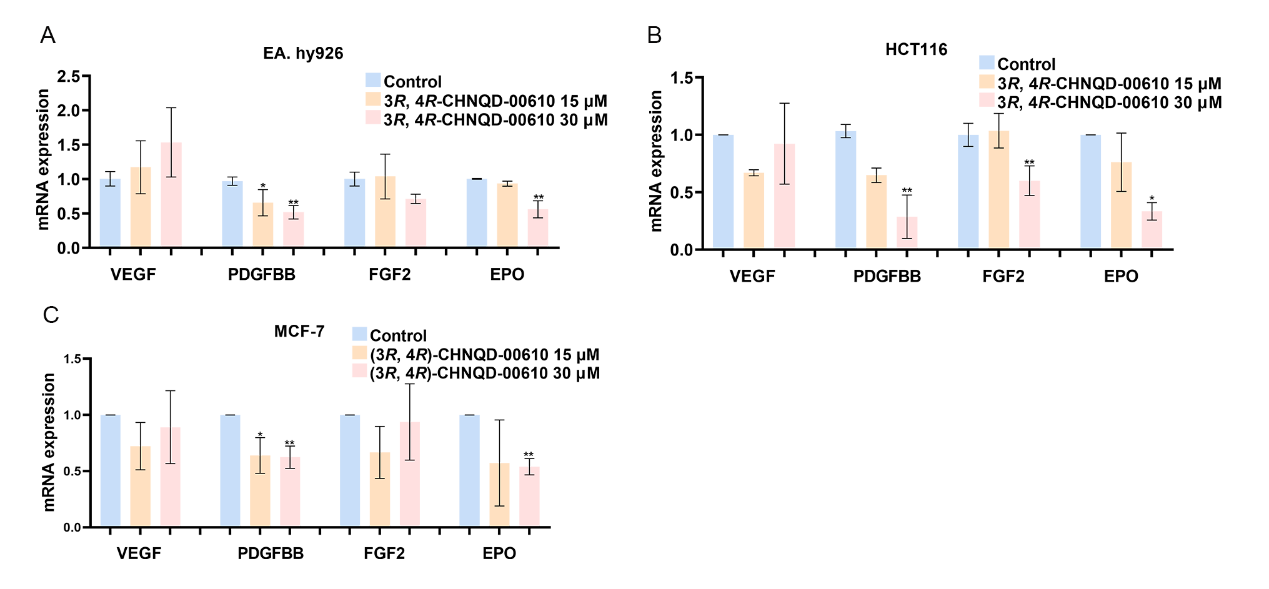


Figure S7. Effects of 3*R*, 4*R*-CHNQD-00610 on mRNA expression of VEGF, PDGFBB, FGF2 and EPO in (A) EA. hy926 cells, (B) HCT116 cells and (C) MCF-7 cells. Results were statistically analyzed after treatment with 15 μM and 30 μM 3*R*, 4*R*-CHNQD-00610 for 12 h. Data were represented as means ± SD (n = 3). **p* < 0.1; ***p* < 0.01.

# **Effects of (3*R*, 4*R*)-CHNQD-00610 on angiogenesis *in vitro*.**


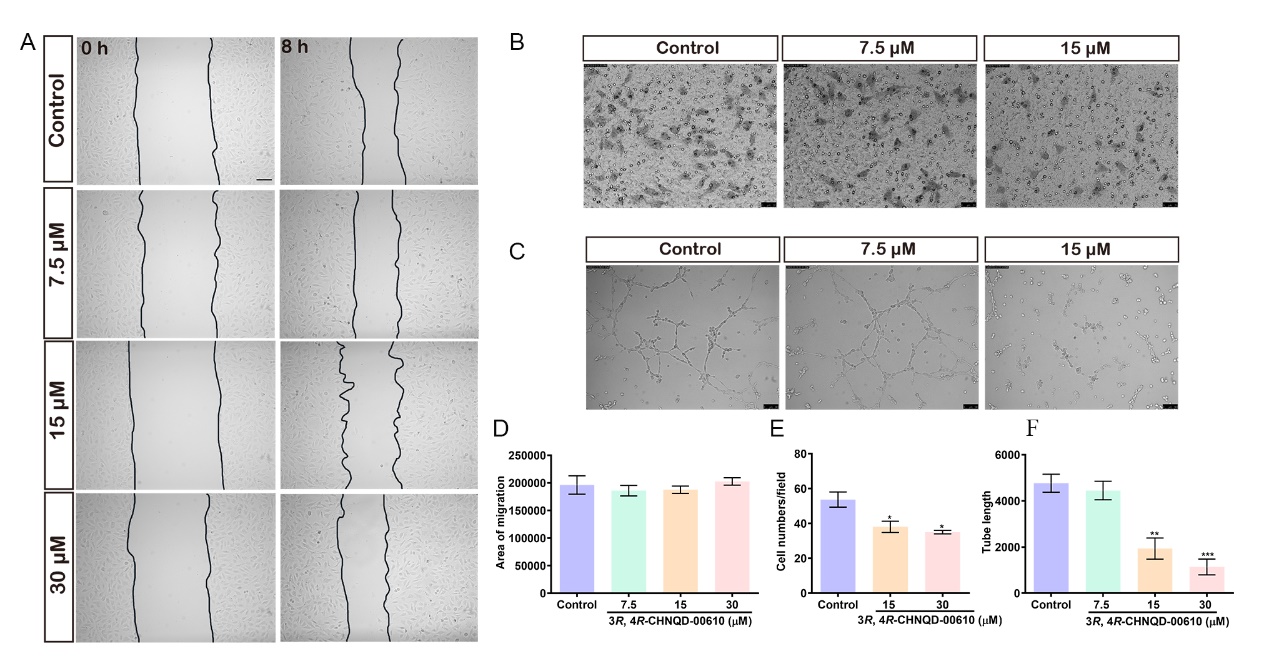


Figure S8. 3*R*, 4*R*-CHNQD-00610 inhibited endothelial migration in EA. hy926. (A, D) Scratch migration and quantification in endothelial cells with 7.5, 15 and 30 μM of 3*R*, 4*R*-CHNQD-00610 treatment for 0 and 8 h. Scale bar, 100 μm. (B, E) Transwell migtation and quantifiction of migration cell number with 3*R*, 4*R*-CHNQD-00610 treatment at concentrations of 7.5 and 15 μM. Scale bar, 50 μm. (C, F) Tube formation and quantification of tube length with 3*R*, 4*R*-CHNQD-00610 treatment at concentrations of 7.5 and 15 μM. Scale bar, 100 μm. Data were represented as means ± SD (n = 3). **p* < 0.1; ***p* < 0.01; ****p* < 0.001.

**Table S1**. Natural products of 3,4-dioxygenated-4-aryl-quinolin-2(1*H*)-one alkaloids and their activities.

| **Natural Product** | **Fungi** | **Chirality of C3, C4** | **Activity** | **References** |
| --- | --- | --- | --- | --- |
| quinolinones A and B;  aflaquinolone A;  aniduquinolones B and C | *Penicillium* | 3*S*, 4*S* | Low toxicity against brine shrimp (*Artemia salina*) | An et al. 2013a; An et al. 2013b; Hayashi et al. 1997; Nakaya, 1995; Neff et al. 2012; |
| yaequinolones D−F;  yaequinolones J1 and J2; | *Penicillium* | 3*S**, 4*S** | Toxicity against brine shrimp (*Artemia salina*) | (Dai et al. 2021; Uchida et al. 2006) |
| penigequinolones A and B | *Penicillium* | 3*S**, 4*S** | Toxicity against brine shrimp (*Artemia salina*); pollen-growth-inhibiting; root-growth-promoting;  nematicidal with selectivity against the root-lesion nematode | (Kimura et al. 1996; Kusano et al. 2000; Uchida et al. 2006) |
| peniprequinolone | *Penicillium* | 3*S**, 4*S** | Toxicity against brine shrimp (*Artemia salina*); root-growth-promoting;  nematicidal with selectivity against the root-lesion nematode; cytotoxicity | (He et al. 2005; Kusano et al. 2000; Schmeda-Hirschmann et al. 2005; Uchida et al. 2006; Wubshet et al. 2013;) |
| yaequinolones A1−A2 | *Penicillium* | 3*R**, 4*S**;  3*S**, 4*S** | Cytotoxicity | (Uchida et al. 2006) |
| aspoquinolones A−B | *Aspergillus* | 3*S**, 4*S** | Cytotoxicity | (Scherlach and Hertweck 2006) |
| aniduquinolone A | *Aspergillus* | 3*S*, 4*S* | Antifouling | (Mou et al. 2017; Shao et al. 2015) |
| aflaquinolone D; 22-*O*-(*N*-Me-*L*-valinyl)-21-*epi*-aflaquinolone B | *Aspergillus* | 3*S*, 4*S* | Antiviral | (Chen et al. 2014) |
| asperalins A−F | *Aspergillus* | 3*R*, 4*S* | Antibacterial | (Hu et al. 2023) |
| pesimquinolones I and J | *Penicillium* | 3*S*, 4*S* | Anti-inflammatory | (Dai et al. 2021; Guo et al. 2023) |
| aflaquinolone I | *Metarhizium* | 3*R*, 4*R* | - | （El-Kashef et al. 2019） |

Figure S9. Structures of 3,4-dioxygenated-4-aryl-quinolin-2(1*H*)-one alkaloids listed in table S1.

**Experimental Section**

## Chemistry

## **General experimental procedures**

Unless otherwise stated, reagents were purchased at the highest commercial quality (>95%) and used without further purification. Optical rotations were measured on a JASCO P-1020 digital polarimeter (JASCO Ltd., Tokyo, Japan). IR spectra were recorded on a Nicolet-Nexus-470 spectrometer (Perkin Elmer Ltd., Boston, MA, USA) using KBr pellets. NMR spectra were recorded on JNM-ECZ600R/S1, Agilent DD2 500 and Bruker Avance NEO 400 NMR spectrometer (600, 500 and 400 MHz for ^1^H NMR, 150, 125 and 100 MHz for ^13^C NMR, respectively). Chemical shifts *δ* were reported in ppm, using TMS as internal standard, and coupling constants (*J*) were in Hz. ESIMS and HRESIMS spectra were obtained from a Micromass Q-TOF spectrometer (Waters Ltd., Boston, MA, USA) and a Thermo Scientific LTQ Orbitrap XL spectrometer (Thermo Fisher Scientific Inc., Waltham, MA, USA). UPLC-MS was performed on Waters UPLC^®^ system (Waters Ltd., Massachusetts, America) using a C18 column [(Waters Ltd., Massachusetts, America) ACQUITY UPLC^®^ BEH C18, 2.1 × 50 mm, 1.7 μm; 0.5 mL/min]. HPLC analysis was performed on a Hitachi L-2000 system (Hitachi Ltd.) using a C18 column [(Eka Ltd.) Kromasil 250 × 10 mm, 5 μm, 2.0 mL/min]. Silica gel (Qingdao Haiyang Chemical Group Co., Qingdao, China; 200–300 mesh) and octadecylsilyl silica gel (YMC Co., Ltd. Tokyo, Japan; 45−60 μm) were used for column chromatography. Yields referred to chromatographically, unless otherwise specified. TLC silica gel plates (Yantai Zhifu Chemical Group Co., Yantai, China; G60, F-254) were used for thin-layer chromatography.

## Bioactivity

### Cell lines

Human umbilical vein endothelial cells HUVEC, EA. hy926, human embryonic kidney cells HEK293T/17, colorectal cancer cells HCT116 and human liver cancer HepG2 cells were from ATCC. HEK293T/17, EA. hy926, HCT116 and HepG2 cells were cultured in medium containing 10% fetal calf serum (FBS), 1% penicillin/streptomycin, 89% DMEM. HUVEC cells were cultured in specific medium, containing 1% penicillin/streptomycin, 1% ECGS, 1% heparin sodium, 10% FBS and 87% Ham's F-12K. All Cell lines were maintained at 37°C in humidified 5% CO_2_ incubator.

### Animals

The *Tg(flk1:EGFP)* transgenic zebrafish used in the experiment was donated by Professor Ting-Xi Liu from Shanghai Jiao Tong University. The *To*(*kras^G12V^*) zebrafish was presented by Professor Zhi-Yuan Gong from the National University of Singapore. Zebrafish were maintained on a 14 h/10 h light/dark cycle at 28 ℃ and fed twice daily. The fertilized zebrafish eggs were staged and maintained according to the standard methods described previously. The embryo medium was supplemented with 0.003% (w/v) 2-phenylthiourea (PTU) to prevent pigmentation. All experiments involving animals were approved by the Institutional Animal Care and Use Committee (IACUC) of the Ocean University of China.

### Plasmids

The pCS2-myc-HIF1α, p2.1 and Renilla plasmids used in this experiment were constructed or retained in the laboratory, and pcDNA3.1-VEGFR2-mCherry was purchased from Addgene.

### *In vivo* anti-angiogenesis assay in zebrafish embryos

Transgenic zebrafish embryos were cultured in a standard environment. Embryos were generated by natural pair mating of adults aged 5 to 12 months and were cultured in embryo culture medium at 28.5°C. After collection, embryos were distributed into 24-well microplates, with 10 embryos per well. When they developed to 18 hpf, embryos were incubated with compounds, sorafenib, or DMSO. Embryos in each well were incubated at 28°C for 28 hpf. Photographs were taken under a fluorescence microscope (Leica DMI6000B, Germany), and intersegmental vessel lengths were measured and statistically analyzed using image Pro Plus 6.

### Western blot analysis

After treatment with (3*R*, 4*R*)-CHNQD-00728 or (3*R*, 4*R*)-CHNQD-00610 at different concentrations for 12 h and VEGF for 15 min, cell extracts were obtained by lysis with RIPA buffer. Protein samples were separated by SDS-PAGE and transfered to a polyvinylidene difluoride (PVDF) membrane. Membranes were incubated with the primary antibodies specific for target proteins overnight at 4℃, and then incubated with the secondary antibody which was coupled to ECL Chemiluminescence Substrate (#BL520A, biosharp).

### Wound healing assay

HUVEC and EA. Hy926 cells were seeded in 12-well plates. When the cells were 100% confluent in the well, injured them using a 200 μl pipette tip, and then wash the cells with D-Hanks solution. Add medium containing 1% FBS and DMSO or different concentrations of compounds to the scratched monolayer. Culture the cells for 0, 12 or 24 h, Place the 12-well plate under the microscope, take pictures and record the wounds. Use the image Pro Plus 6 software to measure the scratch areas at 0, 12 and 24 h, and analyze the results using ImageJ.

### Transwell invasion assay

HUVEC and EA. hy926 cells were starved overnight. A single cell suspension was prepared and counted. To the top chamber, 200 μl of medium containing 1% BSA and 5×10^4^ cells were added. The top chamber contained DMSO or various concentrations of compounds. To the lower chamber, 600 μl of medium containing 1% BSA was added. After 12 hours, the migrated cells were fixed with ethanol, the non-migrated cells were scraped off with cotton swabs, and the migrated cells were then stained with 0.05% crystal violet and photographed under a light microscope.

### Tube formation assay

Matrigel was placed into pre-cooled 96-well plates (50 μl/well) and polymerized at room temperature for 20 minutes, then at 37 ℃ for 30 minutes. HUVEC and EA. hy926 cells (4×10^3^/per well) were added to 96-well plates with DMSO or various concentrations of compounds. After incubation for 8 hours, the tubular structures were observed under a light microscope. Finally, the formation of tubules was quantified by measuring the total length of branches using ImageJ software.

### Real-Time quantitative PCR (qPCR) analysis

Total RNA was extracted after treated with (3*R*, 4*R*)-CHNQD-00728 using the RNAiso plus reagent (Takara Bio). cDNA was made using 500 ng of total RNA with Reverse Transcriptase. qPCR incubations were run with 200 nM of gene-specific primers and Q RT SuperMix for qPCR. Data was analyzed by the 2^-∆∆CT^ method. The specific primers for cDNA synthesis were as follows:

FMNL3 forward 5′-CAGCGAACTTGATGATGAGAAG-3′, reverse 5′-TCTTGTTTTTGGAGCAGATGAG-3′;

HMOX-1, forward 5′-AAGACTGCGTTCCTGCTCAAC-3′, reverse 5′-AAAGCCCTACAGCAACTGTCG-3′;

PDGFBB, forward 5′-GCTGAAAGGGTGGCAACTTC-3′, reverse 5′-GGGAATGAAAAATGGGCGCT-3′;

TGF-β, forward 5′-TGAACCGGCCTTTCCTGCTTCTCATG-3′, reverse 5′-GCGGAAGTCAATGTACAGCTGCCGC-3′;

VEGF, forward 5′-CCTGGTGGACATCTTCCAGGAGTACC-3′, reverse 5′-GAAGCTCATCTCTCCTATGTGCTGGC-3′;

FGF2, forward 5′-GTGTGTGCTAACCGTTACCT-3′, reverse 5′-GCTCTTAGCAGACATTGGAAG-3′;

EPO, forward 5′-GGAGGCCGAGAATATCACGAC-3′, reverse 5′-CCCTGCCAGACTTCTACGG-3′;

ACTIN, forward 5′-AGAGCTACGAGCTGCCTGAC-3′, reverse 5′-AGCACTGTGTTGGCGTACAG-3′.

### Enzyme-linked immunosorbent assay (ELISA)

The concentration of human VEGF protein in 786-O CM or RCC4 CM was determined by a human VEGF Duo-set enzyme linked immune sorbent assay kit (Human VEGF-A Precoated ELISA kit, #1117342, Dakewe biotech) in accordance with the manufacture instructions.

### *In vivo* anticancer assay in zebrafish embryos

Transgenic *To(kras^G12V^)* female and male zebrafish were mated to obtain embryos. Embryos were cultured to 24 hpf in embryo medium containing 2-phenylthiourea (PTU) and embryos were treated with (3*R*, 4*R*)-CHNQD-00728 at concentrations of 5, 10 μM, along with DOX (60 mg/L, Sangon, China) in embryo medium containing 2-phenylthiourea (PTU). The embryo culture medium was refreshed every two days. At 5 and 7 dpf, zebrafish were anesthetized with tracaine (0.08%, w/v) and transferred to methylcellulose (2%, w/v) for photo observation. The results were photographed using a fluorescence microscope (Leica DMI6000B, Leica M205 FCA, Germany) and analyzed using Image Pro Plus 6.

## **Characterization data**

1-Benzyl-4-hydroxy-3-methoxy-4-phenyl-3,4-dihydroquinolin-2(1*H*)-one (**23**), white solid, 72% yield. ^1^H NMR (500 MHz, acetone-*d*_6_) *δ* 7.39 − 7.21 (12H, overlapped), 7.08 (1H, d, *J* = 8.2 Hz), 7.03 (1H, td, *J* = 7.5, 1.1 Hz), 5.27 (1H, d, *J* = 16.3 Hz), 5.15 (1H, d, *J* = 16.3 Hz), 4.85 (1H, s), 4.28 (1H, s), 3.51 (3H, s); ^13^C NMR (125 MHz, acetone-*d*_6_) *δ* 168.1, 142.2, 138.9, 138.0, 131.7, 129.6, 129.3 × 2, 128.9 × 2, 128.7, 128.3, 127.9 × 2, 127.8, 127.6 × 2, 123.9, 116.1, 85.8, 77.0, 59.5, 45.6; ESIMS *m/z* 360.20 [M+H]^+^, 382.21 [M+Na]^+^; HRESIMS *m/z* 360.1585 [M+H]^+^ (calcd for C_23_H_22_O_3_N^+^, 360.1594).

1-(2-Fluorobenzyl)-4-hydroxy-3-methoxy-4-phenyl-3,4-dihydroquinolin-2(1*H*)-one (**24**), white solid, 60% yield. ^1^H NMR (500 MHz, acetone-*d*_6_) *δ* 7.40 (2H, dd, *J* = 8.0, 1.8 Hz), 7.37 − 7.24 (6H, overlapped), 7.16 (1H, m), 7.12 − 7.01 (4H, overlapped), 5.35 (1H, d, *J* = 16.8 Hz), 5.16 (1H, d, *J* = 16.8 Hz), 4.90 (1H, s), 4.34 (1H, s), 3.51 (3H, s); ^13^C NMR (125 MHz, acetone-*d*_6_) *δ* 168.3, 161.2 (d, *J* = 242.9 Hz), 142.1, 138.7, 131.6, 129.8, 129.7 (d, *J* = 8.3 Hz), 128.9 × 2, 128.9 (d, *J* = 3.9 Hz), 128.7, 128.5, 127.8 × 2, 125.2 (d, *J* = 3.4 Hz), 124.7 (d, *J* = 14.2 Hz), 124.1, 115.8 (d, *J* = 21.2 Hz), 115.5, 85.3, 77.0, 59.5, 39.4 (d, *J* = 5.3 Hz); ESIMS *m/z* 378.23 [M+H]^+^, 400.20 [M+Na]^+^; HRESIMS m/z 378.1491 [M+H]^+^ (calcd for C_23_H_21_O_3_NF^+^, 378.1500).

1-(2-Chlorobenzyl)-4-hydroxy-3-methoxy-4-phenyl-3,4-dihydroquinolin-2(1*H*)-one (**25**), white solid, 62% yield. ^1^H NMR (500 MHz, acetone-*d*_6_) *δ* 7.48 (1H, dd, *J* = 8.0, 1.3 Hz), 7.43 (2H, dd, *J* = 8.3, 1.5 Hz), 7.40 − 7.31 (4H, overlapped), 7.31 − 7.24 (2H, overlapped), 7.20 (1H, td, *J* = 7.6, 1.4 Hz), 7.10 − 7.02 (2H, overlapped), 6.86 (1H, dd, *J* = 8.2, 1.2 Hz), 5.38 (1H, d, *J* = 17.3 Hz), 5.06 (1H, d, *J* = 17.3 Hz), 4.92 (1H, s), 4.35 (1H, s), 3.51 (3H, s); ^13^C NMR (125 MHz, acetone-*d*_6_) *δ* 168.3, 142.2, 138.9, 134.9, 133.0, 131.7, 130.3, 129.9, 129.4, 129.0 × 2, 128.7, 128.6, 128.1, 127.9 × 2, 127.8, 124.2, 115.6, 85.4, 77.1, 59.6, 43.9; ESIMS *m/z* 394.15/396.15 [M+H]^+^/[M+2+H]^+^ (3:1); 416.20/418.15 [M+Na]^+^/[M+2+Na]^+^ (3:1); HRESIMS m/z 394.1198 [M+H]^+^ (calcd for C_23_H_21_O_3_NCl^+^, 394.1204).

1-(2-Bromobenzyl)-4-hydroxy-3-methoxy-4-phenyl-3,4-dihydroquinolin-2(1*H*)-one (**26***)*, white solid, 57% yield. ^1^H NMR (500 MHz, acetone-*d*_6_) *δ* 7.66 (1H, dd, *J* = 7.7, 1.5 Hz), 7.45 − 7.41 (2H, overlapped), 7.40 − 7.31 (4H, overlapped), 7.29 − 7.19 (3H, overlapped), 7.08 (1H, td, *J* = 7.5, 1.1 Hz), 7.03 (1H, d, *J* = 7.6 Hz), 6.83 (1H, dd, *J* = 8.1, 1.1 Hz), 5.34 (1H, d, *J* = 17.2 Hz), 5.00 (1H, d, *J* = 17.2 Hz), 4.92 (1H, s), 4.35 (1H, s), 3.51 (3H, s); ^13^C NMR (125 MHz, acetone-*d*_6_) *δ* 168.3, 142.3, 138.9, 136.3, 133.6, 131.7, 129.9, 129.8, 129.0 × 2, 128.8, 128.7, 128.6, 127.9 × 3, 124.2, 122.9, 115.6, 85.4, 77.2, 59.6, 46.6; ESIMS m/z 438.12/440.09 [M+H]^+^/[M+2+H]^+^ (1:1); HRESIMS m/z 438.0687 [M+H]^+^ (calcd for C_23_H_21_O_3_NBr^+^, 438.0699).

4-Hydroxy-3-methoxy-1-(2-methylbenzyl)-4-phenyl-3,4-dihydroquinolin-2(*1H*)-one (**27**), white solid, 54% yield. ^1^H NMR (500 MHz, acetone-*d*_6_) *δ* 7.44 − 7.40 (2H, overlapped), 7.39 − 7.30 (4H, overlapped), 7.25 − 7.20 (2H, overlapped), 7.14 (1H, t, *J* = 7.1 Hz), 7.09 − 7.03 (2H, overlapped), 6.95 (1H, d, *J* = 7.7 Hz), 6.83 (1H, dd, *J* = 8.2, 1.2 Hz), 5.30 (1H, d, *J* = 17.0 Hz), 4.91 (1H, d, *J* = 17.0 Hz), 4.85 (1H, s), 4.28 (1H, s), 3.51 (3H, s), 2.40 (3H, s); ^13^C NMR (125 MHz, acetone-*d*_6_) *δ* 168.0, 142.4, 139.3, 135.9, 135.4, 131.6, 131.0, 129.7, 129.0 × 2, 128.7, 128.3, 127.9 × 2, 127.5, 126.8, 125.4, 123.9, 116.0, 85.6, 77.0, 59.4, 44.2, 19.0; ESIMS *m/z* 374.18 [M+H]^+^, 396.15 [M+Na]^+^; HRESIMS 374.1740 [M+H]^+^ (calcd for C_24_H_24_O_3_N^+^, 396.1570); 396.1558 [M+Na]^+^ (calcd for C_24_H_23_O_3_NNa^+^, 396.1570); 356.1635 [M-H_2_O+H]^+^ (calcd for C_24_H_22_O_2_N^+^, 356.1645).

1-(3-Fluorobenzyl)-4-hydroxy-3-methoxy-4-phenyl-3,4-dihydroquinolin-2(1*H*)-one (**28**), white solid, 70% yield. ^1^H NMR (500 MHz, acetone-*d*_6_) *δ* 7.38 − 7.24 (8H, overlapped), 7.12 (1H, d, *J* = 7.6 Hz), 7.08 (1H, d, *J* = 8.0 Hz), 7.07 − 6.97 (3H, overlapped), 5.31 (1H, d, *J* = 16.5 Hz), 5.17 (1H, d, *J* = 16.5 Hz), 4.87 (1H, s), 4.32 (1H, s), 3.51 (3H, s); ^13^C NMR (125 MHz, acetone-*d*_6_) *δ* 168.4, 163.9 (d, *J* = 242.7 Hz), 142.3, 141.1 (d, *J* = 7.1 Hz), 138.8, 131.8, 131.2 (d, *J* = 8.3 Hz), 129.7, 129.0 × 2, 128.6 (d, *J* = 22.8 Hz), 127.9 × 2, 124.0, 123.6 (d, *J* = 2.7 Hz), 116.0, 114.5, 114.5 (d, *J* = 43.8 Hz), 110.9, 85.4, 76.9, 59.3, 45.1 (d, *J* = 1.7 Hz); ESIMS *m/z* 378.22 [M+H]^+^, 400.21 [M+Na]^+^; HRESIMS *m/z* 378.1490 [M+H]^+^ (calcd for C_23_H_21_O_3_NF^+^, 378.1500).

1-(3-Chlorobenzyl)-4-hydroxy-3-methoxy-4-phenyl-3,4-dihydroquinolin-2(1*H*)-one (**29**), white solid, 50% yield. ^1^H NMR (500 MHz, acetone-*d*_6_) *δ* 7.36 − 7.25 (10H, overlapped), 7.23 (1H, d, *J* = 7.5 Hz), 7.09 (1H, d, *J* = 8.2 Hz), 7.06 (1H, t, *J* = 7.6 Hz), 5.29 (1H, d, *J* = 16.5 Hz), 5.16 (1H, d, *J* = 16.5 Hz), 4.88 (1H, s), 4.29 (1H, s), 3.51 (3H, s); ^13^C NMR (125 MHz, acetone-*d*_6_) *δ* 168.3, 142.2, 140.6, 138.7, 134.8, 131.8, 131.0, 129.7, 129.0 × 2, 128.7, 128.5, 127.9, 127.8 × 2, 127.6, 126.3, 124.1, 115.9, 85.5, 77.1, 59.5, 45.0; ESIMS *m/z* 394.16/396.14 [M+H]^+^/[M+2+H]^+^ (3:1), 416.20/418.16 [M+Na]^+^/[M+2+Na]^+^ (3:1); HRESIMS *m/z* 394.1197 [M+H]^+^ (calcd for C_23_H_21_O_3_NCl^+^, 394.1204).

4-Hydroxy-1-(3-iodobenzyl)-3-methoxy-4-phenyl-3,4-dihydroquinolin-2(1*H*)-one (**30**), white solid, 58% yield. ^1^H NMR (500 MHz, acetone-*d*_6_)^1^H NMR (acetone-*d*_6_, 500 MHz) *δ* 7.48 (1H, dd, *J* = 8.0, 1.1 Hz), 7.44 – 7.41 (2H, overlapped), 7.40 – 7.31 (4H, overlapped), 7.30 – 7.25 (2H, overlapped), 7.20 (1H, td, *J* = 7.7, 1.1 Hz), 7.10 – 7.03 (2H, overlapped), 6.86 (1H, d, *J* = 8.2 Hz), 5.38 (1H, d, *J* = 17.3 Hz), 5.06 (1H, d, *J* = 17.3 Hz), 4.92 (1H, s), 4.35 (1H, s), 3.51 (3H, s); ^13^C NMR (125 MHz, acetone-*d*_6_) *δ* 168.3, 142.2, 138.9, 134.9, 133.0, 131.7, 130.3, 129.9, 129.4, 129.0 × 2, 128.7, 128.6, 128.1, 127.9 × 2, 127.8, 124.2, 115.6, 85.4, 77.1, 59.6, 43.9.

4-Hydroxy-3-methoxy-1-(3-nitrobenzyl)-4-phenyl-3,4-dihydroquinolin-2(1*H*)-one (**31**), white solid, 55% yield. ^1^H NMR (500 MHz, acetone-*d*_6_) *δ* 8.18 (1H, s), 8.13 (1H, d, *J* = 8.1 Hz), 7.70 (1H, d, *J* = 7.3 Hz), 7.60 (1H, t, *J* = 7.9 Hz), 7.32 (6H, overlapped), 7.27 (1H, td, *J* = 7.7, 1.5 Hz), 7.13 (1H, d, *J* = 8.2 Hz), 7.06 (1H, t, *J* = 7.5 Hz), 5.49 (1H, d, *J* = 16.6 Hz), 5.25 (1H, d, *J* = 16.6 Hz), 4.91 (1H, s), 4.32 (1H, s), 3.53 (3H, s); ^13^C NMR (125 MHz, acetone-*d*_6_) *δ* 168.5, 149.4, 142.2, 140.6, 138.6, 134.1, 131.9, 130.7, 129.8, 129.0 × 2, 128.8, 128.6, 127.8 × 2, 124.3, 122.9, 122.5, 115.9, 85.5, 77.1, 59.6, 45.0; ESIMS *m/z* 405.28 [M+H]^+^, 427.20 [M+Na]^+^; HRESIMS *m/z* 405.1440 [M+H]^+^ (calcd for C_23_H_21_O_5_N_2_^+^, 405.1445).

4-Hydroxy-3-methoxy-1-(3-methoxybenzyl)-4-phenyl-3,4-dihydroquinolin-2(1*H*)-one (**32**), white solid, 60% yield. ^1^H NMR (500 MHz, acetone-*d*_6_) *δ* 7.37 − 7.27 (6H, overlapped), 7.25 (1H, t, *J* = 7.8 Hz), 7.21 (1H, t, *J* = 7.9 Hz), 7.08 (1H, d, *J* = 8.2 Hz), 7.03 (1H, t, *J* = 7.5 Hz), 6.88 (1H, s), 6.84 (1H, d, *J* = 7.9 Hz), 6.79 (1H, dd, *J* = 8.2, 2.1 Hz), 5.30 (1H, d, *J* = 16.2 Hz), 5.06 (1H, d, *J* = 16.3 Hz), 4.82 (1H, s), 4.27 (1H, s), 3.72 (3H, s), 3.51 (3H, s); ESIMS *m/z* 390.22 [M+H]^+^, 412.20 [M+Na]^+^; HRESIMS *m/z* 390.1669 [M+H]^+^ (calcd for C_24_H_24_O_4_N^+^, 390.1700).

1-(4-Fluorobenzyl)-4-hydroxy-3-methoxy-4-phenyl-3,4-dihydroquinolin-2(1*H*)-one (**34**), white solid, 65% yield. ^1^H NMR (500 MHz, acetone-*d*_6_) *δ* 7.35 − 7.23 (9H, overlapped), 7.11 (1H, d, *J* = 8.2 Hz), 7.08 − 7.01 (3H, overlapped), 5.25 (1H, d, *J* = 16.2 Hz), 5.16 (1H, d, *J* = 16.2 Hz), 4.84 (1H, s), 4.28 (1H, s), 3.50 (3H, s); ^13^C NMR (125 MHz, acetone-*d*_6_) *δ* 168.3, 162.7 (d, *J* = 241.9 Hz), 142.2, 138.8, 134.0 (d, *J* = 3.0 Hz), 131.8, 129.7, 129.7 (d, *J* = 3.0 Hz) × 2, 128.9 × 2, 128.7, 128.5, 127.9 × 2, 124.0, 116.1 (d, *J* = 5.0 Hz) × 2, 115.9, 85.5, 77.1, 59.5, 44.8; ESIMS *m/z* 378.24 [M+H]^+^, 400.20 [M+Na]^+^; HRESIMS *m/z* 378.1491 [M+H]^+^ (calcd for C_23_H_21_O_3_NF^+^, 378.1500).

1-(4-Bromobenzyl)-4-hydroxy-3-methoxy-4-phenyl-3,4-dihydroquinolin-2(1*H*)-one (**35**), white solid, 65% yield. ^1^H NMR (500 MHz, acetone-*d*_6_) *δ* 7.46 (2H, d, *J* = 8.2 Hz), 7.33 (6H, overlapped), 7.26 (1H, t, *J* = 8.0 Hz), 7.20 (2H, d, *J* = 8.2 Hz), 7.08 (1H, d, *J* = 8.1 Hz), 7.05 (1H, t, *J* = 7.5 Hz), 5.25 (1H, d, *J* = 16.4 Hz), 5.13 (1H, d, *J* = 16.4 Hz), 4.84 (1H, s), 4.29 (1H, s), 3.50 (3H, s); ^13^C NMR (125 MHz, acetone-*d*_6_) *δ* 168.3, 142.2, 138.7, 137.5, 132.3 × 2, 131.8, 129.8× 2, 129.7, 129.0 × 2, 128.7, 128.5, 127.9 × 2, 124.0, 121.2, 116.0, 85.5, 77.1, 59.5, 45.0; ESIMS *m/z* 438.13/440.13 [M+H]^+^/[M+2+H]^+^ (1:1); HRESIMS *m/z* 438.0695 [M+H]^+^ (calcd for C_23_H_21_O_3_NBr^+^, 438.0699).

4-Hydroxy-3-methoxy-1-(4-methylbenzyl)-4-phenyl-3,4-dihydroquinolin-2(1*H*)-one (**36**), white solid, 57% yield. ^1^H NMR (500 MHz, acetone-*d*_6_) *δ* 7.34 − 7.32 (6H, overlapped), 7.24 (1H, t, *J* = 7.1 Hz), 7.14 (2H, d, *J* = 8.0 Hz), 7.11 − 7.07 (3H, overlapped), 7.03 (1H, t, *J* = 7.5 Hz), 5.20 (1H, d, *J* = 16.1 Hz), 5.11 (1H, d, *J* = 16.1 Hz), 4.80 (1H, s), 4.23 (1H, s), 3.50 (3H, s), 2.27 (3H, s); ^13^C NMR (125 MHz, acetone-*d*_6_) *δ* 168.1, 142.3, 139.0, 137.3, 134.9, 131.8, 129.9 × 2, 129.6, 128.9 × 2, 128.7, 128.3, 127.9 × 2, 127.7 × 2, 123.9, 116.2, 85.7, 77.0, 59.4, 45.3, 21.0; ESIMS *m/z* 374.21 [M+H]^+^, 396.21 [M+Na]^+^; HRESIMS *m/z* 374.1743 [M+H]^+^ (calcd for C_24_H_24_O_3_N^+^, 374.1751).

4-Hydroxy-3-methoxy-4-phenyl-1-(4-(trifluoromethoxy)benzyl)-3,4-dihydroquinolin-2(1*H*)-one (**37**), white solid, 62% yield. ^1^H NMR (500 MHz, acetone-*d*_6_) *δ* 7.39 (2H, d, *J* = 8.3 Hz), 7.36 − 7.30 (6H, overlapped), 7.29 − 7.23 (3H, overlapped), 7.11 (1H, d, *J* = 8.2 Hz), 7.05 (1H, t, *J* = 7.5 Hz), 5.32 (1H, d, *J* = 16.4 Hz), 5.19 (1H, d, *J* = 16.4 Hz), 4.86 (1H, s), 4.31 (1H, s), 3.50 (3H, s); ESIMS m/z 444.19 [M+H]^+^, 426.19 [M-H_2_O+H]^+^; HRESIMS m/z 444.1414 [M+H]^+^ (calcd for C_24_H_21_O_4_NF_3_^+^, 444.1417).

1-(2-Chloro-5-(trifluoromethyl)benzyl)-4-hydroxy-3-methoxy-4-phenyl-3,4-dihydroquinolin-2(1*H*)-one (**38**), white solid, 66% yield. ^1^H NMR (500 MHz, acetone-*d*_6_) *δ* 7.76 (1H, d, *J* = 8.3 Hz), 7.67 (1H, d, *J* = 8.0 Hz), 7.45 − 7.39 (4H, overlapped), 7.38 − 7.32 (3H, overlapped), 7.29 (1H, t, *J* = 7.0 Hz), 7.12 (1H, t, *J* = 7.5 Hz), 6.90 (1H, d, *J* = 8.2 Hz), 5.53 (1H, d, *J* = 17.5 Hz), 5.01 (1H, d, *J* = 17.5 Hz), 5.00 (1H, s), 4.26 (1H, s), 3.52 (3H, s); ^13^C NMR (125 MHz, acetone-*d*_6_) *δ* 168.3, 142.1 (d, *J* = 5.7 Hz), 138.7, 137.4, 136.7, 131.8 (d, *J* = 4.8 Hz), 131.5, 130.1, 130.0, 129.9, 129.1 × 2, 128.8, 128.7, 127.8 × 2, 126.4 (dd, *J* = 6.9, 3.0 Hz), 124.3 (dd, *J* = 7.1, 3.2 Hz), 122.3 (dd, *J* = 603.4, 270.4 Hz), 115.4, 85.6, 77.1, 59.5, 44.2; ESIMS *m/z* 462.15/464.11 [M+H]^+^/[M+2+H]^+^ (3:1); HRESIMS *m/z* 462.1068 [M+H]^+^ (calcd for C_24_H_20_O_3_NClF_3_^+^, 462.1078).

1-(3,4-Difluorobenzyl)-4-hydroxy-3-methoxy-4-phenyl-3,4-dihydroquinolin-2(1*H*)-one (**39**), white solid, 55% yield. ^1^H NMR (500 MHz, acetone-*d*_6_) *δ* 7.39 − 7.31 (5H, overlapped), 7.29 (1H, d, *J* = 9.4 Hz, 1H), 7.21 (3H, overlapped), 7.12 (2H, d, *J* = 8.1 Hz), 7.05 (1H, t, *J* = 7.5 Hz), 5.28 (1H, d, *J* = 16.4 Hz), 5.17 (1H, d, *J* = 16.4 Hz), 4.90 (1H, s), 4.37 (1H, s), 3.51 (3H, s); ^13^C NMR (125 MHz, acetone-*d*_6_) *δ* 168.5, 151.4 (dd, *J* = 116.8, 12.9 Hz), 149.4 (d, *J* = 115.9, 12.8 Hz), 142.2, 138.5, 135.7 (dd, *J* = 5.3, 3.5 Hz), 131.8, 129.8, 128.9 × 2, 128.7, 128.6, 127.9 × 2, 124.4 (dd, *J* = 6.6, 3.6 Hz), 124.1, 118.1 (d, *J* = 17.3 Hz), 116.7 (d, *J* = 17.9 Hz), 116.0, 85.3, 77.2, 59.6, 44.5; ESIMS m/z 396.18 [M+H]^+^, 378.22 [M-H_2_O+H]^+^; HRESIMS m/z 396.1398 [M+H]^+^ (calcd for C_23_H_20_O_3_NF_2_^+^, 396.1406).

1-(4-Chloro-2-fluorobenzyl)-4-hydroxy-3-methoxy-4-phenyl-3,4-dihydroquinolin-2(1*H*)-one (**40**), white solid, 53%. ^1^H NMR (500 MHz, acetone-*d*_6_) *δ* ^1^H NMR (500 MHz, acetone-*d*_6_) *δ* 7.40 − 7.26 (8H, overlapped), 7.11 (1H, dd, *J* = 8.4, 2.0 Hz), 7.09 – 7.02 (3H, overlapped), 5.30 (1H, d, *J* = 16.9 Hz), 5.13 (1H, d, *J* = 16.9 Hz), 4.89 (1H, s), 4.33 (1H, s), 3.49 (3H, s); ^13^C NMR (125 MHz, acetone-*d*_6_) *δ* 168.5, 161.2 (d, *J* = 247.1 Hz), 142.2, 138.6, 134.0 (d, *J* = 10.1 Hz), 131.8, 130.2 (d, *J* = 5.1 Hz), 129.9, 129.0 × 2, 128.7, 128.6, 127.9 × 2, 125.5 (d, *J* = 3.7 Hz), 124.2, 124.0 (d, *J* = 14.7 Hz), 116.7 (d, *J* = 25.1 Hz), 115.5, 85.3, 77.1, 59.6, 39.2 (dd, *J* = 4.9 Hz); ESIMS m/z 412.15/414.15 [M+H]^+^/[M+2+H]^+^ (3:1).

1-(4-Bromo-2-fluorobenzyl)-4-hydroxy-3-methoxy-4-phenyl-3,4-dihydroquinolin-2(1*H*)-one (**41**), white solid, 55% yield. ^1^H NMR (500 MHz, acetone-*d*_6_) *δ* 7.42 (1H, dd, *J* = 9.8, 2.0 Hz), 7.40 − 7.27 (7H, overlapped), 7.25 (1H, d, *J* = 8.3 Hz), 7.08 − 7.03 (2H, overlapped), 7.00 (1H, t, *J* = 8.2 Hz), 5.29 (1H, d, *J* = 16.9 Hz), 5.11 (1H, d, *J* = 16.9 Hz), 4.88 (1H, s), 4.33 (1H, s), 3.49 (3H, s); ^13^C NMR (125 MHz, acetone-*d*_6_) *δ* 168.4, 161.2 (d, *J* = 248.1 Hz), 142.2, 138.6, 131.8, 130.5 (d, *J* = 4.7 Hz), 129.9, 129.0 × 2, 128.7, 128.6, 128.5 (d, *J* = 3.7 Hz), 127.9 ×2, 124.5 (d, *J* = 14.3 Hz), 124.2, 121.4 (d, *J* = 9.5 Hz), 119.5 (d, *J* = 24.8 Hz), 115.5, 85.3, 77.1, 59.6, 39.2 (d, *J* = 4.8 Hz); ESIMS *m/z* 456.06/458.07 [M+H]^+^/[M+2+H]^+^ (1:1).

1-(2,6-Difluorobenzyl)-4-hydroxy-3-methoxy-4-phenyl-3,4-dihydroquinolin-2(1*H*)-one (**42**), white solid, 60% yield. ^1^H NMR (500 MHz, acetone-*d*_6_) *δ* 7.38 − 7.30 (3H, overlapped), 7.29 − 7.23 (6H, overlapped), 7.06 (1H, td, *J* = 7.5, 1.2 Hz), 6.96 (2H, t, *J* = 8.2 Hz), 5.43 (1H, d, *J* = 15.7 Hz), 5.16 (1H, d, *J* = 15.7 Hz), 4.69 (1H, s), 4.15 (1H, s), 3.47 (3H, s); ^13^C NMR (125 MHz, acetone-*d*_6_) *δ* 167.2, 163.3 (d, *J* = 7.9 Hz), 161.3 (d, *J* = 8.0 Hz), 142.1, 138.7, 131.9, 130.7 (t, *J* = 10.4 Hz), 129.5, 128.8 × 2, 128.6, 128.4, 127.8 × 2, 124.0, 115.3 (t, *J* = 2.5 Hz), 113.4 (t, *J* = 17.8 Hz), 112.4 (d, *J* = 5.6 Hz), 112.2 (d, *J* = 5.6 Hz), 85.6, 76.7, 59.3, 34.9 (t, *J* = 3.3 Hz); ESIMS *m/z* 396.19 [M+H]^+^; 418.17[M+Na]^+^; HRESIMS m/z 396.1400 [M+H]^+^ (calcd for C_23_H_20_O_3_NF_2_^+^, 396.1406).

1-(2,6-Dichlorobenzyl)-4-hydroxy-3-methoxy-4-phenyl-3,4-dihydroquinolin-2(1*H*)-one (**43**), white solid, 53% yield. ^1^H NMR (500 MHz, acetone-*d*_6_) *δ* 7.37 (4H, d, *J* = 8.0 Hz), 7.34 − 7.25 (5H, overlapped), 7.21 (1H, d, *J* = 7.7 Hz), 7.17 (1H, d, *J* = 7.1 Hz), 7.02 (1H, td, *J* = 7.4, 1.1 Hz), 5.50 (1H, d, *J* = 15.8 Hz), 5.34 (1H, d, *J* = 15.8 Hz), 4.65 (1H, s), 4.29 (1H, s), 3.45 (3H, s); ^13^C NMR (125 MHz, acetone-*d*_6_) *δ* 168.1, 142.7, 139.3, 136.3 × 2, 133.0, 131.9, 130.3, 129.9 × 2, 129.5, 128.9, 128.8 × 2, 128.5, 128.0 × 2, 123.8, 116.0, 85.2, 76.9, 59.7, 43.2; ESIMS m/z 428.14/430.09 [M+H]^+^/[M+2+H]^+^ (9:6); HRESIMS m/z 428.0809 [M+H]^+^ (calcd for C_23_H_20_O_3_NCl_2_^+^, 428.0815).

1-(2,5-Difluorobenzyl)-4-hydroxy-3-methoxy-4-phenyl-3,4-dihydroquinolin-2(1*H*)-one (**44**), white solid, 50% yield. ^1^H NMR (500 MHz, acetone-*d*_6_) *δ* 7.44 − 7.39 (2H, overlapped), 7.38 − 7.26 (5H, overlapped), 7.21 (1H, dt, *J* = 9.3, 4.4 Hz), 7.09 − 7.02 (3H, overlapped), 6.83 (1H, m), 5.33 (1H, d, *J* = 17.0 Hz), 5.16 (1H, d, *J* = 17.0 Hz), 4.94 (1H, s), 4.40 (1H, s), 3.51 (3H, s); ^13^C NMR (125 MHz, acetone-*d*_6_) *δ* 168.7, 159.8 (dd, *J* = 239.5, 2.1 Hz), 157.4 (dd, *J* = 239.1, 2.4 Hz), 142.2, 138.6, 131.8, 130.0, 129.0 × 2, 128.8, 128.7, 127.8 × 2, 127.0 (dd, *J* = 9.5, 7.6 Hz), 124.3, 117.5 (dd, *J* = 72.5, 8.9 Hz), 116.0 (dd, *J* = 24.4, 8.7 Hz), 115.5, 115.3 (dd, *J* = 25.5, 4.4 Hz), 85.2, 77.3, 59.7, 39.4 (d, *J* = 4.8 Hz); ESIMS *m/z* 396.19 [M+H]^+^; 418.17[M+Na]^+^; HRESIMS m/z 396.1396 [M+H]^+^ (calcd for C_23_H_20_O_3_NF_2_^+^, 396.1406).

1-(3,5-Difluorobenzyl)-4-hydroxy-3-methoxy-4-phenyl-3,4-dihydroquinolin-2(1*H*)-one (**45**), white solid, 63% yield. ^1^H NMR (500 MHz, acetone-*d*_6_) *δ* 7.37 − 7.33 (5H, overlapped), 7.29 − 7.23 (2H, overlapped), 7.09 (1H, d, *J* = 8.1 Hz), 7.05 (1H, t, *J* = 7.5 Hz), 6.95 – 6.86 (3H, overlapped), 5.34 (1H, d, *J* = 16.7 Hz), 5.19 (1H, d, *J* = 16.7 Hz), 4.92 (1H, s), 4.38 (1H, s), 3.50 (3H, s); ^13^C NMR (125 MHz, acetone-*d*_6_) *δ* 168.6, 165.0 (d, *J* = 12.8 Hz), 163.0 (d, *J* = 13.0 Hz), 143.01, 143.0 (d, *J* = 8.9 Hz), 138.6, 131.8, 129.9, 129.0 × 2, 128.7 × 2, 127.9 × 2, 124.2, 115.9, 110.7 (d, *J* = 6.2 Hz), 110.6 (d, *J* = 6.2 Hz), 103.1 (t, *J* = 25.6 Hz), 85.2, 77.3, 59.6, 44.9; ESIMS m/z 396.18 [M+H]^+^, 378.19 [M-H_2_O+H]^+^; HRESIMS m/z 396.1401 [M+H]^+^ (calcd for C_23_H_20_O_3_NF_2_^+^, 396.1406).

4-Hydroxy-3-methoxy-4-phenyl-1-(2,4,5-trifluorobenzyl)-3,4-dihydroquinolin-2(1*H*)-one (**46**), white solid, 50% yield. ^1^H NMR (500 MHz, acetone-*d*_6_) *δ* 7.40 (2H, d, *J* = 7.2 Hz), 7.38 − 7.28 (5H, overlapped), 7.25 (1H, m), 7.07 (2H, dd, *J* = 14.4, 8.0 Hz), 6.99 (1H, dd, *J* = 18.1, 8.6 Hz), 5.29 (1H, d, *J* = 16.8 Hz), 5.16 (1H, d, *J* = 16.8 Hz), 4.96 (1H, d, *J* = 1.1 Hz), 4.43 (1H, s), 3.50 (3H, s); ^13^C NMR (125 MHz, acetone-*d*_6_) *δ* 168.8, 155.4 (dd, *J* = 10.0, 3.2 Hz), 150.7 (dd, *J* = 12.5, 14.3 Hz), 148.8 (dd, *J* = 12.5, 14.5 Hz), 142.2, 138.4, 131.8, 130.0, 129.0 × 2, 128.8, 128.7, 127.8 × 2, 124.3, 122.0 (dd, *J* = 16.1, 3.8 Hz), 116.9 (dd, *J* = 20.3, 5.7 Hz), 115.5, 106.7 (dd, *J* = 28.0, 21.3 Hz), 85.1, 77.3, 59.7, 38.8 (d, *J* = 3.9 Hz); ESIMS *m/z* 414.11 [M+H]^+^; HRESIMS m/z 436.1128 [M+Na]^+^ (calcd for C_23_H_18_O_3_NF_3_Na^+^, 436.1131); 396.1202 [M-H_2_O+H]^+^ (calcd for C_23_H_17_O_2_NF_3_^+^, 396.1206).

7-Bromo-4-hydroxy-3-methoxy-1-(3-methylbenzyl)-4-phenyl-3,4-dihydroquinolin-2(1*H*)-one (**49**), white solid, 57% yield. ^1^H NMR (400 MHz, CDCl_3_) *δ* 7.69 (1H, d, *J* = 2.4 Hz), 7.36 – 7.26 (4H, overlapped), 7.23 – 7.13 (3H, overlapped), 7.06 (1H, d, *J* = 7.6 Hz), 6.92 (2H, d, *J* = 8.8 Hz), 6.85 (1H, d, *J* = 8.7 Hz), 5.14 (1H, d, *J* = 16.2 Hz), 5.02 (1H, d, *J* = 16.2 Hz), 4.10 (1H, s), 3.59 (3H, s), 3.56 (1H, s), 2.27 (3H, s); ^13^C NMR (100 MHz, CDCl_3_) *δ* 166.5, 138.9, 138.5, 136.7, 135.7, 132.4, 132.1, 130.6, 128.9, 128.7 × 3, 128.3, 127.2, 126.9 × 2, 123.7, 117.1 × 2, 84.4, 75.8, 59.2, 45.6, 21.5; HRESIMS m/z 452.0849 [M+H]^+^ (calcd for C_24_H_23_O_3_NBr^+^, 452.0856); 474.0663 [M+Na]^+^ (calcd for C_24_H_22_O_3_NBrNa^+^, 474.0675); 434.0742 [M-H_2_O+H]^+^ (calcd for C_24_H_21_O_2_NBr^+^, 434.0750).

4-Hydroxy-3-methoxy-7-methyl-1-(3-methylbenzyl)-4-phenyl-3,4-dihydroquinolin-2(1*H*)-one (**50**), white solid, 54% yield. ^1^H NMR (400 MHz, CDCl_3_) *δ* 7.26 (1H, d, *J* = 2.1 Hz), 7.22 – 7.17 (3H, overlapped), 7.17 – 7.11 (2H, overlapped), 7.07 (1H, t, *J* = 7.5 Hz), 6.95 (2H, overlapped), 6.87 (2H, d, *J* = 8.2 Hz), 6.79 (1H, d, *J* = 8.3 Hz,), 5.09 (1H, d, *J* = 16.1 Hz), 4.93 (1H, d, *J* = 16.1 Hz), 4.01 (1H, s), 3.51 (3H, s), 3.39 (1H, s), 2.18 (6H, d, *J* = 4.7 Hz); ^13^C NMR (100 MHz, CDCl_3_) *δ* 166.7, 139.8, 138.4, 136.3, 135.2, 133.7, 129.8, 129.7, 128.6 × 3, 128.5, 128.1, 128.0, 127.3, 127.0 × 2, 123.8, 115.3, 84.8, 76.1, 59.3, 45.6, 21.5, 20.8; HRESIMS m/z 388.1902 [M+H]^+^ (calcd for C_25_H_26_O_3_N^+^, 388.1907); 410.1717 [M+Na]^+^ (calcd for C_25_H_25_O_3_NNa^+^, 410.1727); 370.1796 [M-H_2_O+H]^+^ (calcd for C_25_H_24_O_2_N^+^, 370.1802).

8-Chloro-4-hydroxy-3-methoxy-1-(3-methylbenzyl)-4-phenyl-3,4-dihydroquinolin-2(1*H*)-one (**51**), white solid, 52% yield. ^1^H NMR (400 MHz, CDCl_3_) *δ* 7.40 (1H, d, *J* = 8.2 Hz), 7.29 – 7.23 (3H, overlapped), 7.22 – 7.14 (3H, overlapped), 7.06 – 6.91 (5H, overlapped), 5.05 (2H, dd, *J* = 23.8, 16.2 Hz), 4.11 (1H, s), 3.59 (1H, s), 3.54 (3H, s), 2.26 (3H, s); HRESIMS m/z 408.1354 [M+H]^+^ (calcd for C_24_H_23_O_3_NCl^+^, 408.1361); 430.1172 [M+Na]^+^ (calcd for C_24_H_22_O_3_NClNa^+^, 430.1180).

4-(4-Fluorophenyl)-4-hydroxy-3-methoxy-1-(3-methylbenzyl)-3,4-dihydroquinolin-2(1*H*)-one (**52**)*,* white solid, 52% yield. ^1^H NMR (600 MHz, CDCl_3_) *δ* 7.53 (1H, dd, *J* = 7.6, 1.6 Hz), 7.29 − 7.25 (1H, m), 7.21 − 7.16 (3H, overlapped), 7.12 (1H, td, *J* = 7.5, 1.1 Hz), 7.06 (1H, d, *J* = 7.5 Hz), 7.01 (1H, d, *J* = 7.2 Hz), 6.98 − 6.93 (4H, overlapped), 5.19 (1H, d, *J* = 16.1 Hz), 5.06 (1H, d, *J* = 16.1 Hz), 4.08 (1H, s), 3.61 (3H, s), 3.49 (1H, s), 2.29 (3H, s); ^13^C NMR (150 MHz, CDCl_3_) *δ* 166.7, 162.7 (d, *J* = 96.2 Hz), 138.4, 137.5, 136.1, 135.5, 129.8, 129.4, 128.9 (d, *J* = 8.2 Hz) × 2, 128.6, 128.1, 127.4, 127.3, 124.0, 123.7, 115.5 (d, *J* = 9.1 Hz) × 2, 115.3, 84.6, 75.6, 59.2, 45.5, 21.4; HRESIMS *m*/*z* 392.1662 [M+H]^+^ (calcd for C_24_H_23_O_3_NF^+^, 392.1656).

4-(4-Chlorophenyl)-4-hydroxy-3-methoxy-1-(3-methylbenzyl)-3,4-dihydroquinolin-2(1*H*)-one (**53**), white solid, 53% yield. ^1^H NMR (600 MHz, CDCl_3_) *δ* 7.48 (1H, dd, *J* = 7.8, 1.6 Hz), 7.28 − 7.23 (3H, overlapped), 7.19 − 7.13 (3H, overlapped), 7.10 (1H, td, *J* = 7.5, 1.1 Hz), 7.05 (1H, d, *J* = 7.5 Hz), 7.00 (1H, dd, *J* = 8.2, 1.1 Hz), 6.98 (1H, d, *J* = 7.7 Hz), 6.96 (1H, s), 5.17 (1H, d, *J* = 16.0 Hz), 5.06 (1H, d, *J* = 16.0 Hz), 4.07 (1H, s), 3.59 (3H, s), 3.46 (1H, s), 2.28 (3H, s); ^13^C NMR (150 MHz, CDCl_3_) *δ* 166.6, 138.4, 138.3, 137.6, 136.1, 134.5, 129.5, 129.4, 128.7 × 2, 128.6, 128.4 × 2, 128.2, 127.5, 127.3, 124.1, 123.7, 115.5, 84.4, 75.7, 59.3, 45.6, 21.4; HRESIMS *m*/*z* 408.1367 [M+H]^+^ (calcd for C_24_H_23_O_3_NCl^+^, 408.1361).

4-(4-Bromophenyl)-4-hydroxy-3-methoxy-1-(3-methylbenzyl)-3,4-dihydroquinolin-2(1*H*)-one (**54**), white solid, 52% yield. ^1^H NMR (600 MHz, CDCl_3_) *δ* 7.47 (1H, dd, *J* = 7.8, 1.6 Hz), 7.42 − 7.38 (2H, overlapped), 7.26 (1H, m), 7.17 (1H, t, *J* = 7.6 Hz), 7.13 − 7.08 (3H, overlapped), 7.05 (1H, d, *J* = 7.6 Hz), 7.02 (1H, m), 6.98 (1H, d, *J* = 7.7 Hz), 6.96 (1H, s), 5.17 (1H, d, *J* = 16.2 Hz), 5.06 (1H, d, *J* = 16.2 Hz), 4.07 (1H, s), 3.59 (3H, s), 3.46 (1H, s), 2.28 (3H, s); ^13^C NMR (150 MHz, CDCl_3_) *δ* 166.6, 138.9, 138.4, 137.5, 136.1, 131.7 × 2, 129.5, 129.4, 128.7 × 2, 128.6, 128.1, 127.5, 127.3, 124.0, 123.7, 122.7, 115.5, 84.3, 75.8, 59.3, 45.6, 21.4; HRESIMS *m*/*z* 452.0864 [M+H]^+^ (calcd for C_24_H_23_O_3_NBr^+^, 452.0856).

7-Chloro-4-(2-fluorophenyl)-4-hydroxy-3-methoxy-1-(3-methylbenzyl)-3,4-dihydroquinolin-2(1*H*)-one (**55**), white solid, 52% yield. ^1^H NMR (400 MHz, CDCl_3_) *δ* 7.42 – 7.34 (1H, m), 7.29 – 7.23 (1H, m), 7.21 – 7.06 (5H, overlapped), 7.04 (1H, d, *J* = 7.6 Hz), 6.99 – 6.97 (2H, overlapped), 6.90 (1H, d, *J* = 8.7 Hz), 5.13 (2H, s), 4.66 (1H, s), 3.55 (3H, s), 2.28 (3H, s); ^13^C NMR (100 MHz, CDCl_3_) *δ* 167.7, 160.1 (d, *J* = 246.7 Hz), 138.5, 136.6, 135.9, 130.9 (d, *J* = 8.7 Hz), 129.8, 129.7, 129.5 (d, *J* = 3.2 Hz), 129.0, 128.7, 128.2, 127.8, 127.3, 126.8 (d, *J* = 11.0 Hz), 124.4 (d, *J* = 3.5 Hz), 123.7, 117.2, 116.6 (d, *J* = 49.7 Hz), 81.4 (d, *J* = 5.4 Hz), 75.5 (d, *J* = 3.2 Hz), 60.4, 46.1, 21.5; HRESIMS m/z 426.1266 [M+H]^+^ (calcd for C_24_H_22_O_3_NClF^+^, 426.1267); 448.1084 [M+Na]^+^ (calcd for C_24_H_21_O_3_NClFNa^+^, 448.1086); 408.1159 [M-H_2_O+H]^+^ (calcd for C_24_H_20_O_2_NClF^+^, 408.1161).

7-Chloro-4-(2-chlorophenyl)-4-hydroxy-3-methoxy-1-(3-methylbenzyl)-3,4-dihydroquinolin-2(1*H*)-one (**56**), white solid, 50% yield. ^1^H NMR (400 MHz, CDCl_3_) *δ* 7.54 (1H, dd, *J* = 7.2, 2.4 Hz), 7.45 (1H, dd, *J* = 7.6, 1.8 Hz), 7.39 – 7.30 (2H, overlapped), 7.23 – 7.16 (2H, overlapped), 7.09 – 6.99 (3H, overlapped), 6.97 (1H, d, *J* = 2.5 Hz), 6.92 (1H, d, *J* = 8.8 Hz), 5.17 (3H, s), 3.56 (1H, s), 3.52 (3H, s), 2.31 (3H, s); ^13^C NMR (100 MHz, CDCl_3_) *δ* 168.0, 138.5, 136.9, 136.8, 135.8, 131.9, 131.8, 130.1 × 2, 129.7, 128.9 × 2, 128.7, 128.2, 128.1, 127.3, 127.2, 123.6, 117.3, 79.5, 60.8, 46.1, 29.7, 21.5.

1-Benzyl-7-chloro-4-hydroxy-3-methoxy-4-phenyl-3,4-dihydroquinolin-2(1*H*)-one (**57**), white solid, 53% yield. ^1^H NMR (500 MHz, acetone-*d*_6_) *δ* 7.37 − 7.30 (6H, overlapped), 7.29 − 7.25 (3H, overlapped), 7.23 (3H, t, *J* = 6.7 Hz), 7.11 (1H, d, *J* = 8.8 Hz), 5.24 (1H, d, *J* = 16.3 Hz), 5.14 (1H, d, *J* = 16.3 Hz), 5.06 (1H, s), 4.27 (1H, s), 3.52 (3H, s); ^13^C NMR (125 MHz, acetone-*d*_6_) *δ* 167.9, 141.4, 137.9, 137.6, 134.1, 129.4 × 2, 129.3, 129.2 × 2, 129.1, 128.8, 128.1, 128.0, 127.8 × 2, 127.7 × 2, 117.9, 85.3, 76.8, 59.5, 45.6; ESIMS *m/z* 394.15/396.10 [M+H]^+^/[M+2+H]^+^ (3: 1); HRESIMS *m/z* 394.1198 [M+H]^+^ (calcd for C_23_H_21_O_3_NCl^+^, 394.1204).

2-((7-Chloro-4-hydroxy-3-methoxy-2-oxo-4-phenyl-3,4-dihydroquinolin-1(2*H*)-yl)methyl)benzonitrile (**58**), white solid, 49% yield. ^1^H NMR (500 MHz, acetone-*d*_6_) *δ* 7.83 (1H, d, *J* = 7.7 Hz), 7.57 (1H, t, *J* = 7.7 Hz), 7.48 (1H, t, *J* = 7.6 Hz), 7.44 − 7.30 (7H, overlapped), 7.12 (1H, d, *J* = 7.9 Hz), 7.03 (1H, d, *J* = 9.0 Hz), 5.49 (1H, d, *J* = 17.3 Hz), 5.24 (1H, d, *J* = 17.3 Hz), 5.17 (1H, s), 4.37 (1H, s), 3.50 (3H, s); ^13^C NMR (125 MHz, acetone-*d*_6_) *δ* 168.2, 141.4, 141.3, 141.1, 137.7, 134.2, 133.9, 129.7, 129.3 × 2, 129.2, 129.1, 128.9, 128.5, 127.9 × 2, 127.1, 117.6, 117.5, 111.9, 85.0, 77.0, 59.6, 44.6; ESIMS *m/z* 419.13/421.01 [M+H]^+^/[M+2+H]^+^ (3:1); HRESIMS *m/z* 419.1149 [M+H]^+^ (calcd for C_24_H_20_O_3_N_2_Cl^+^, 419.1157).

7-Chloro-4-hydroxy-3-methoxy-1-(3-nitrobenzyl)-4-phenyl-3,4-dihydroquinolin-2(1*H*)-one (**60**), white solid, 73%. ^1^H NMR (CDCl_3_, 400 MHz) *δ* 8.12 (1H, dt, *J* = 7.5, 2.1 Hz), 8.02 (1H, t, *J* = 1.8 Hz), 7.55 (1H, d, *J* = 2.5 Hz), 7.50 – 7.42 (2H, overlapped), 7.35 – 7.29 (3H, overlapped), 7.22 (3H, overlapped), 6.80 (1H, d, *J* = 8.7 Hz), 5.36 (1H, d, *J* = 16.5 Hz), 5.07 (1H, d, *J* = 16.5 Hz), 4.16 (1H, s), 3.62 (3H, s), 3.54 (1H, s); ^13^C NMR (CDCl_3_, 100 MHz) *δ* 166.7, 148.6, 138.6, 138.1, 135.5, 132.6, 132.1, 129.9 × 2, 129.2, 129.0, 128.8 × 2, 128.2, 126.7 × 2, 122.7, 121.4, 116.0, 84.1, 75.8, 59.4, 44.9; HRESIMS *m*/*z* 439.1051 [M+H]^+^ (calcd for C_23_H_20_O_5_N_2_Cl^+^, 439.1055); *m*/*z* 421.0946 [M-H_2_O+H]^+^ (calcd for C_23_H_18_O_4_N_2_Cl^+^, 421.0950).

7-Chloro-1-(3-fluorobenzyl)-4-hydroxy-3-methoxy-4-phenyl-3,4-dihydroquinolin-2(1*H*)-one (**62**), white solid, 58% yield. ^1^H NMR (500 MHz, acetone-*d*_6_) *δ* 7.36 − 7.31 (7H, overlapped), 7.29 (1H, dd, *J* = 8.6, 2.5 Hz), 7.12 (1H, d, *J* = 8.7 Hz), 7.08 (1H, d, *J* = 7.7 Hz), 7.04 − 6.95 (2H, overlapped), 5.29 (1H, d, *J* = 16.5 Hz), 5.16 (1H, d, *J* = 16.5 Hz), 5.10 (1H, s), 4.32 (1H, s), 3.52 (3H, s); ^13^C NMR (125 MHz, acetone-*d*_6_) *δ* 168.0, 163.8 (d, *J* = 243.3 Hz), 141.4, 140.6 (d, *J* = 7.2 Hz), 137.7, 134.1, 131.3 (d, *J* = 8.4 Hz), 129.4, 129.2 × 2, 129.1, 128.9, 128.3, 127.8 × 2, 123.6 (d, *J* = 2.9 Hz), 117.8, 114.8 (d, *J* = 21.0 Hz), 114.4 (d, *J* = 22.3 Hz), 85.2, 76.9, 59.5, 45.1 (d, *J* = 1.9 Hz); ESIMS *m/z* 412.14/414.15 [M+H]^+^/[M+2+H]^+^ (3:1); HRESIMS *m/z* 412.1100 [M+H]^+^ (calcd for C_23_H_20_O_3_NClF^+^, 412.1110).

7-Chloro-1-(3-chlorobenzyl)-4-hydroxy-3-methoxy-4-phenyl-3,4-dihydroquinolin-2(1*H*)-one (**63**), white solid, 55% yield. ^1^H NMR (500 MHz, acetone-*d*_6_) *δ* 7.38 − 7.25 (10H, overlapped), 7.19 (1H, d, *J* = 7.5 Hz), 7.13 (1H, d, *J* = 8.7 Hz), 5.27 (1H, d, *J* = 16.5 Hz), 5.16 (1H, d, *J* = 16.5 Hz), 5.10 (1H, s), 4.29 (1H, s), 3.52 (3H, s); ^13^C NMR (125 MHz, acetone-*d*_6_) *δ* 168.0, 141.4, 140.1, 137.6, 134.9, 134.1, 131.1, 129.5, 129.2 × 2, 129.1, 129.0, 128.3, 128.1, 127.8 × 2, 127.6, 126.3, 117.8, 85.3, 76.9, 59.5, 45.1; ESIMS *m/z* 428.05/430.06/432.03 [M+H]^+^/[M+2+H]^+^/[M+2+2+H]^+^ (9:6:1); HRESIMS m/z 428.0811 [M+H]^+^ (calcd for C_23_H_20_O_3_NCl_2_^+^, 428.0815).

7-Chloro-1-(4-fluorobenzyl)-4-hydroxy-3-methoxy-4-phenyl-3,4-dihydroquinolin-2(1*H*)-one (**64**), white solid, 62% yield. ^1^H NMR (500 MHz, acetone-*d*_6_) *δ* 7.37 − 7.27 (7H, overlapped), 7.25 (2H, dd, *J* = 8.5, 5.5 Hz), 7.15 (1H, d, *J* = 8.8 Hz), 7.05 (2H, t, *J* = 8.8 Hz), 5.22 (1H, d, *J* = 16.2 Hz), 5.15 (1H, d, *J* = 16.2 Hz), 5.07 (1H, s), 4.28 (1H, s), 3.51 (3H, s); ^13^C NMR (125 MHz, acetone-*d*_6_) *δ* 167.9, 163.8 (d, *J* = 242.3 Hz), 141.4, 137.6, 134.2, 133.6 (d, *J* = 3.2 Hz), 129.8 (d, *J* = 8.1 Hz) × 2, 129.3, 129.2 × 2, 129.1, 128.9, 128.2, 127.8 × 2, 117.9, 116.0 (d, *J* = 21.4 Hz) × 2, 85.3, 76.9, 59.5, 44.8; ESIMS *m/z* 412.09/414.15 [M+H]^+^/[M+2+H]^+^ (3:1); HRESIMS *m/z* 412.1103 [M+H]^+^ (calcd for C_23_H_20_O_3_NClF^+^, 412.1110).

7-Chloro-1-(4-chlorobenzyl)-4-hydroxy-3-methoxy-4-phenyl-3,4-dihydroquinolin-2(1*H*)-one (**65**), white solid, 55% yield. ^1^H NMR (500 MHz, acetone-*d*_6_) *δ* 7.37 − 7.27 (9H, overlapped), 7.22 (2H, d, *J* = 8.5 Hz), 7.12 (1H, d, *J* = 8.7 Hz), 5.24 (1H, d, *J* = 16.4 Hz), 5.15 (1H, d, *J* = 16.4 Hz), 5.08 (1H, s), 4.29 (1H, s), 3.51 (3H, s); ^13^C NMR (125 MHz, acetone-*d*_6_) *δ* 168.0, 141.4, 137.6, 136.6, 134.2, 133.3, 129.5 × 2, 129.4 × 3, 129.2 × 2, 129.1, 128.9, 128.2, 127.8 × 2, 117.8, 85.2, 76.9, 59.5, 44.9; ESIMS *m/z* 428.11/430.11/432.09 [M+H]^+^/[M+2+H]^+^/[M+2+2+H]^+^ (9:6:1); HRESIMS *m/z* 428.0808 [M+H]^+^ (calcd for C_23_H_20_O_3_NCl_2_^+^, 428.0815).

1-(4-Bromobenzyl)-7-chloro-4-hydroxy-3-methoxy-4-phenyl-3,4-dihydroquinolin-2(1*H*)-one (**66**), white solid, 65% yield. ^1^H NMR (500 MHz, acetone-*d*_6_) *δ* 7.45 (2H, d, *J* = 8.4 Hz), 7.37 − 7.26 (7H, overlapped), 7.16 (2H, d, *J* = 8.4 Hz), 7.11 (1H, d, *J* = 8.7 Hz), 5.23 (1H, d, *J* = 16.4 Hz), 5.13 (1H, d, *J* = 16.4 Hz), 5.08 (1H, s), 4.29 (1H, s), 3.50 (3H, s); ^13^C NMR (125 MHz, acetone-*d*_6_) *δ* 168.0, 141.4, 137.6, 137.0, 134.2, 132.4 × 2, 129.8 × 2, 129.4, 129.2 × 2, 129.1, 128.9, 128.2, 127.8 × 2, 121.4, 117.8, 85.2, 76.9, 59.5, 45.0; ESIMS *m/z* 472.04/474.04/476.01 [M+H]^+^/[M+2+H]^+^/[M+2+2+H]^+^ (3:4:1); HRESIMS *m/z* 472.0305 [M+H]^+^ (calcd for C_23_H_20_O_3_NBrCl^+^, 472.0310).

1-(4-(Tert-butyl)benzyl)-7-chloro-4-hydroxy-3-methoxy-4-phenyl-3,4-dihydroquinolin-2(1*H*)-one (**67**), white solid, 51% yield. ^1^H NMR (600 MHz, CDCl_3_) *δ* 7.54 (1H, d, *J* = 2.5 Hz), 7.32 – 7.27 (5H, overlapped), 7.22 – 7.19 (3H, overlapped), 7.07 (2H, d, *J* = 8.3 Hz), 6.94 (1H, d, *J* = 8.7 Hz), 5.21 (1H, d, *J* = 16.1 Hz), 4.98 (1H, d, *J* = 16.1 Hz), 4.09 (1H, s), 3.60 (3H, s), 3.51 (1H, d, *J* = 1.5 Hz), 1.30 (9H, s); ^13^C NMR (150 MHz, CDCl_3_) *δ* 166.4, 150.4, 138.8, 136.3, 132.7, 132.1, 129.4, 129.1, 128.8, 128.6 × 2, 127.7, 126.8 × 2, 126.3 × 2, 125.7 × 2, 116.7, 84.4, 75.8, 59.3, 45.4, 34.5, 31.3 × 3; HRESIMS *m*/*z* 450.1841 [M+H]^+^ (calcd for C_27_H_29_O_3_NCl^+^, 450.1830).

1-(4-Bromo-2-fluorobenzyl)-7-chloro-4-hydroxy-3-methoxy-4-phenyl-3,4-dihydroquinolin-2(1*H*)-one (**69**), white solid, 60% yield. ^1^H NMR (500 MHz, acetone-*d*_6_) *δ* 7.42 (1H, dd, d, *J* = 9.8, 1.9 Hz), 7.38 − 7.30 (7H overlapped), 7.25 (1H, dd, *J* = 8.3 Hz), 7.08 (1H, d, *J* = 8.7 Hz), 6.93 (1H, t, *J* = 8.2 Hz), 5.27 (1H, d, *J* = 16.9 Hz), 5.12 (1H, s), 5.09 (1H, d, *J* = 16.9 Hz), 4.32 (1H, s), 3.50 (3H, s); ^13^C NMR (125 MHz, acetone-*d*_6_) *δ* 168.1, 161.2 (d, *J* = 248.4 Hz), 141.3, 137.5, 134.1, 134.07, 130.4 (d, *J* = 4.7 Hz), 129.6, 129.2 × 2, 129.1, 128.5 (d, *J* = 3.6 Hz), 128.3, 127.8 × 2, 124.1 (d, *J* = 14.4 Hz), 121.6 (d, *J* = 9.7 Hz), 119.6 (d, *J* = 24.7 Hz), 117.4, 85.1, 76.9, 59.6, 39.4 (d, *J* = 4.8 Hz); ESIMS *m/z* 489.98/491.97/494.03 [M+H]^+^/[M+2+H]^+^/[M+2+2+H]^+^ (3:4:1); HRESIMS *m/z* 490.0211 [M+H]^+^ (calcd for C_23_H_19_O_3_NBrClF^+^, 490.0215).

7-Chloro-1-(2-chloro-5-(trifluoromethyl)benzyl)-4-hydroxy-3-methoxy-4-phenyl-3,4-dihydroquinolin-2(1*H*)-one (**70**), white solid, 50% yield. ^1^H NMR (500 MHz, acetone-*d*_6_) *δ* 7.76 (1H, d, *J* = 8.4 Hz), 7.68 (1H, d, *J* = 8.6 Hz), 7.42 (3H, dd, *J* = 8.2, 1.8 Hz), 7.40 − 7.34 (4H, overlapped), 7.32 (1H, dd, *J* = 8.7, 2.5 Hz), 6.95 (1H, d, *J* = 8.7 Hz), 5.53 (1H, d, *J* = 17.6 Hz), 5.24 (1H, s), 4.97 (1H, d, *J* = 17.6 Hz), 4.26 (1H, s), 3.52 (3H, s); ESIMS *m/z* 496.06/498.00/500.02 [M+H]^+^/[M+2+H]^+^/ [M+2+2+H]^+^ (9:6:1); HRESIMS *m/z* 496.0681 [M+H]^+^ (calcd for C_24_H_19_O_3_NCl_2_F_3_^+^, 496.0689).

7-Chloro-1-(3,4-difluorobenzyl)-4-hydroxy-3-methoxy-4-phenyl-3,4-dihydroquinolin-2(1*H*)-one (**71**), white solid, 52% yield. ^1^H NMR (500 MHz, acetone-*d*_6_) *δ* 7.37 − 7.28 (7H, overlapped), 7.24 (1H, m), 7.18 − 7.05 (3H, overlapped), 5.24 (1H, d, *J* = 16.4 Hz), 5.17 (1H, d, *J* = 16.4 Hz), 5.12 (1H, s), 4.34 (1H, s), 3.51 (3H, s); ^13^C NMR (125 MHz, acetone-*d*_6_) *δ* 168.1, 151.5 (dd, *J* = 105.8, 13.2 Hz), 149.5 (dd, *J* = 105.1, 12.8 Hz), 141.4, 137.5, 135.3 (dd, *J* = 5.7, 3.9 Hz), 134.2, 129.5, 129.2 × 2, 129.1, 129.0, 128.3, 127.8 × 2, 124.4 (dd, *J* = 6.5, 3.7 Hz), 118.2 (d, *J* = 17.3 Hz), 117.8, 116.9 (d, *J* = 17.7 Hz), 85.1, 77.0, 59.6, 44.5; ESIMS *m/z* 430.11/432.12 [M+H]^+^/[M+2+H]^+^ (3:1); HRESIMS *m/z* 430.1007 [M+H]^+^ (calcd for C_23_H_19_O_3_NClF_2_^+^, 430.1016).

7-Chloro-1-(3,4-dichlorobenzyl)-4-hydroxy-3-methoxy-4-phenyl-3,4-dihydroquinolin-2(1*H*)-one (**72**), white solid, 55% yield. ^1^H NMR (500 MHz, acetone-*d*_6_) *δ* 7.48 (1H, d, *J* = 8.3 Hz), 7.42 (1H, d, *J* = 1.3 Hz), 7.34 − 7.28 (7H, overlapped), 7.19 (1H, dd, *J* = 8.3, 1.3 Hz), 7.15 (1H, d, *J* = 8.7 Hz), 5.27 (1H, d, *J* = 16.6 Hz), 5.17 (1H, d, *J* = 16.6 Hz), 5.14 (1H, s), 4.32 (1H, s), 3.51 (3H, s); ^13^C NMR (125 MHz, acetone-*d*_6_) *δ* 168.1, 141.3, 138.7, 137.4, 134.1, 132.8, 131.5, 131.4, 129.8, 129.5, 129.2 × 2, 129.1, 129.06, 128.3, 127.9, 127.8 × 2, 117.7, 85.1, 76.9, 59.5, 44.5; ESIMS *m/z* 462.04/464.03/466.01 [M+H]^+^/[M+2+H]^+^/[M+2+2+H]^+^ (3:3:1); HRESIMS *m/z* 462.0420 [M+H]^+^ (calcd for C_23_H_19_O_3_NCl_3_^+^, 462.0425).

7-Chloro-1-(2,6-dichlorobenzyl)-4-hydroxy-3-methoxy-4-phenyl-3,4-dihydroquinolin-2(1*H*)-one (**73**), white solid, 56% yield. ^1^H NMR (500 MHz, acetone-*d*_6_) *δ* 7.38 − 7.27 (10H, overlapped), 7.23 (1H, d, *J* = 8.7 Hz), 5.47 (1H, d, *J* = 15.8 Hz), 5.30 (1H, d, *J* = 15.8 Hz), 4.90 (1H, s), 4.27 (1H, s), 3.45 (3H, s); ^13^C NMR (125 MHz, acetone-*d*_6_) *δ* 167.7, 141.8, 138.2, 136.4, 134.3 × 2, 132.7, 130.5, 129.9 × 2, 129.1 × 3, 128.9, 128.7, 128.5, 128.0 × 2, 117.8, 85.0, 76.6, 59.6, 43.4; ESIMS *m/z* 462.01/464.01/466.04 [M+H]^+^/[M+2+H]^+^/[M+2+2+H]^+^ (3:3:1); HRESIMS m/z 484.0244 [M+Na]^+^ (calcd for C_23_H_18_O_3_NCl_3_Na^+^, 484.0244); 444.0320 [M-H_2_O+H]^+^ (calcd for C_23_H_17_O_2_NCl_3_^+^, 444.0319).

1-(3,5-Bis(trifluoromethyl)benzyl)-7-chloro-4-hydroxy-3-methoxy-4-phenyl-3,4-dihydroquinolin-2(1*H*)-one (**74**), white solid, 51% yield. ^1^H NMR (500 MHz, acetone-*d*_6_) *δ* 7.95 − 7.93 (3H, overlapped), 7.40 − 7.32 (6H, overlapped), 7.31 (1H, d, *J* = 8.6 Hz), 7.20 (1H, d, *J* = 8.7 Hz), 5.63 (1H, d, *J* = 16.9 Hz), 5.26 (1H, d, *J* = 16.9 Hz), 5.24 (1H, s), 4.35 (1H, s), 3.53 (3H, s); ESIMS *m/z* 530.00/531.80 [M+H]^+^/[M+2+H]^+^ (3:1); HRESIMS m/z 530.0950 [M+H]^+^ (calcd for C_25_H_19_O_3_NClF_6_^+^, 530.0952); 512.0844 [M-H_2_O+H]^+^ (calcd for C_25_H_19_O_2_NClF_6_^+^, 512.0847).

7-Chloro-1-(4-chloro-2-fluorobenzyl)-4-hydroxy-3-methoxy-4-phenyl-3,4-dihydroquinolin-2(1*H*)-one (**75**), white solid, 52% yield. ^1^H NMR (500 MHz, acetone-*d*_6_) *δ* 7.39 − 7.31 (7H, overlapped), 7.29 (1H, dd, *J* = 10.0, 2.0 Hz), 7.12 (1H, dd, *J* = 16.9, 1.6 Hz), 7.08 (1H, d, *J* = 9.1 Hz), 6.99 (1H, t, *J* = 8.3 Hz), 5.28 (1H, d, *J* = 16.9 Hz), 5.11 (2H, d, *J* = 16.9 Hz), 4.32 (1H, s), 3.50 (3H, s); ^13^C NMR (125 MHz, acetone-*d*_6_) *δ* 168.1, 161.2 (d, *J* = 247.3 Hz), 141.3, 137.5, 134.2, 134.1, 130.1 (d, *J* = 5.1 Hz), 129.6, 129.2 × 2, 129.1× 2, 128.3, 127.8 ×2, 125.6 (d, *J* = 3.6 Hz), 123.6 (d, *J* = 14.7 Hz), 117.4 (d, *J* = 1.0 Hz), 116.8 (d, *J* = 25.0 Hz), 85.1, 76.9, 59.5, 39.3 (d, *J* = 4.9 Hz); ESIMS *m/z* 446.18/448.03/450.14 [M+H]^+^/[M+2+H]^+^/[M+2+2+H]^+^ (9:6:1); HRESIMS *m/z* 446.0719 [M+H]^+^ (calcd for C_23_H_19_O_3_NCl_2_F^+^, 446.0721).

1-Benzyl-3-methoxy-4-phenylquinolin-2(1*H*)-one (**76**), white solid, 64% yield. ^1^H NMR (500 MHz, acetone-*d*_6_) *δ* 7.57 (2H, t, *J* = 7.3 Hz), 7.50 (1H, m), 7.46 (1H, d, *J* = 8.6 Hz), 7.42 − 7.38 (3H, overlapped), 7.37 − 7.32 (4H, overlapped), 7.27 (1H, m), 7.16 (1H, dd, *J* = 8.0, 1.4 Hz), 7.11 (1H, m), 5.70 (2H, s), 3.82 (3H, s); ^13^C NMR (125 MHz, acetone-*d*_6_) *δ* 159.7, 145.8, 138.2, 137.9, 137.5, 134.8, 130.3 × 2, 129.6, 129.5 × 2, 129.2 × 2, 128.9, 128.0, 127.9, 127.6 × 2, 123.0, 122.1, 116.0, 60.1, 46.4; HRESIMS m/z 342.1482 [M+H]^+^ (calcd for C_23_H_20_O_2_N^+^, 342.1489).

3-Methoxy-1-(2-methylbenzyl)-4-phenylquinolin-2(1*H*)-one (**77**), white solid, 67% yield. ^1^H NMR (600 MHz, CDCl_3_) *δ* 7.61 − 7.59 (2H, overlapped), 7.54 (1H, m), 7.48 − 7.45 (2H, overlapped), 7.38 (1H, td, *J* = 7.2, 1.4 Hz), 7.34 (1H, d, *J* = 8.1 Hz), 7.31 (1H, d, *J* = 6.6 Hz), 7.22 (1H, t, *J* = 7.1 Hz), 7.16 − 7.09 (3H, overlapped), 6.77 (1H, d, *J* = 7.7 Hz), 5.64 (2H, s), 3.88 (3H, s), 2.58 (3H, s); ^13^C NMR (150 MHz, CDCl_3_) *δ* 159.3, 144.9, 137.7, 136.6, 134.8, 133.5, 133.4, 130.4, 129.5 × 2, 128.9, 128.4 × 2, 128.2, 127.4, 127.0, 126.3, 124.6, 122.4, 121.4, 114.9, 60.3, 44.6, 19.2; HRESIMS *m*/*z* 356.1650 [M+H] ^+^ (calcd for C_24_H_22_O_2_N^+^, 356.1645).

1-(2-Fluorobenzyl)-3-methoxy-4-phenylquinolin-2(1*H*)-one (**78**), white solid, 62% yield. ^1^H NMR (500 MHz, acetone-*d*_6_) *δ* 7.59 – 7.54 (2H, overlapped), 7.51 (1H, t, *J* = 7.5 Hz), 7.45 (1H, m), 7.41 − 7.37 (3H, overlapped), 7.33 (1H, t, *J* = 7.2 Hz), 7.26 − 7.17 (2H, overlapped), 7.15 (1H, t, *J* = 7.2 Hz), 7.09 (1H, t, *J* = 7.5 Hz), 7.01 (1H, t, *J* = 7.6 Hz), 5.72 (2H, s), 3.81 (3H, s); ^13^C NMR (125 MHz, acetone-*d*_6_) *δ* 161.3 (d, *J* = 243.0 Hz), 159.7, 145.8, 138.3, 137.4, 134.8, 130.3 × 2, 130.0 (d, *J* = 8.3 Hz), 129.8, 129.2 × 2, 128.9, 128.9 (d, *J* = 4.0 Hz), 128.0, 125.5 (d, *J* = 3.4 Hz), 124.6 (d, *J* = 5.6 Hz), 123.2, 122.2, 116.2 (d, *J* = 21.3 Hz), 115.4, 60.1, 40.4 (d, *J* = 5.6 Hz); ESIMS *m/z* 360.22 [M+H]^+^, 382.20 [M + Na]^+^; HRESIMS m/z 360.1387 [M+H]^+^ (calcd for C_23_H_19_O_2_NF^+^, 360.1394).

1-(2-Iodobenzyl)-3-methoxy-4-phenylquinolin-2(1*H*)-one (**79**), white solid, 52% yield. ^1^H NMR (500 MHz, acetone-*d*_6_) *δ* 8.02 (1H, dd, *J* = 7.9, 1.3 Hz), 7.59 – 7.56 (2H, overlapped), 7.51 (1H, t, *J* = 7.4 Hz), 7.45 − 7.41 (3H, overlapped), 7.28 (1H, t, *J* = 7.6 Hz), 7.21 (1H, dd, *J* = 8.1, 1.7 Hz), 7.16 (1H, m), 7.11 (1H, d, *J* = 8.6 Hz), 7.08 (1H, t, *J* = 7.6 Hz), 6.74 (1H, d, *J* = 7.8 Hz), 5.57 (2H, s), 3.80 (3H, s); ^13^C NMR (125 MHz, acetone-*d*_6_) *δ* 159.5, 145.8, 140.5, 138.7, 138.4, 137.4, 134.8, 130.3 × 2, 130.0, 129.9, 129.6, 129.3 × 2, 129.0, 128.0, 127.3, 123.3, 122.2, 115.7, 97.8, 60.2, 52.4; ESIMS *m/z* 468.10 [M+H]^+^; HRESIMS m/z 468.0444 [M+H]^+^ (calcd for C_23_H_19_O_2_NI^+^, 468.0455).

1-(3-Fluorobenzyl)-3-methoxy-4-phenylquinolin-2(1*H*)-one (**81**), white solid, 52% yield. ^1^H NMR (500 MHz, acetone-*d*_6_) *δ* 7.58 − 7.55 (2H, overlapped), 7.51 − 7.48 (1H, m), 7.46 − 7.36 (5H, overlapped), 7.22 – 7.16 (2H, overlapped), 7.15 − 7.10 (2H, overlapped), 7.02 (1H, td, *J* = 8.5, 2.5 Hz), 5.72 (2H, s), 3.82 (3H, s); ^13^C NMR (125 MHz, acetone-*d*_6_) *δ* 163.9 (d, *J* = 243.2 Hz), 159.7, 145.8, 140.9 (d, *J* = 7.5 Hz), 138.3, 137.4, 134.8, 131.4 (d, *J* = 8.1 Hz), 130.3 × 2, 129.7, 129.2 × 2, 128.9, 128.0, 123.6 (d, *J* = 2.8 Hz), 123.2, 122.2, 115.8, 114.8 (d, *J* = 21.0 Hz), 114.5 (d, *J* = 22.3 Hz), 60.1, 46.0 (d, *J* = 2.0 Hz); ESIMS *m/z* 360.17 [M+H]^+^, 382.17 [M+Na]^+^; HRESIMS m/z 360.1388 [M+H]^+^ (calcd for C_23_H_19_O_2_NF^+^, 360.1394).

3-Methoxy-1-(3-methoxybenzyl)-4-phenylquinolin-2(1*H*)-one (**82**), white solid, 58% yield. ^1^H NMR (500 MHz, acetone-*d*_6_) *δ* 7.57 − 7.54 (2H, overlapped), 7.52 − 7.47 (1H, m), 7.46 (1H, d, *J* = 8.7 Hz), 7.41 − 7.38 (3H, overlapped), 7.24 (1H, t, *J* = 7.9 Hz), 7.16 (1H, dd, *J* = 8.2, 1.4 Hz), 7.11 (1H, m), 6.94 (1H, s), 6.89 (1H, d, *J* = 7.7 Hz), 6.83 (1H, dd, *J* = 8.2, 2.2 Hz), 5.67 (2H, s), 3.82 (3H, s), 3.76 (3H, s); ^13^C NMR (125 MHz, acetone-*d*_6_) *δ* 161.0, 159.6, 145.8, 139.5, 138.2, 137.5, 134.8, 130.6, 130.3 × 2, 129.5, 129.2 × 2, 128.8, 127.8, 123.0, 122.1, 119.6, 115.9, 113.7, 112.9, 60.1, 55.4, 46.3; ESIMS *m/z* 372.18 [M+H]^+^, 394.14 [M+Na]^+^; HRESIMS m/z 372.1591 [M+H]^+^ (calcd for C_24_H_22_O_3_N^+^, 372.1594).

3-Methoxy-1-(4-methylbenzyl)-4-phenylquinolin-2(1*H*)-one (**83**), white solid, 52% yield. ^1^H NMR (500 MHz, acetone-*d*_6_) *δ* 7.58 − 7.54 (2H, overlapped), 7.51 − 7.46 (2H, overlapped), 7.41 − 7.38 (3H, overlapped), 7.24 (2H, d, *J* = 7.8 Hz), 7.15 (3H, d, *J* = 7.4 Hz), 7.11 (1H, t, *J* = 7.5 Hz), 5.65 (2H, s), 3.81 (3H, s), 2.28 (3H, s); ^13^C NMR (125 MHz, acetone-*d*_6_) *δ* 159.7, 145.9, 138.1, 137.5 × 2, 134.9 × 2, 130.3 × 2, 130.1 × 2, 129.5, 129.2 × 2, 128.9, 127.8, 127.7 × 2, 123.0, 122.1, 116.0, 60.1, 46.2, 21.0; ESIMS *m/z* 356.19 [M+H]^+^, 378.19 [M+Na]^+^; HRESIMS m/z 356.1597 [M+H]^+^ (calcd for C_24_H_22_O_2_N^+^, 356.1645).

1-(4-Fluorobenzyl)-3-methoxy-4-phenylquinolin-2(1*H*)-one (**84**), white solid, 67% yield. ^1^H NMR (500 MHz, acetone-*d*_6_) *δ* 7.58 – 7.53 (2H, overlapped), 7.51 − 7.47 (2H, overlapped), 7.43 − 7.38 (5H, overlapped), 7.16 (1H, d, *J* = 7.5 Hz), 7.13 − 7.09 (3H, overlapped), 5.68 (2H, s), 3.81 (3H, s); ^13^C NMR (125 MHz, acetone-*d*_6_) *δ* 162.8 (d, *J* = 242.9 Hz), 159.7, 145.8, 138.2, 137.4, 134.8, 134.0, 130.3 × 2, 129.8 (d, *J* = 8.1 Hz) × 2, 129.6, 129.2 × 2, 128.9, 127.9, 123.1, 122.2, 116.2 (d, *J* = 21.5 Hz) × 2, 115.8, 60.1, 45.7; HRESIMS m/z 360.1385 [M+H]^+^ (calcd for C_23_H_19_O_2_NF^+^, 360.1394).

3-Methoxy-4-phenyl-1-(4-(trifluoromethyl)benzyl)quinolin-2(1*H*)-one (**85**), white solid, 50% yield. ^1^H NMR (500 MHz, acetone-*d*_6_) *δ* 7.70 − 7.69 (2H, overlapped), 7.58 − 7.54 (4H, overlapped), 7.50 (1H, m), 7.45 − 7.37 (4H, overlapped), 7.19 (1H, dd, *J* = 8.2, 1.0 Hz), 7.12 (1H, m), 5.80 (2H, s), 3.82 (3H, s); ^13^C NMR (125 MHz, acetone-*d*_6_) *δ* 159.7, 145.8, 142.7 (dd, *J* = 2.4, 1.1 Hz), 138.3, 137.3, 134.7, 130.3 × 2, 129.8, 129.7 (dd, *J* = 63.9, 31.8 Hz) × 2, 129.2 × 2, 128.9, 128.4 × 2, 128.0, 126.4 (dd, *J* = 8.2, 1.0 Hz), 125.2 (d, *J* = 377.3, 107.1 Hz), 123.2, 122.2, 115.7, 60.1, 46.1; ESIMS *m/z* 410.22 [M+H]^+^; HRESIMS m/z 410.1360 [M+H]^+^ (calcd for C_24_H_19_O_2_NF_3_^+^, 410.1362).

1-([1,1'-Biphenyl]-4-ylmethyl)-3-methoxy-4-phenylquinolin-2(1*H*)-one (**86**), white solid, 50% yield. ^1^H NMR (500 MHz, acetone-*d*_6_) *δ* 7.65 − 7.61 (4H, overlapped), 7.59 − 7.54 (2H, overlapped), 7.53 − 7.48 (2H, overlapped), 7.46 − 7.43 (4H, overlapped), 7.43 − 7.39 (3H, overlapped), 7.34 (1H, t, *J* = 7.5 Hz), 7.17 (1H, dd, *J* = 8.1, 1.4 Hz), 7.12 (1H, m), 5.75 (2H, s), 3.83 (3H, s); ^13^C NMR (125 MHz, acetone-*d*_6_) *δ* 159.7, 145.8, 141.3, 140.8, 138.2, 137.5, 137.1, 134.8, 130.3 × 2, 129.7 × 2, 129.6, 129.2 × 2, 128.9, 128.3 × 2, 128.2, 128.0 × 2, 127.9, 127.6 × 2, 123.1, 122.2, 116.0, 60.1, 46.2; ESIMS *m/z* 418.19 [M+H]^+^, 440.19 [M+Na]^+^; HRESIMS m/z 418.1801 [M+H]^+^ (calcd for C_29_H_24_O_2_N^+^, 418.1802); 440.1619 [M+Na]^+^ (calcd for C_29_H_23_O_2_NNa^+^, 440.1621).

1-(3,5-Bis(trifluoromethyl)benzyl)-3-methoxy-4-phenylquinolin-2(1*H*)-one (**87**), white solid, 59% yield. ^1^H NMR (500 MHz, acetone-*d*_6_) *δ* 8.08 (2H, s), 7.99 (1H, s), 7.57 − 7.55 (3H, overlapped), 7.50 (1H, t, *J* = 7.5 Hz), 7.45 (1H, td, *J* = 7.8, 1.4 Hz), 7.39 (2H, d, *J* = 7.0 Hz), 7.20 (1H, dd, *J* = 7.8, 1.1 Hz), 7.16 (1H, t, *J* = 7.5 Hz), 5.92 (2H, s), 3.82 (3H, s); ^13^C NMR (125 MHz, acetone-*d*_6_) *δ* 159.9, 145.8, 141.7, 138.6, 137.3, 134.6, 132.3 (dd, *J* = 66.3, 33.1 Hz) × 2, 130.3 × 2, 129.9, 129.3 × 2, 129.0, 128.7 (dd, *J* = 7.0, 3.7 Hz) × 2, 128.2, 124.3 (dd, *J* = 541.4, 270.8 Hz) × 2, 123.5, 122.3, 122.1 (tt, *J* = 3.8 Hz), 115.4, 60.1, 46.1; ESIMS *m/z* 478.05 [M+H]^+^; HRESIMS m/z 478.1230 [M+H]^+^ (calcd for C_25_H_18_O_2_NF_6_^+^, 478.1236); 500.1050 [M+Na]^+^ (calcd for C_25_H_17_O_2_NF_6_Na^+^, 500.1056).

4-(4-Fluorophenyl)-3-methoxy-1-(3-methylbenzyl)quinolin-2(1*H*)-one (**88**), white solid, 55% yield. ^1^H NMR (600 MHz, CDCl_3_) *δ* 7.39 − 7.34 (4H, overlapped), 7.26 − 7.20 (4H, overlapped), 7.13 (1H, s), 7.12 − 7.06 (3H, overlapped), 5.62 (2H, s), 3.84 (3H, s), 2.33 (3H, s); ^13^C NMR (150 MHz, CDCl_3_) *δ* 162.5 (d, *J* = 246.2 Hz), 159.3, 145.2, 138.5, 136.6 × 2, 136.1, 131.3 (d, *J* = 8.0 Hz) × 2, 129.3 (d, *J* = 3.6 Hz), 128.9, 128.6, 128.1, 127.4, 127.1, 123.7, 122.3, 121.3, 115.5 (d, *J* = 21.4 Hz) × 2, 115.0, 60.1, 46.5, 21.4; HRESIMS *m*/*z* 374.1556 [M+H]^+^ (calcd for C_24_H_21_O_2_NF^+^, 374.1551).

4-(4-Chlorophenyl)-3-methoxy-1-(3-methylbenzyl)quinolin-2(1*H*)-one (**89**), white solid, 57% yield. ^1^H NMR (600 MHz, CDCl_3_) *δ* 7.53 − 7.50 (2H, overlapped), 7.39 − 7.31 (4H, overlapped), 7.24 − 7.20 (2H, overlapped), 7.13 (1H, d, *J* = 2.2 Hz), 7.11 − 7.06 (3H, overlapped), 5.61 (2H, s), 3.85 (3H, s), 2.33 (3H, s); ^13^C NMR (150 MHz, CDCl_3_) *δ* 159.2, 145.1, 138.5, 136.6, 136.3, 136.1, 134.2, 131.9, 130.9 × 2, 128.9, 128.7 × 2, 128.6, 128.1, 127.3, 127.0, 123.7, 122.4, 121.0, 115.0, 60.2, 46.5, 21.4; HRESIMS *m*/*z* 390.1263 [M+H]^+^ (calcd for C_24_H_21_O_2_NCl^+^, 390.1255).

4-(4-Bromophenyl)-3-methoxy-1-(3-methylbenzyl)quinolin-2(1*H*)-one (**90**), white solid, 60% yield. ^1^H NMR (600 MHz, CDCl_3_) *δ* 7.68 − 7.64 (2H, m), 7.37 (1H, td, *J* = 8.3, 6.7 Hz), 7.34 (1H, dd, *J* = 8.6, 1.4 Hz), 7.27 (1H, m), 7.26 (1H, m), 7.24 − 7.20 (2H, overlapped), 7.12 (1H, s), 7.11 − 7.06 (3H, overlapped), 5.61 (2H, s), 3.84 (3H, s), 2.33 (3H, s); ^13^C NMR (150 MHz, CDCl_3_) *δ* 159.3, 145.0, 138.6, 136.6, 136.4, 136.1, 132.4, 131.7 × 2, 131.2 × 2, 129.0, 128.7, 128.2, 127.4, 127.1, 123.8, 122.4 × 2, 121.0, 115.0, 60.2, 46.6, 21.4; HRESIMS *m*/*z* 434.0761 [M+H]^+^ (calcd for C_24_H_21_O_2_NBr^+^, 434.0750).

1-(2-Bromobenzyl)-7-chloro-3-methoxy-4-phenylquinolin-2(1*H*)-one (**91**), white solid, 72% yield. ^1^H NMR (500 MHz, acetone-*d*_6_) *δ* 7.74 (1H, m), 7.60 (2H, t, *J* = 7.5 Hz), 7.54 (1H, t, *J* = 7.5 Hz), 7.47 − 7.42 (3H, overlapped), 7.28 − 7.23 (2H, overlapped), 7.20 (1H, d, *J* = 9.1 Hz), 7.14 (1H, d, *J* = 2.4 Hz), 6.81 (1H, dd, *J* = 5.7, 3.7 Hz), 5.65 (2H, s), 3.83 (3H, s); ^13^C NMR (125 MHz, acetone-*d*_6_) *δ* 159.3, 146.7, 137.2, 136.1, 135.7, 134.1, 133.8, 130.3 × 2, 130.0, 129.5 × 3, 129.3, 128.9, 128.3, 127.8, 126.8, 123.9, 123.0, 117.6, 60.3, 47.5; ESIMS *m/z* 454.05/456.05/458.02 [M+H]^+^/[M+2+H]^+^/[M+2+2+H]^+^ (3:4:1); HRESIMS *m/z* 454.0204 [M+H]^+^ (calcd for C_23_H_18_O_2_NBrCl^+^, 454.0204).

7-Chloro-3-methoxy-1-(2-methylbenzyl)-4-phenylquinolin-2(1*H*)-one (**92**), white solid, 54% yield. ^1^H NMR (600 MHz, CDCl_3_) *δ* 7.61 – 7.57 (2H, overlapped), 7.54 (1H, m), 7.42 (1H, d, *J* = 1.5 Hz), 7.41 (1H, m), 7.29 (1H, d, *J* = 2.3 Hz), 7.28 (1H, d, *J* = 2.4 Hz), 7.26 (1H, d, *J* = 2.4 Hz), 7.21 (1H, t, *J* = 7.5 Hz), 7.09 (1H, t, *J* = 7.3 Hz), 7.01 (1H, d, *J* = 8.9 Hz), 6.70 (1H, d, *J* = 7.7 Hz), 5.58 (2H, s), 3.86 (3H, s), 2.54 (3H, s); ^13^C NMR (150 MHz, CDCl_3_) *δ* 158.9, 145.7, 136.5, 135.0, 134.8, 133.0, 132.8, 130.5, 129.4 × 2, 128.8, 128.6 × 2, 128.5, 128.0, 127.2, 126.4 × 2, 124.5, 122.8, 116.4, 60.3, 44.7, 19.2; HRESIMS *m*/*z* 390.1263 [M+H]^+^ (calcd for C_24_H_21_O_2_NCl^+^, 390.1255).

3-((7-Chloro-3-methoxy-2-oxo-4-phenylquinolin-1(2*H*)-yl)methyl)benzonitrile (**93**), white solid, 52% yield. ^1^H NMR (500 MHz, acetone-*d*_6_) *δ* 7.79 (1H, s), 7.70 (2H, t, *J* = 9.2 Hz), 7.59 (3H, t, *J* = 7.0 Hz), 7.53 (1H, q, *J* = 6.5 Hz), 7.49 (1H, d, *J* = 9.0 Hz), 7.44 − 7.41 (3H, overlapped), 7.11 (1H, d, *J* = 2.4 Hz), 5.77 (2H, s), 3.84 (3H, s); ^13^C NMR (125 MHz, acetone-*d*_6_) *δ* 159.5, 146.8, 139.4, 137.1, 136.0, 134.1, 132.4, 131.9, 131.3, 130.8, 130.2 × 2, 129.5 × 2, 129.4, 129.3, 128.2, 126.8, 124.0, 119.1, 117.6, 113.5, 60.3, 46.1; ESIMS *m/z* 401.14/403.09 [M+H]^+^/[M+2+H]^+^ (3:1); HRESIMS *m/z* 401.1046 [M+H]^+^ (calcd for C_24_H_18_O_2_N_2_Cl^+^, 401.1051).

7-Chloro-3-methoxy-1-(4-methylbenzyl)-4-phenylquinolin-2(1*H*)-one (**95**), white solid, 72% yield. ^1^H NMR (400 MHz, CDCl_3_) *δ* 7.54 (3H, overlapped), 7.36 (2H, d, *J* = 6.5 Hz), 7.28 (2H, overlapped), 7.22 – 7.12 (5H, overlapped), 5.60 (2H, s), 3.84 (3H, s), 2.34 (3H, s); ^13^C NMR (100 MHz, CDCl_3_) *δ* 159.1, 145.8, 137.2, 136.6, 135.0, 132.8 × 2, 129.5 × 2, 129.3 × 2, 128.7, 128.6 × 2, 128.5, 127.9, 126.7 × 2, 126.4, 122.8, 116.3, 60.3, 46.4, 21.1; HRESIMS m/z 390.1250 [M+H]^+^ (calcd for C_24_H_21_O_2_NCl^+^, 390.1255); 412.1062 [M+Na]^+^ (calcd for C_24_H_20_O_2_NClNa^+^, 412.1075).

7-Chloro-3-methoxy-4-phenyl-1-(4-(trifluoromethyl)benzyl)quinolin-2(1*H*)-one (**96**), white solid, 56% yield. ^1^H NMR (500 MHz, acetone-*d*_6_) *δ* 7.70 (2H, d, *J* = 8.1 Hz), 7.61 − 7.55 (4H, overlapped), 7.53 (1H, m), 7.46 (1H, d, *J* = 9.0 Hz), 7.44 − 7.38 (3H, overlapped), 7.12 (1H, d, *J* = 2.4 Hz), 5.80 (2H, s), 3.84 (3H, s); ^13^C NMR (125 MHz, acetone-*d*_6_) *δ* 159.4, 146.8, 142.3 (d, *J* = 1.6 Hz), 137.1, 136.1, 134.1, 130.2 × 2, 129.8 (d, *J* = 31.9 Hz), 129.5 × 2, 129.4, 129.3, 128.4 × 2, 128.2, 126.8, 126.5 (dd, *J* = 7.7, 3.8 Hz) × 2, 125.2 (dd, *J* = 428.3, 158.4 Hz), 123.9, 117.6, 60.3, 46.4; ESIMS *m/z* 444.12/446.08 [M+H]^+^/[M+2+H]^+^ (3:1); HRESIMS *m/z* 444.0964 [M+H]^+^ (calcd for C_24_H_18_O_2_NClF_3_^+^, 444.0973).

1-(4-(Tert-butyl)benzyl)-7-chloro-3-methoxy-4-phenylquinolin-2(1*H*)-one (**97**), white solid, 51% yield. ^1^H NMR (600 MHz, CDCl_3_) *δ* 7.56 − 7.52 (2H, overlapped), 7.50 (1H, m), 7.36 − 7.32 (4H, overlapped), 7.29 (1H, d, *J* = 2.1 Hz), 7.29 (1H, s), 7.21 (2H, d, *J* = 8.4 Hz), 7.18 (1H, d, *J* = 1.4 Hz), 5.58 (2H, s), 3.82 (3H, s), 1.29 (9H, s); ^13^C NMR (150 MHz, CDCl_3_) *δ* 159.1, 150.5, 145.9, 136.5, 135.1, 132.9, 132.8, 129.4 × 2, 128.8, 128.7 × 2, 128.5, 127.9, 126.5 × 3, 125.8 × 2, 122.9, 116.3, 60.3, 46.3, 34.5, 31.3 × 3; HRESIMS *m*/*z* 432.1733 [M+H]^+^ (calcd for C_27_H_27_O_2_NCl^+^, 432.1725).

7-Chloro-1-(3,5-difluorobenzyl)-3-methoxy-4-phenylquinolin-2(1*H*)-one (**98**), white solid, 62% yield. ^1^H NMR (500 MHz, acetone-*d*_6_) *δ* 7.62 − 7.56 (2H, overlapped), 7.53 (1H, m), 7.47 (1H, d, *J* = 9.0 Hz), 7.44 − 7.42 (3H, overlapped), 7.11 (1H, d, *J* = 2.4 Hz), 7.01 (2H, overlapped), 6.94 (1H, tt, *J* = 9.2, 2.3 Hz), 5.73 (2H, s), 3.84 (3H, s); ^13^C NMR (125 MHz, acetone-*d*_6_) *δ* 165.2 (d, *J* = 12.9 Hz), 163.2 (d, *J* = 12.9 Hz), 159.4, 146.8, 142.4 (t, *J* = 9.0 Hz), 137.2, 136.0, 134.1, 130.3 × 2, 129.5 × 2, 129.4, 129.2, 128.3, 126.8, 124.0, 117.6, 110.8 (d, *J* = 6.1 Hz), 110.7 (d, *J* = 6.1 Hz), 103.6 (t, *J* = 25.6 Hz), 60.3, 46.1 (t, *J* = 2.3 Hz); ESIMS *m/z* 412.11/414.11 [M+H]^+^/[M+2+H]^+^ (3:1); HRESIMS *m/z* 412.0903 [M+H]^+^ (calcd for C_23_H_17_O_2_NClF_2_^+^, 412.0910).

1-(3,5-Bis(trifluoromethyl)benzyl)-7-chloro-3-methoxy-4-phenylquinolin-2(1*H*)-one (**99**), white solid, 66% yield. ^1^H NMR (500 MHz, acetone-*d*_6_) *δ* 8.07 (2H, s), 8.00 (1H, s), 7.63 − 7.55 (3H, overlapped), 7.52 (1H, m), 7.45 − 7.38 (3H, overlapped), 7.14 (1H, d, *J* = 2.5 Hz), 5.91 (2H, s), 3.85 (3H, s); ^13^C NMR (125 MHz, acetone-*d*_6_) *δ* 159.7, 146.8, 141.2, 137.3, 136.0, 134.0, 132.4 (dd, *J* = 66.3, 33.2 Hz) × 2, 130.2 × 2, 129.5 × 3, 129.3, 128.6 (dd, *J* = 7.2, 3.4 Hz) × 2, 128.4, 127.0, 124.3 (dd, *J* = 541.5, 270.8 Hz) × 2, 124.0, 122.2 (tt, *J* = 3.9 Hz), 117.4, 60.3, 46.4; ESIMS *m/z* 512.05/513.98 [M+H]^+^/[M+2+H]^+^ (3:1); HRESIMS *m/z* 512.0839 [M+H]^+^ (calcd for C_25_H_17_O_2_NClF_6_^+^, 512.0847).

7-Chloro-1-(2-chloro-4-fluorobenzyl)-3-methoxy-4-phenylquinolin-2(1*H*)-one (**100**), white solid, 57% yield. ^1^H NMR (500 MHz, acetone-*d*_6_) *δ* 7.63 − 7.57 (2H, overlapped), 7.54 (1H, m), 7.47 − 7.38 (4H, overlapped), 7.26 (1H, d, *J* = 9.0 Hz), 7.14 (1H, d, *J* = 1.5 Hz), 7.03 (1H, td, *J* = 8.4, 2.6 Hz), 6.91 (1H, m), 5.66 (2H, s), 3.82 (3H, s); ^13^C NMR (125 MHz, acetone-*d*_6_) *δ* 162.6 (d, *J* = 246.5 Hz), 159.3, 146.7, 137.2, 136.0, 134.1, 133.9, 130.5 (d, *J* = 3.4 Hz), 130.3 × 2, 129.5 × 2, 129.5 (d, *J* = 6.5 Hz), 129.3, 129.3 (d, *J* = 9.0 Hz), 128.3, 126.9, 123.9, 117.8 (d, *J* = 25.2 Hz), 117.5, 115.4 (d, *J* = 21.3 Hz), 60.3, 44.5; ESIMS *m/z* 428.16/430.07/432.12 [M+H]^+^/[M+2+H]^+^/[M+2+2+H]^+^ (9:6:1); HRESIMS *m/z* 428.0609 [M+H]^+^ (calcd for C_23_H_17_O_2_NCl_2_F^+^, 428.0615).

## Spectra of compounds

**Figure S10.** ^1^H NMR (500 MHz, acetone-*d*_6_) spectrum of compound **23**.

**Figure S11.** ^13^C NMR (125 MHz, acetone-*d*_6_) spectrum of compound **23**.

**Figure S12.** HRESIMS spectrum of compound **23**.

**Figure S13.** ^1^H NMR (500 MHz, acetone-*d*_6_) spectrum of compound **24**.

**Figure S14.** ^13^C NMR (125 MHz, acetone-*d*_6_) spectrum of compound **24**.

**Figure S15.** HRESIMS spectrum of compound **24**.

**Figure S16.** ^1^H NMR (500 MHz, acetone-*d*_6_) spectrum of compound **25**.

**Figure S17.** ^13^C NMR (125 MHz, acetone-*d*_6_) spectrum of compound **25**.

**Figure S18.** HRESIMS spectrum of compound **25**.

**Figure S19.** ^1^H NMR (500 MHz, acetone-*d*_6_) spectrum of compound **26**.

**Figure S20.** ^13^C NMR (125 MHz, acetone-*d*_6_) spectrum of compound **26**.

**Figure S21.** HRESIMS spectrum of compound **26**.

**Figure S22.** ^1^H NMR (500 MHz, acetone-*d*_6_) spectrum of compound **27**.

**Figure S23.** ^13^C NMR (125 MHz, acetone-*d*_6_) spectrum of compound **27**.

**Figure S24.** ESIMS spectrum of compound **27**.

**Figure S25.** ^1^H NMR (500 MHz, acetone-*d*_6_) spectrum of compound **28**.

**Figure S26.** ^13^C NMR (125 MHz, acetone-*d*_6_) spectrum of compound **28**.

**Figure S27.** HRESIMS spectrum of compound **28**.

**Figure S28.** ^1^H NMR (500 MHz, acetone-*d*_6_) spectrum of compound **29**.

**Figure S29.** ^13^C NMR (125 MHz, acetone-*d*_6_) spectrum of compound **29**.

**Figure S30.** HRESIMS spectrum of compound **29**. **Figure S31.** ^1^H NMR (500 MHz, acetone-*d*_6_) spectrum of compound **30**. **Figure S32.** ^13^C NMR (125 MHz, acetone-*d*_6_) spectrum of compound **30**.

**Figure S33.** ^1^H NMR (500 MHz, acetone-*d*_6_) spectrum of compound **31**.

**Figure S34.** ^13^C NMR (125 MHz, acetone-*d*_6_) spectrum of compound **31**.

**Figure S35.** HRESIMS spectrum of compound **31**.

**Figure S36.** ^1^H NMR (500 MHz, acetone-*d*_6_) spectrum of compound **32**.

**Figure S37.** HRESIMS spectrum of compound **32**.

**Figure S38.** ^1^H NMR (500 MHz, acetone-*d*_6_) spectrum of compound **33**.

**Figure S39.** ^13^C NMR (125 MHz, acetone-*d*_6_) spectrum of compound **33**.

**Figure S40.** HRESIMS spectrum of compound **33**.

**Figure S41.** ^1^H NMR (500 MHz, acetone-*d*_6_) spectrum of compound **34**.

**Figure S42.** ^13^C NMR (125 MHz, acetone-*d*_6_) spectrum of compound **34**.

**Figure S43.** HRESIMS spectrum of compound **34**.

**Figure S44.** ^1^H NMR (500 MHz, acetone-*d*_6_) spectrum of compound **35**.

**Figure S45.** ^13^C NMR (125 MHz, acetone-*d*_6_) spectrum of compound **35**.

**Figure S46.** HRESIMS spectrum of compound **35**.

**Figure S47.** ^1^H NMR (500 MHz, acetone-*d*_6_) spectrum of compound **36**.

**Figure S48.** ^13^C NMR (125 MHz, acetone-*d*_6_) spectrum of compound **36**.

**Figure S49.** HRESIMS spectrum of compound **36**.

**Figure S50.** ^1^H NMR (500 MHz, acetone-*d*_6_) spectrum of compound **37**.

**Figure S51.** HRESIMS spectrum of compound **37**.

**Figure S52.** ^1^H NMR (500 MHz, acetone-*d*_6_) spectrum of compound **38**.

**Figure S53.** ^13^C NMR (125 MHz, acetone-*d*_6_) spectrum of compound **38**.

**Figure S54.** HRESIMS spectrum of compound **38**.

**Figure S55.** ^1^H NMR (500 MHz, acetone-*d*_6_) spectrum of compound **39**.

**Figure S56.** ^13^C NMR (125 MHz, acetone-*d*_6_) spectrum of compound **39**.

**Figure S57.** HRESIMS spectrum of compound **39**.

**Figure S58.** ^1^H NMR (500 MHz, acetone-*d*_6_) spectrum of compound **40**.

**Figure S59.** ^13^C NMR (125 MHz, acetone-*d*_6_) spectrum of compound **40**.

**Figure S60.** ESIMS spectrum of compound **40**.

**Figure S61.** ^1^H NMR (500 MHz, acetone-*d*_6_) spectrum of compound **41**.

**Figure S62.** ^13^C NMR (125 MHz, acetone-*d*_6_) spectrum of compound **41**.

**Figure S63.** ESIMS spectrum of compound **41**.

**Figure S64.** ^1^H NMR (500 MHz, acetone-*d*_6_) spectrum of compound **42**.

**Figure S65.** ^13^C NMR (125 MHz, acetone-*d*_6_) spectrum of compound **42**

**Figure S66.** HRESIMS spectrum of compound **42**.

**Figure S67.** ^1^H NMR (500 MHz, acetone-*d*_6_) spectrum of compound **43**.

**Figure S68.** ^13^C NMR (125 MHz, acetone-*d*_6_) spectrum of compound **43**.

**Figure S69.** HRESIMS spectrum of compound **43**.

**Figure S70.** ^1^H NMR (500 MHz, acetone-*d*_6_) spectrum of compound **44**.

**Figure S71.** ^13^C NMR (125 MHz, acetone-*d*_6_) spectrum of compound **44**.

**Figure S72.** HRESIMS spectrum of compound **44**.

**Figure S73.** ^1^H NMR (500 MHz, acetone-*d*_6_) spectrum of compound **45**.

**Figure S74.** ^13^C NMR (125 MHz, acetone-*d*_6_) spectrum of compound **45**.

**Figure S75.** HRESIMS spectrum of compound **45**.

**Figure S76.** ^1^H NMR (500 MHz, acetone-*d*_6_) spectrum of compound **46**.

**Figure S77.** ^13^C NMR (125 MHz, acetone-*d*_6_) spectrum of compound **46**.

**Figure S78.** ESIMS spectrum of compound **46**.

**Figure S79.** ^1^H NMR (400 MHz, CDCl_3_) spectrum of compound **47**.

**Figure S80.** ^13^C NMR (100 MHz, CDCl_3_) spectrum of compound **47**.

**Figure S81.** HRESIMS spectrum of compound **47**.

**Figure S82.** ^1^H NMR (600 MHz, CDCl_3_) spectrum of compound **48**.

**Figure S83.** ^13^C NMR (150 MHz, CDCl_3_) spectrum of compound **48**.

**Figure S84.** HRESIMS spectrum of compound **48**.

**Figure S85.** ^1^H NMR (400 MHz, CDCl_3_) spectrum of compound **49**.

**Figure S86.** ^13^C NMR (100 MHz, CDCl_3_) spectrum of compound **49**.

**Figure S87.** HRESIMS spectrum of compound **49**.

**Figure S88.** ^1^H NMR (400 MHz, CDCl_3_) spectrum of compound **50**.

**Figure S89.** ^13^C NMR (100 MHz, CDCl_3_) spectrum of compound **50**.

**Figure S90.** HRESIMS spectrum of compound **50**.

**Figure S91.** ^1^H NMR (400 MHz, CDCl_3_) spectrum of compound **51**.

**Figure S92.** HRESIMS spectrum of compound **51**.

**Figure S93.** ^1^H NMR (600 MHz, CDCl_3_) spectrum of compound **52**.

**Figure S94.** ^13^C NMR (150 MHz, CDCl_3_) spectrum of compound **52**.

**Figure S95.** HRESIMS spectrum of compound **52**.

**Figure S96.** ^1^H NMR (600 MHz, CDCl_3_) spectrum of compound **53**.

**Figure S97.** ^13^C NMR (150 MHz, CDCl_3_) spectrum of compound **53**.

**Figure S98.** HRESIMS spectrum of compound **53**.

**Figure S99.** ^1^H NMR (600 MHz, CDCl_3_) spectrum of compound **54**.

**Figure S100.** ^13^C NMR (150 MHz, CDCl_3_) spectrum of compound **54**.

**Figure S101.** HRESIMS spectrum of compound **54**.

**Figure S102.** ^1^H NMR (400 MHz, CDCl_3_) spectrum of compound **55**.

**Figure S103.** ^13^C NMR (100 MHz, CDCl_3_) spectrum of compound **55**.

**Figure S104.** HRESIMS spectrum of compound **55**.

**Figure S105.** ^1^H NMR (400 MHz, CDCl_3_) spectrum of compound **56**.

**Figure S106.** ^13^C NMR (100 MHz, CDCl_3_) spectrum of compound **56**.

**Figure S107.** ^1^H NMR (500 MHz, acetone-*d*_6_) spectrum of compound **57**.

**Figure S108.** ^13^C NMR (125 MHz, acetone-*d*_6_) spectrum of compound **57**.

**Figure S109.** HRESIMS spectrum of compound **57**.

**Figure S110.** ^1^H NMR (500 MHz, acetone-*d*_6_) spectrum of compound **58**.

**Figure S111.** ^13^C NMR (125 MHz, acetone-*d*_6_) spectrum of compound **58**.

**Figure S112.** HRESIMS spectrum of compound **58**.

**Figure S113.** ^1^H NMR (600 MHz, CDCl_3_) spectrum of compound **59**.

**Figure S114.** ^13^C NMR (150 MHz, CDCl_3_) spectrum of compound **59**.

**Figure S115.** HRESIMS spectrum of compound **59**.

**Figure S116.** ^1^H NMR (400 MHz, CDCl_3_) spectrum of compound **60**.

**Figure S117.** ^13^C NMR (100 MHz, CDCl_3_) spectrum of compound **60**.

**Figure S118.** HRESIMS spectrum of compound **60**.

**Figure S119.** ^1^H NMR (500 MHz, acetone-*d*_6_) spectrum of compound **61**.

**Figure S120.** ^13^C NMR (125 MHz, acetone-*d*_6_) spectrum of compound **61**.

**Figure S121.** HRESIMS spectrum of compound **61**.

**Figure S122.** ^1^H NMR (500 MHz, acetone-*d*_6_) spectrum of compound **62**.

**Figure S123.** ^13^C NMR (125 MHz, acetone-*d*_6_) spectrum of compound **62**.

**Figure S124.** HRESIMS spectrum of compound **62**.

**Figure S125.** ^1^H NMR (500 MHz, acetone-*d*_6_) spectrum of compound **63**.

**Figure S126.** ^13^C NMR (125 MHz, acetone-*d*_6_) spectrum of compound **63**.

**Figure S127.** HRESIMS spectrum of compound **63**.

**Figure S128.** ^1^H NMR (500 MHz, acetone-*d*_6_) spectrum of compound **64**.

**Figure S129.** ^13^C NMR (125 MHz, acetone-*d*_6_) spectrum of compound **64**.

**Figure S130.** HRESIMS spectrum of compound **64**.

**Figure S131.** ^1^H NMR (500 MHz, acetone-*d*_6_) spectrum of compound **65**.

**Figure S132.** ^13^C NMR (125 MHz, acetone-*d*_6_) spectrum of compound **65**.

**Figure S133.** HRESIMS spectrum of compound **65**.

**Figure S134.** ^1^H NMR (500 MHz, acetone-*d*_6_) spectrum of compound **66**.

**Figure S135.** ^13^C NMR (125 MHz, acetone-*d*_6_) spectrum of compound **66**.

**Figure S136.** HRESIMS spectrum of compound **66**.

**Figure S137.** ^1^H NMR (600 MHz, CDCl_3_) spectrum of compound **67**.

**Figure S138.** ^13^C NMR (150 MHz, CDCl_3_) spectrum of compound **67**.

**Figure S139.** HRESIMS spectrum of compound **67**.

**Figure S140.** ^1^H NMR (500 MHz, acetone-*d*_6_) spectrum of compound **68**.

**Figure S141.** ^13^C NMR (125 MHz, acetone-*d*_6_) spectrum of compound **68**.

**Figure S142.** HRESIMS spectrum of compound **68**.

**Figure S143.** ^1^H NMR (500 MHz, acetone-*d*_6_) spectrum of compound **69**.

**Figure S144.** ^13^C NMR (125 MHz, acetone-*d*_6_) spectrum of compound **69**.

**Figure S145.** HRESIMS spectrum of compound **69**.

**Figure S146.** ^1^H NMR (500 MHz, acetone-*d*_6_) spectrum of compound **70**.

**Figure S147.** HRESIMS spectrum of compound **70**.

**Figure S148.** ^1^H NMR (500 MHz, acetone-*d*_6_) spectrum of compound **71**.

**Figure S149.** ^13^C NMR (125 MHz, acetone-*d*_6_) spectrum of compound **71**.

**Figure S150.** HRESIMS spectrum of compound **71**.

**Figure S151.** ^1^H NMR (500 MHz, acetone-*d*_6_) spectrum of compound **72**.

**Figure S152.** ^13^C NMR (125 MHz, acetone-*d*_6_) spectrum of compound **72**.

**Figure S153.** HRESIMS spectrum of compound **72**.

**Figure S154.** ^1^H NMR (500 MHz, acetone-*d*_6_) spectrum of compound **73**.

**Figure S155.** ^13^C NMR (125 MHz, acetone-*d*_6_) spectrum of compound **73**.

**Figure S156.** ESIMS spectrum of compound **73**.

**Figure S157.** ^1^H NMR (500 MHz, acetone-*d*_6_) spectrum of compound **74**.

**Figure S158.** ESIMS spectrum of compound **74**.

**Figure S159.** ^1^H NMR (500 MHz, acetone-*d*_6_) spectrum of compound **75**.

**Figure S160.** ^13^C NMR (125 MHz, acetone-*d*_6_) spectrum of compound **75**.

**Figure S161.** HRESIMS spectrum of compound **75**.

**Figure S162.** ^1^H NMR(500 MHz, acetone-*d*_6_) spectrum of compound **76**.

**Figure S163.** ^13^C NMR (125 MHz, acetone-*d*_6_) spectrum of compound **76**.

**Figure S164.** HRESIMS spectrum of compound **76**.

**Figure S165.** ^1^H NMR (600 MHz, CDCl_3_) spectrum of compound **77**.

**Figure S166.** ^13^C NMR (150 MHz, CDCl_3_) spectrum of compound **77**.

**Figure S167.** HRESIMS spectrum of compound **77**.

**Figure S168.** ^1^H NMR (500 MHz, acetone-*d*_6_) spectrum of compound **78**.

**Figure S169.** ^13^C NMR (125 MHz, acetone-*d*_6_) spectrum of compound **78**.

**Figure S170.** HRESIMS spectrum of compound **78**.

**Figure S171.** ^1^H NMR (500 MHz, acetone-*d*_6_) spectrum of compound **79**.

**Figure S172.** ^13^C NMR (125 MHz, acetone-*d*_6_) spectrum of compound **79**.

**Figure S173.** HRESIMS spectrum of compound **79**.

**Figure S174.** ^1^H NMR (500 MHz, acetone-*d*_6_) spectrum of compound **80**.

**Figure S175.** ^13^C NMR (125 MHz, acetone-*d*_6_) spectrum of compound **80**.

**Figure S176.** ESIMS spectrum of compound **80**.

**Figure S177.** ^1^H NMR (500 MHz, acetone-*d*_6_) spectrum of compound **81**.

**Figure S178.** ^13^C NMR (125 MHz, acetone-*d*_6_) spectrum of compound **81**.

**Figure S179.** HRESIMS spectrum of compound **81**.

**Figure S180.** ^1^H NMR (500 MHz, acetone-*d*_6_) spectrum of compound **82**.

**Figure S181.** ^13^C NMR (125 MHz, acetone-*d*_6_) spectrum of compound **82**.

**Figure S182.** ESIMS spectrum of compound **82**.

**Figure S183.** ^1^H NMR (500 MHz, acetone-*d*_6_) spectrum of compound **83**.

**Figure S184.** ^13^C NMR (125 MHz, acetone-*d*_6_) spectrum of compound **83**.

**Figure S185.** HRESIMS spectrum of compound **83**.

**Figure S186.** ^1^H NMR (500 MHz, acetone-*d*_6_) spectrum of compound **84**.

**Figure S187.** ^13^C NMR (125 MHz, acetone-*d*_6_) spectrum of compound **84**.

**Figure S188.** HRESIMS spectrum of compound **84**.

**Figure S189.** ^1^H NMR (500 MHz, acetone-*d*_6_) spectrum of compound **85**.

**Figure S190.** ^13^C NMR (125 MHz, acetone-*d*_6_) spectrum of compound **85**.

**Figure S191.** HRESIMS spectrum of compound **85**.

**Figure S192.** ^1^H NMR (500 MHz, acetone-*d*_6_) spectrum of compound **86**.

**Figure S193.** ^13^C NMR (125 MHz, acetone-*d*_6_) spectrum of compound **86**.

**Figure S194.** ESIMS spectrum of compound **86**.

**Figure S195.** ^1^H NMR (500 MHz, acetone-*d*_6_) spectrum of compound **87**.

**Figure S196.** ^13^C NMR (125 MHz, acetone-*d*_6_) spectrum of compound **87**.

**Figure S197.** ESIMS spectrum of compound **87**.

**Figure S198.** ^1^H NMR (600 MHz, CDCl_3_) spectrum of compound **88**.

**Figure S199.** ^13^C NMR (150 MHz, CDCl_3_) spectrum of compound **88**.

**Figure S200.** HRESIMS spectrum of compound **88**.

**Figure S201.** ^1^H NMR (600 MHz, CDCl_3_) spectrum of compound **89**.

**Figure S202.** ^13^C NMR (150 MHz, CDCl_3_) spectrum of compound **89**.

**Figure S203.** HRESIMS spectrum of compound **89**.

**Figure S204.** ^1^H NMR (600 MHz, CDCl_3_) spectrum of compound **90**.

**Figure S205.** ^13^C NMR (150 MHz, CDCl_3_) spectrum of compound **90**.

**Figure S206.** HRESIMS spectrum of compound **90**.

**Figure S207.** ^1^H NMR (500 MHz, acetone-*d*_6_) spectrum of compound **91**.

**Figure S208.** ^13^C NMR (125 MHz, acetone-*d*_6_) spectrum of compound **91**.

**Figure S209.** HRESIMS spectrum of compound **91**.

**Figure S210.** ^1^H NMR (600 MHz, CDCl_3_) spectrum of compound **92**.

**Figure S211.** ^13^C NMR (150 MHz, CDCl_3_) spectrum of compound **92**.

**Figure S212.** HRESIMS spectrum of compound **92**.

**Figure S213.** ^1^H NMR (500 MHz, acetone-*d*_6_) spectrum of compound **93**.

**Figure S214.** ^13^C NMR (500 MHz, acetone-*d*_6_) spectrum of compound **93**.

**Figure S215.** ESI-MS spectrum of compound **93**.

**Figure S216.** ^1^H NMR (500 MHz, acetone-*d_6_*) spectrum of compound **94**.

**Figure S217.** ^13^C NMR (125 MHz, acetone-*d_6_*) spectrum of compound **94**.

**Figure S218.** HRESIMS spectrum of compound **94**.

**Figure S219.** ^1^H NMR (400 MHz, CDCl_3_) spectrum of compound **95**.

**Figure S220.** ^13^C NMR (100 MHz, CDCl_3_) spectrum of compound **95**.

**Figure S221.** HRESIMS spectrum of compound **95**.

**Figure S222.** ^1^H NMR (500 MHz, acetone-*d*_6_) spectrum of compound **96**.

**Figure S223.** ^13^C NMR (125 MHz, acetone-*d*_6_) spectrum of compound **96**.

**Figure S224.** HRESIMS spectrum of compound **96**.

**Figure S225.** ^1^H NMR (600 MHz, CDCl_3_) spectrum of compound **97**.

**Figure S226.** ^13^C NMR (150 MHz, CDCl_3_) spectrum of compound **97**.

**Figure S227.** HRESIMS spectrum of compound **97**.

**Figure S228.** ^1^H NMR (500 MHz, acetone-*d*_6_) spectrum of compound **98**.

**Figure S229.** ^13^C NMR (125 MHz, acetone-*d*_6_) spectrum of compound **98**.

**Figure S230.** HRESIMS spectrum of compound **98**.

**Figure S231.** ^1^H NMR (500 MHz, acetone-*d*_6_) spectrum of compound **99**.

**Figure S232.** ^13^C NMR (125 MHz, acetone-*d*_6_) spectrum of compound **99**.

**Figure S233.** HRESIMS spectrum of compound **99**.

**Figure S234.** ^1^H NMR (500 MHz, acetone-*d*_6_) spectrum of compound **100**.

**Figure S235.** ^13^C NMR (125 MHz, acetone-*d*_6_) spectrum of compound **100**.

**Figure S236.** HRESIMS spectrum of compound **100**.
